# Nitrogen‐Rich 5,5′‐Azotetrazolates: A Broad Spectrum of Technologically Accessible Gas‐Generating Pyrotechnic Materials

**DOI:** 10.1002/open.70288

**Published:** 2026-08-31

**Authors:** Adam Votýpka, Robert Matyáš, Jakub Mikuláštík, Aleš Růžička, Zdeňka Růžičková, Jan Zigmund, Miroslav Labaj, Martin Adam, Zdeněk Jalový

**Affiliations:** ^1^ Institute of Energetic Materials Faculty of Chemical Technology University of Pardubice Pardubice Czech Republic; ^2^ Department of General and Inorganic Chemistry Faculty of Chemical Technology University of Pardubice Pardubice Czech Republic; ^3^ Department of Analytical Chemistry Faculty of Chemical Technology University of Pardubice Pardubice Czech Republic

**Keywords:** azo compounds, energetic materials, gas generate compositions, green chemistry, thermochemistry

## Abstract

Ten 5,5′‐azotetrazolate salts with nitrogen‐rich bases was prepared and evaluated as technologically accessible gas‐generating materials. In addition to sufficient performance, the development focused on safety, reliable handling, and environmental responsibility, as essential factors for evaluating compounds intended for controlled gas generation. The azo and tetrazole framework combines high nitrogen content with favorable energetic characteristics, making it an attractive platform for applications requiring rapid, controlled gas release. The prepared salts were evaluated for their physicochemical and safety‐relevant properties, including bulk density, hygroscopicity, and mechanical sensitivity. The structures of several compounds were determined by X‐ray analysis of single‐crystalline material. The ballistic properties were calculated for both the compounds themselves and their mixtures with the oxidizers potassium perchlorate and strontium nitrate, which ensure the required reaction rate while producing nontoxic gaseous and solid products. Several compounds showed advantageous combinations of high bulk density, low‐to‐moderate hygroscopicity, and favorable impact and friction sensitivities, resulting in performance comparable to that of currently used materials or commonly cited high‐potential candidates for gas‐generating components in various safety systems.

## Graphical abstract

A series of ten 5,5′‐azotetrazolate salts with nitrogen‐rich bases is prepared and assessed as technologically accessible gas‐generating materials. Structural, safety, and ballistic evaluations reveal several compounds combining high bulk density, acceptable sensitivities, and favorable gas‐generation performance, highlighting the potential of azotetrazolate‐based systems for environmentally responsible gas‐generating applications.

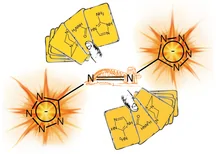

## 1 Introduction

Gas‐generating pyrotechnic compositions are used in systems that require rapid, controlled, and reliable gas generation to perform mechanical functions in equipment, including inflators, separation devices, and pyrotechnic actuators. The most widely known class of gas‐generating compositions is those used in automotive airbag inflators [1, 2]. Airbag‐based protection systems have a surprisingly long history, originating in the early twentieth century when Parrot and Round patented protective “air cushions” for vehicles [3]. Their real and broad technological expansion, however, began in the latter half of the twentieth century [4] and originally used gas cylinders [5, 6] were, to a great extent, replaced by gas‐generating pyrotechnic compositions capable of producing the required pressure within tenths of a millisecond [7]. Among the many mixtures explored, sodium azide‐based compositions were the first to be widely implemented. They provided reliable performance for decades and produced predominantly nitrogen gas during combustion [1, 7]. However, sodium azide has several disadvantages, the most notable of which is its toxicity [4], significant environmental impact [8], relatively low specific gas yield [9], complex and unresolved disposal issues [9], and the risk of misuse of undeployed modules for improvised synthesis of primary explosives such as lead azide [10].

In the second generation of airbag compositions, sodium azide was replaced by nitrogen‐rich organic compounds whose thermal decomposition likewise produces nitrogen as the predominant gaseous product. Among the most widely applied were azodicarbonamide, guanidine nitrate, nitroguanidine, and triaminoguanidine nitrate [9, 11, 12, 13]. These materials offer the advantages of broad availability and favorable combustion product profiles; however, their relatively low heat of formation limits their energetic performance.

Compared with conventional air bag compositions or gas‐generating pyrotechnic compositions in general, tetrazole‐based compounds offer distinct advantages. Their high nitrogen content and corresponding low carbon content in the tetrazole molecule provide an exceptionally favorable oxygen balance (OB), enabling minimal oxidizer content in gas‐generating pyrotechnic composition. Consequently, their combustion produces predominantly nitrogen gas at comparatively low temperatures [14], a highly desirable feature for airbag inflators and related applications. A further benefit is their energetic character; tetrazoles possess high positive heats of formation (+237 kJ mol^−1^ for 5*H*‐1,2,3,4‐tetrazole [15]). In addition, the aromatic nature of the tetrazole ring confers notable chemical and thermal stability [16, 17]. Together, these attributes make the tetrazole scaffold an exceptionally attractive platform for the design of modern gas‐generating compositions.

In recent decades, numerous tetrazole derivatives have been proposed for application in gas‐generating compositions [18, 19, 20, 21, 22]. However, the large‐scale production of most of these compounds remains technologically challenging. The principal limitations arise from their limited availability (high production costs and a multistep synthesis process) and significant safety concerns associated with their manufacture. Particularly problematic and hazardous on the technological scale is the diazotization of 5‐aminotetrazole, a key precursor for the synthesis of numerous tetrazole‐based compounds. For example, the diazotization of 5‐aminotetrazole to sodium 5‐nitrotetrazolate, an essential precursor for various primary explosives as well as nitrogen‐rich salts of nitrogen bases [23, 24], is known to be accompanied by microdetonations and to form highly hazardous intermediate [25, 26, 27, 28]. Although significant progress has been made in diazotization technology in recent years, it remains difficult on a large scale [29, 30, 31, 32, 33].

**FIGURE 1:**
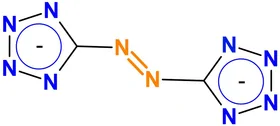
Tetrazole and azo groups as energetic building blocks of *N*‐rich 5,5′‐azotetrazolate compounds.

A promising framework for the design of high‐nitrogen gas‐generating propellants is the 5,5′‐azotetrazolate moiety, which combines a heterocyclic high‐nitrogen tetrazole ring with a highly energetic azo group [14] (Figure 1). 5,5′‐Azotetrazole is stable only in the form of its salts [34] that can be prepared both in laboratory and technological scale by oxidizing 5‐aminotetrazole using a range of agents, most commonly potassium permanganate [35, 36, 37, 38]. This method is technologically undemanding, safe, and has been well established over decades of industrial practice. Only a limited number of readily accessible salts of nitrogen‐rich bases—primarily guanidinium, triaminoguanidinium, and azidoformamidinium azotetrazolates—have been reported, some of which have been proposed for use in gas‐generating compositions [14, 34, 39, 40].

The present study focuses on developing high‐performance energetic materials based on salts of 5,5′‐azotetrazolate, with nitrogen‐rich bases that offer a good balance between physicochemical and energetic properties and an easy manufacturing process. The compounds contain structural motifs such as glyoxal–amidino guanidine cations, amidino‐guanidine‐amino‐triazole frameworks, and linked triazole units that, to the best of our knowledge, have not been described previously. Furthermore, all studied compounds crystallize with favorable morphologies and exhibit relatively high bulk densities without the use of crystal modifiers. Together with the comprehensive characterization presented herein, as well as the evaluation of ballistic parameters and combustion products in real pyrotechnic compositions, these features provide a basis for identifying the most promising candidates for specific gas‐generation systems.

## 2 Experimental Part

The preparation of the starting sodium salt of 5,5′‐azotetrazole, all investigated *N*‐rich salts of 5,5′‐azotetrazole, and the reference materials guanidinium azotetrazolate (GZT), triaminoguanidinium azotetrazolate (TAGZT), and triaminoguanidinium nitrate (TAGN), along with their analytical characterization, is described in the Supporting Information. Detailed descriptions of all methods used to determine individual parameters are also provided therein.

## 3 Results and Discussion

### 3.1 Synthesis

**FIGURE 2:**
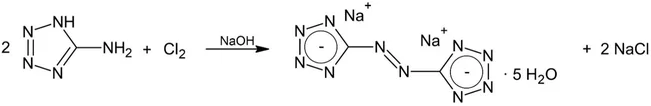
Synthesis of sodium 5,5′‐azotetrazolate pentahydrate.

The salts of 5,5′‐azotetrazole were prepared from the sodium 5,5′‐azotetrazolate pentahydrate, which is mostly prepared by the Thiele method [35], i.e., by oxidation of 5‐aminotetrazole with potassium permanganate [14, 36, 37, 38]. A significant drawback of this procedure is the formation of manganese dioxide as a by‐product, which precipitates as an extremely fine, poorly filterable solid, thereby complicating the technological‐scale production of sodium 5,5‐azotetrazolate. Therefore, we apply chlorine as a promising alternative oxidizing agent (Figure 2). The strain‐forward use of chlorine yields the product with a remarkably high 71% yield, comparable to that of the original Thiele method. The nontoxic by‐product, sodium chloride, remains dissolved in the aqueous reaction medium, allowing the slightly soluble sodium salt of 5,5′‐azotetrazole to be readily isolated from the reaction mixture. The replacement of manganese dioxide with chlorine significantly facilitates the large‐scale production of the sodium salt of 5,5′‐azotetrazole.

**FIGURE 3:**
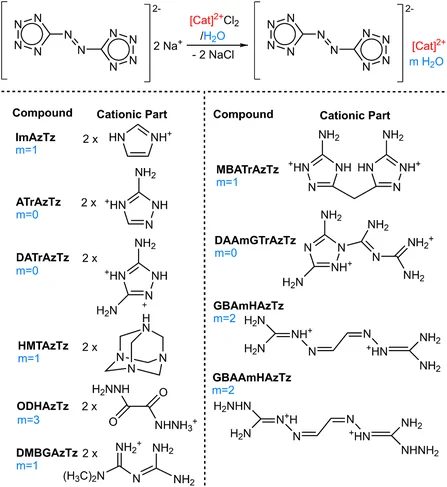
Preparation and structures of energetic salts of 5,5′‐azotetrazole.

Salts of 5,5‐azotetrazole with: imidazole **ImAzTz·H**_**2**_ **O**, derivatives of aminotriazole **ATrAzTz**, **DATrAzTz**, **MBATrAzTz·H**_**2**_ **O**, and **DAAmGTrAzTz**, hexamine **HMTAzTz·H**_**2**_ **O**, oxalyl dihydrazide **ODHAzTz·3H**_**2**_ **O**, and guanidine derivatives **DMBGAzTz·H**_**2**_ **O**, **GBAmHAzTz·2H**_**2**_ **O**, and **GBAAmHAzTz·2H**_**2**_ **O** were prepared by precipitating from an aqueous solution of sodium 5,5′‐azotetrazolate with hydrochlorides of the corresponding nitrogen bases (Figure 3). The reactions are simple to perform, do not require any technologically demanding steps, and yield a product that precipitates in an easily filterable form. Moreover, all compounds were obtained in high yields in the range from 88% to 99% (Supporting Information: Synthesis).

### 3.2 Crystal Structure

Of the ten compounds, five yielded single crystals suitable for molecular structure determination by scXRD techniques. The crystals were grown using the silica‐gel method (Supporting Information: Synthesis). For compounds **HMTAzTz**, **DMBGAzTz**, and **GBAmHAzTz**, the techniques used for single‐crystal growth differ from those for bulk synthesis, whereas the resulting single crystals differ in their crystalline water content from the bulk materials.

**FIGURE 4:**
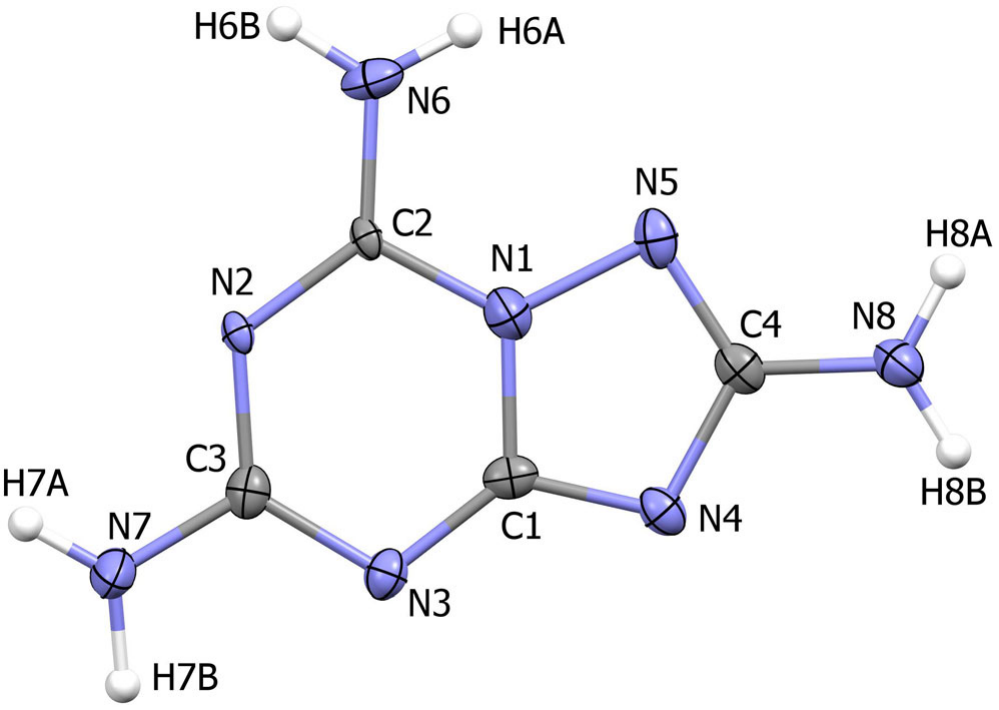
Molecular structure of the condensation product of **DAAmGTrAzTz**. Ortep view, 50% probability level, minor disorder of N2 and C2 atoms is not shown. Selected interatomic distances [Å] and angles [°]: N1—C2 1.355(5), N1—C1 1.383(5), N1—N5 1.407(5), N3—C1 1.337(5), N3—C3 1.361(5), N4—C1 1.318(5), N4—C4 1.373(5), N5—C4 1.313(5), N6—C2 1.348(5), N7—C3 1.334(5), N8—C4 1.342(5), C1—N2A 1.445(14), C3—N2 1.346(5), N2—C2 1.349(5).

Four of the azotetrazolates, such as **DATrAzTz**, **HMTAzTz**, **DMBGAzTz**, and **GBAmHAzTz**, exhibit the structure containing a protonated nitrogen‐rich moiety present in the cationic part of the molecule, thus compensating for the negative charge located on the azotetrazolate part. The fifth molecular structure is the product of the 3,5‐diamino‐*N*‐amidino‐1‐guanyl‐1,2,4‐triazole **DAAmGTrAzTz** condensation, upon formation of ammonia, to the [1, 2, 4]triazolo[1,5‐a] [1, 3, 5] triazine‐2,5,7‐triamine, which is already described in its protonated form as perchlorate [41]. For this compound, the condensation typically occurs under the aqueous conditions used for single‐crystal growth in silica gel, while it is not observed in the bulk material synthesized in ethanol [42]. Only negligible elongations of C1—N4 and C2—N2 bonds by ~0.03 Å are observed in the protonated reported structure in comparison to the condensation product (Figure 4).

**FIGURE 5:**
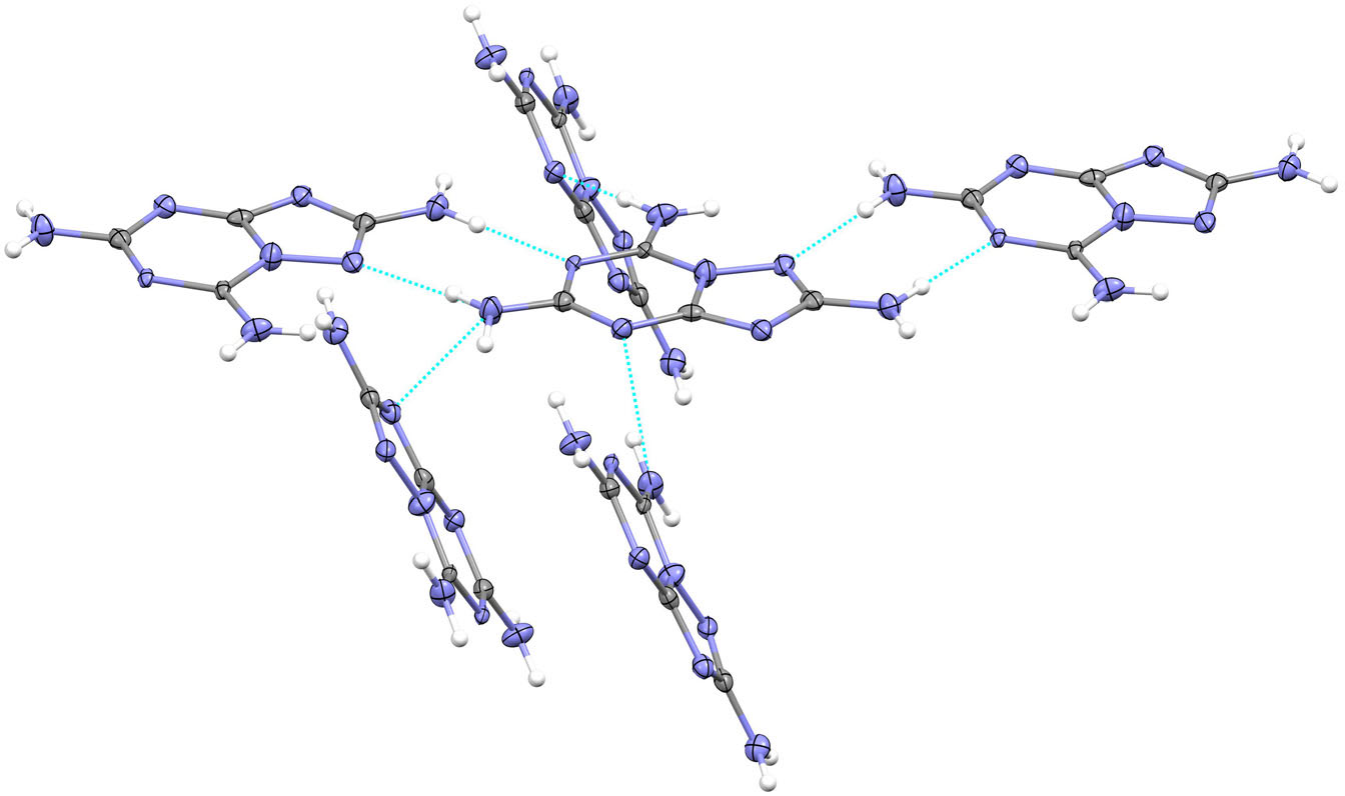
Dominant H‐bonding (Ortep view, 50% probability level) in [1,2,4]triazolo[1,5‐a][1,3,5]triazine‐2,5,7‐triamine.

In contrast to its protonated form [41], the [1, 2, 4]triazolo[1,5‐a] [1, 3, 5] triazine‐2,5,7‐triamine exhibits many more degrees of freedom to form hydrogen bonds and short contacts owing to the absence of an anion. The dominant types are contacts of N8—H8B…N2 and N7—H7A…N5 promoting the formation of a planar chain of molecules, while the rest of the NH bonds are involved in bonding of nearly perpendicularly bonded molecules (Figure 5 and Table S9). The *π*–*π* stacking is less pronounced.

**FIGURE 6:**
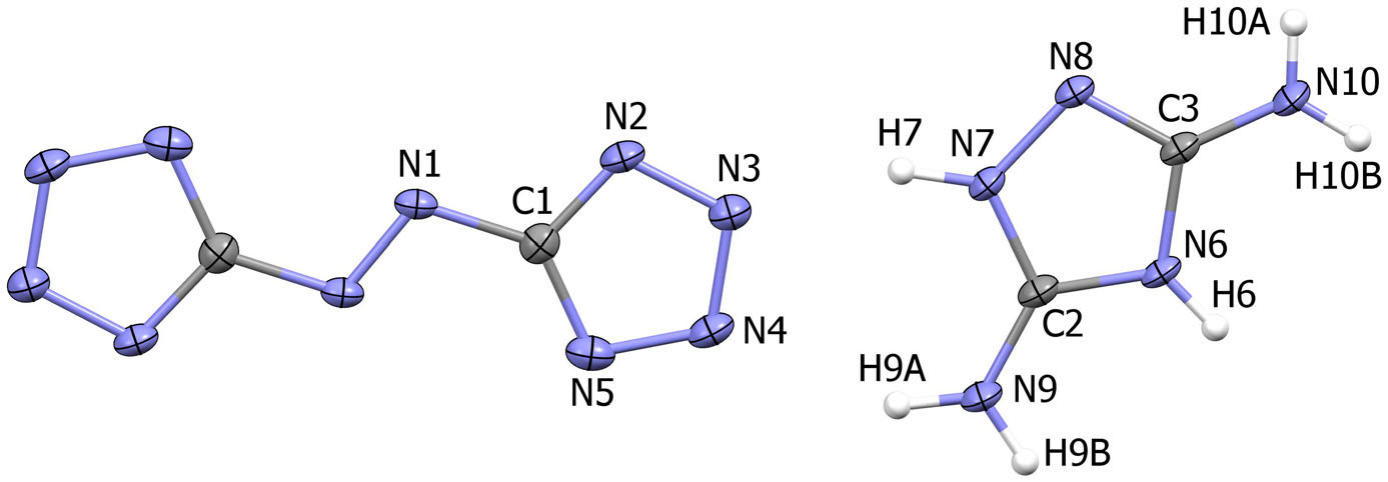
Molecular structure of **DATrAzTz**. Ortep view, 50% probability level, minor disorder of N1 atom is not shown. Selected interatomic distances [Å] and angles [°]: N2—C1 1.315(3), N2—N3 1.328(2), N3—N4 1.322(2), N4—N5 1.333(3), N5—C1 1.341(3), N6—C2 1.349(3), N6—C3 1.381(3), N7—C2 1.325(3), N7—N8 1.393(2), N8—C3 1.315(3), N9—C2 1.318(3), N10—C3 1.327(3), C1—N1 1.416(7), N1—N1*^i^* 1.264(10).

**FIGURE 7:**
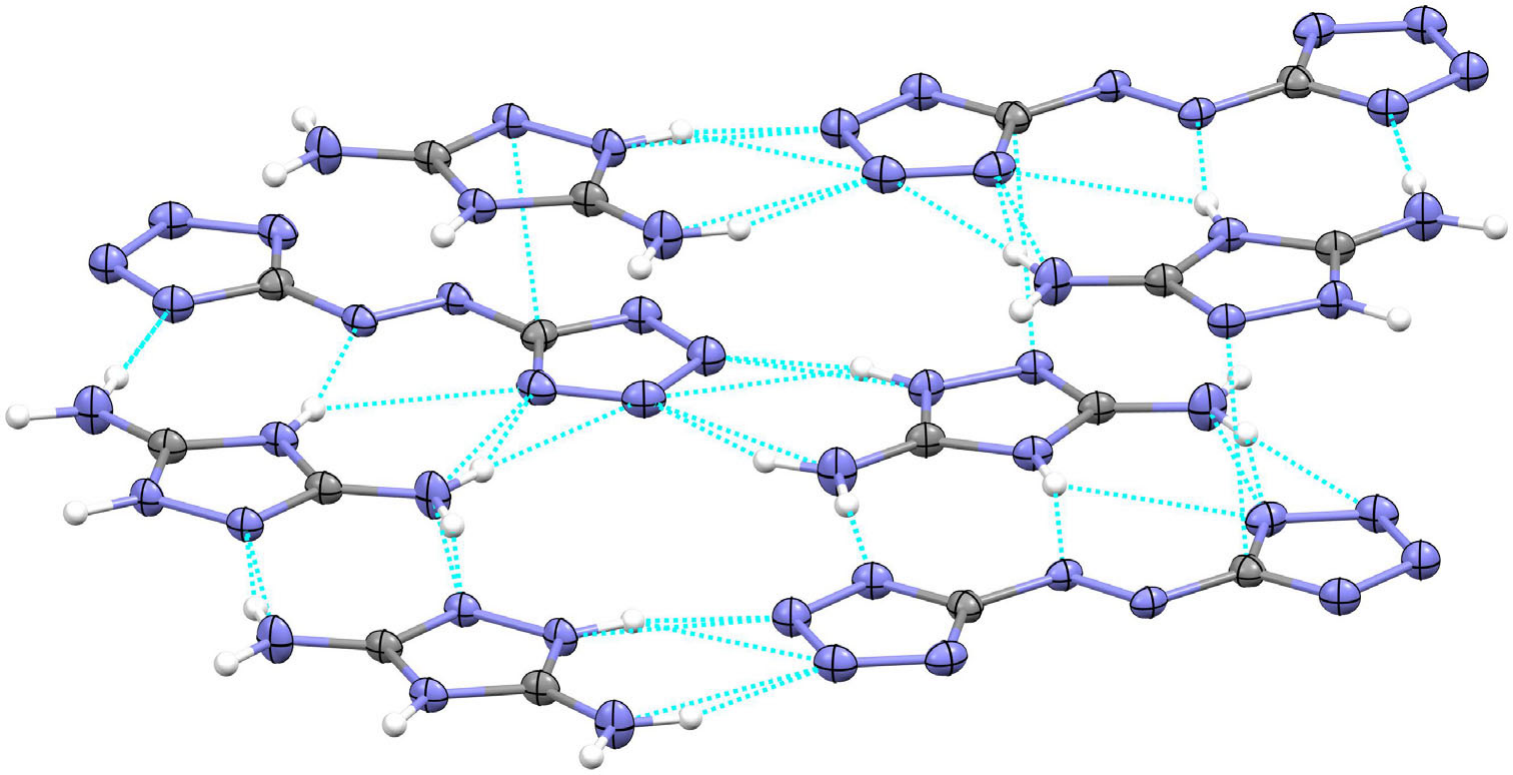
H‐bonding patterns (Ortep view, 50% probability level) in **DATrAzTz**.

In all dianionic azotetrazolate units of **DATrAzTz**, **HMTAzTz**, **DMBGAzTz**, and **GBAmHAzTz**, the geometry is planar, and the N=N separation of the azenyl group is of typical value ~1.26 Å. Due to the absence of a solvent or water molecule, the compound **DATrAzTz** crystallizes as a layered structure with a planar azotetrazolate anion and 3,5‐diamino‐4*H*−1,2,4‐triazol‐1‐ium cation (Figure 6 and Table S10). This particular architecture is stabilized by extensive H‐bonding, where several rings between both heterocycles were identified: the thirteen‐membered by N10H10B…N5 and N9H9B…N2, supported by close contact N6H6…N1; the five‐membered by N7H7…N3 and N9H9A…N4; the eight‐membered by two N10H10A…N8; and the second thirteen‐membered by N10H10A…N8, N10H10B…N5, N7H7…N3, N9H9A…N4, and N9H9B…N2. Each layer is interconnected by *π*–*π* stacking formed from dominant areas around the N8 and C1 atoms (Figure 7 and Table S11).

**FIGURE 8:**
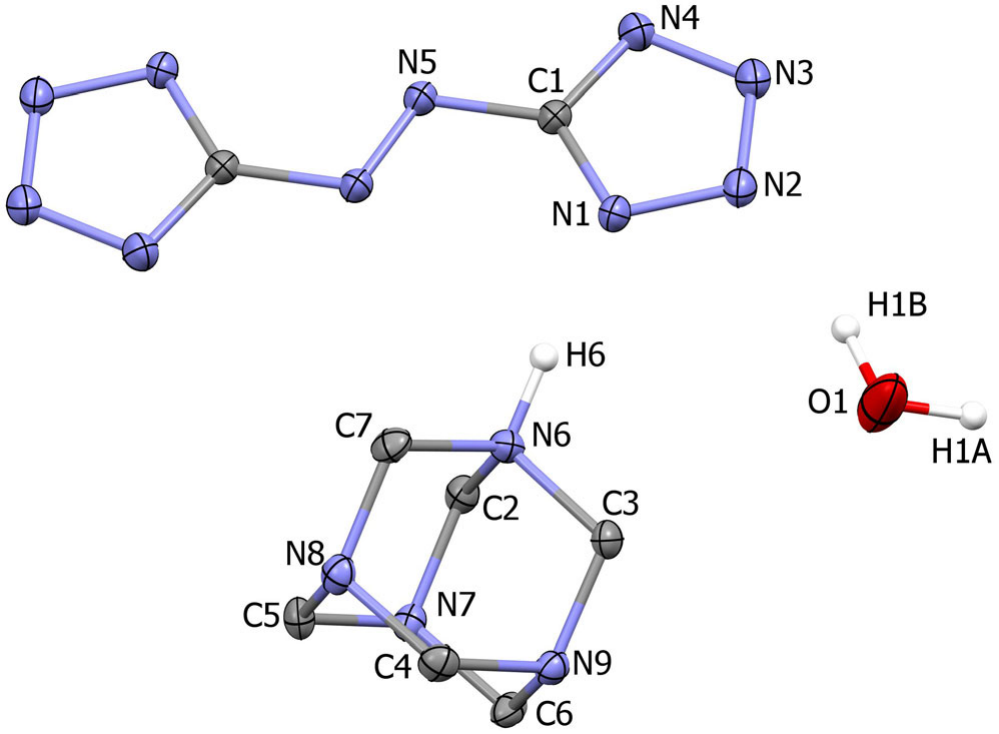
Molecular structure of **HMTAzTz·2H** **_2_** **O**. Ortep view, 50% probability level. Selected interatomic distances [Å] and angles [°]: N1—N2 1.3340(16), N1—C1 1.3366(17), N2—N3 1.3253(18), N3—N4 1.3367(17), N4—C1 1.3330(17), N5—N5*^i^* 1.258(2), N5—C1 1.4054(16), N6—C2 1.5143(16), N6—C7 1.5175(17), N6—C3 1.5182(16), N7—C2 1.4395(17), N7—C6 1.4721(16), N7—C5 1.4741(16), N8—C7 1.4475(17), N8—C5 1.4736(17), N8—C4 1.4743(17), N9—C3 1.4460(17), N9—C4 1.4738(17), N9—C6 1.4749(16).

**FIGURE 9:**
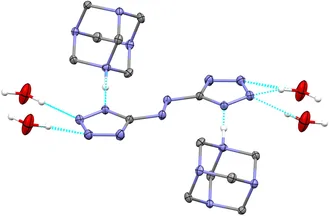
H‐bonding patterns (Ortep view, 50% probability level) in **HMTAzTz·2H** **_2_** **O**.

The molecular structure of **HMTAzTz** consists of dianionic azotetrazolate and two urotopinium cations connected (Figure 8 and Table S12) by N6H6…N1 H‐bonds. Other contacts, important for the creation of a layered structure, are promoted by the presence of two water molecules interacting with N2 and N3 atoms. Several weak interactions between nitrogen atoms and methylene hydrogens support either the layers or interconnect them (Figure 9 and Table S13).

**FIGURE 10:**
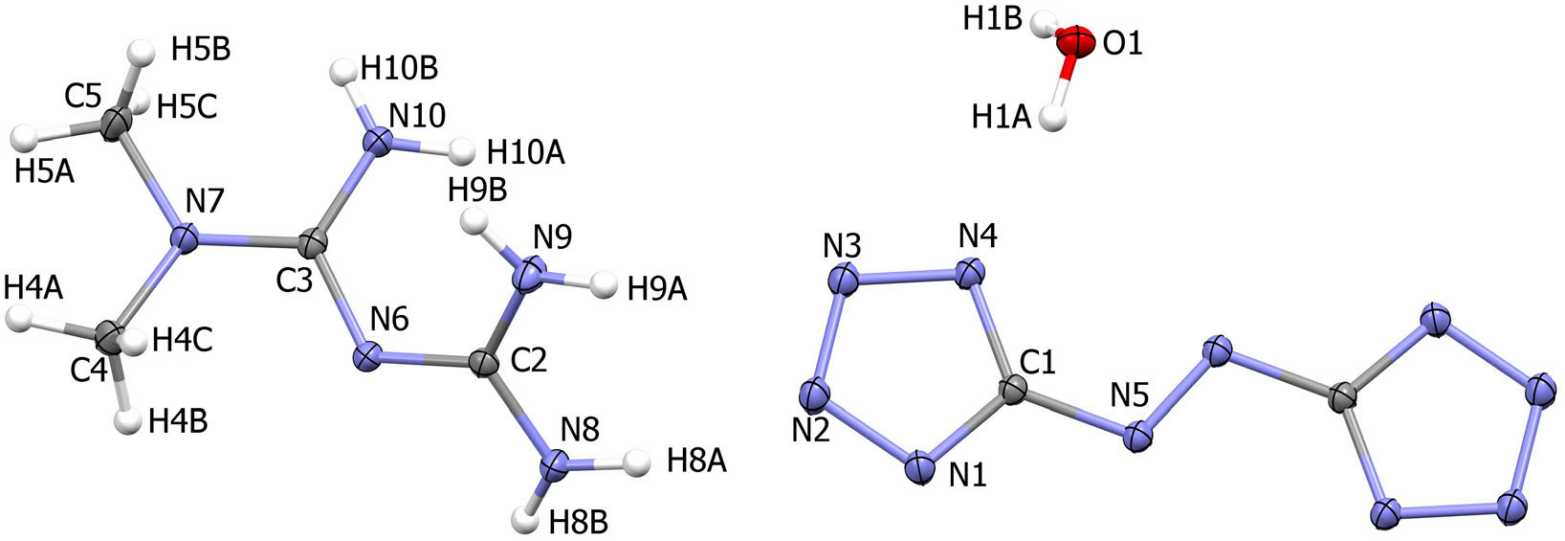
Molecular structure **DMBGAzTz·2H** **_2_** **O**. Ortep view, 50% probability level. Selected interatomic distances [Å] and angles [°]: N1—C1 1.3354(18), N1—N2 1.3366(18), N2—N3 1.3226(19), N3—N4 1.3404(18), N4—C1 1.3382(18), N5—N5*^i^* 1.259(2), N5—C1 1.4010(18), N6—C2 1.3375(18), N6—C3 1.3473(18), N7—C3 1.3398(18), N7—C5 1.4563(19), N7—C4 1.4579(19), N8—C2 1.3310(19), N9—C2 1.334(2), N10—C3 1.3326(19).

**FIGURE 11:**
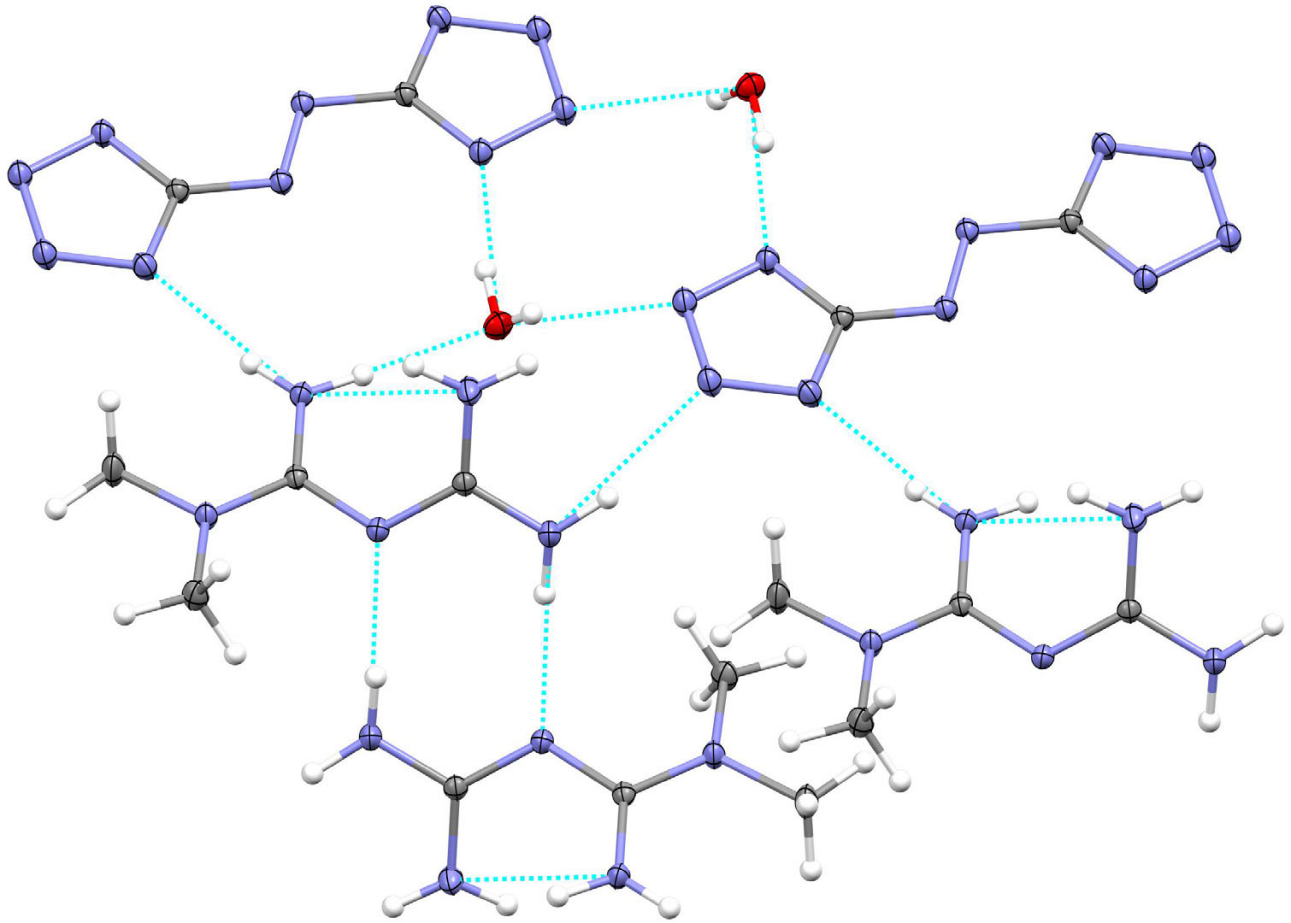
H‐bonding patterns (Ortep view, 50% probability level) in **DMBGAzTz·2H** **_2_** **O**.

In **DMBGAzTz**, two protonated 1,1‐dimethylbiguanides compensate azotetrazolate(2‐) ion (Figure 10), water molecules interconnect the N4 atom with N3 form another anion, and two distorted biguanidinium units are joined by N8H8B…N6 to a six‐membered ring, while remaining H atoms point to the azotetrazolate (Figure 11 and Tables S14 and S15).

**FIGURE 12:**
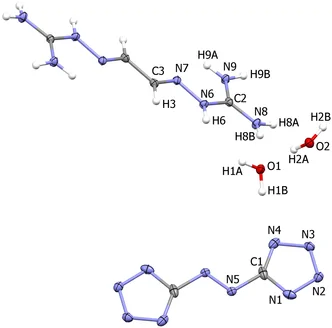
Molecular structure of **GBAmHAzTz·4H** **_2_** **O**. Ortep view, 50% probability level. Selected interatomic distances [Å] and angles [°]: N1—C1 1.3244(19), N8—C2 1.3202(16), N1—N2 1.3262(16), N9—C2 1.3152(16), N2—N3 1.3150(16), C1—N5 1.451(6), N3—N4 1.3248(17), N4—C1 1.3219(19), C3—C3*ii* 1.442(2), N6—C2 1.3504(15), N5—N5*i* 1.263(10), N6—N7 1.3611(14), N7—C3 1.2846(15), C1—N1—N2 104.20(10), N3—N2—N1 109.41(10), N2—N3—N4 109.63(10), C1—N4—N3 104.21(11), N9—C2—N8 122.62(11), C2—N6—N7 118.46(9), N9—C2—N6 120.13(11), C3—N7—N6 116.06(10), N8—C2—N6 117.25(10), N4—C1—N1 112.54(11), N7—C3—C3*ii* 117.92(13), N4—C1—N5 143.2(2), N5*i*—N5—C1 107.5(4), N1—C1—N5 104.11(19). Symmetry codes: (i) −*x* + 1, −*y* + 2, −*z* + 1; (ii) −*x* + 1, −*y*, −*z*.

**FIGURE 13:**
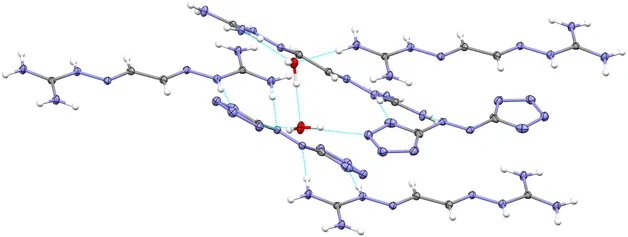
H‐bonding patterns (Ortep view, 50% probability level) in **GBAmHAzTz·4H** **_2_** **O**.

The molecular structure of **GBAmHAzTz** is composed of azotetrazolate dianion, bis(amidinohydrazonium)glyoxal dication, and four water molecules. The charged parts are interconnected by N6H6…N1 and N8H8B…N5 (from the azenyl group) H‐bridges. Two water molecules serve as electron density donors for cations (N9H9A…O2 and N8H8A…O2), while another two serve as acceptors joining azotetrazolates (O1H1A…N2 and O1H1B…N4). The remaining nitrogen donors of the negatively charged part interact with the hydrogens of water molecules (Figures 12 and 13, Tables S16 and S17).

### 3.3 Physicochemical Properties

Standard solid‐state enthalpies of formation are essential parameters for evaluating and predicting energetic performance. These values were determined using both computational and experimental methods, as shown in Table 1.

**TABLE 1:** The fundamental physical properties of synthesized compounds and reference substances GZT, TAGZT, and TAGN.

Compound	*M*, g mol^−^^1^ a	*ρ*, g cm^−^^3^ b	Δ_f_ *H* ^°^, kJ mol^−1^ c	Δ_f_ *H* ^°^, kJ mol^−1^ d	*T*_d_,°C^e^
ImAzTz·H_2_O	320.3	1.450	712.3	515.6	164
ATrAzTz	334.3	1.634	1054	958.4	163
DATrAzTz	364.3	1.630	825.3	885.7	195
MBATrAzTz·H_2_O	364.3	1.715	741.7	412.4	134
DAAmGTrAzTz	349.3	1.593	739.9	594.3	171
HMTAzTz·H_2_O	464.5	1.593	138.3	891.4	149
ODHAzTz·3H_2_O	456.3	1.442	−736.1	−328.5	85
DMBGAzTz·H_2_O	442.5	1.604	−4.763	315.8	182
GBAmHAzTz·2H_2_O	372.3	1.352	411.9	103.4	185
GBAAmHAzTz·2H_2_O	402.4	1.590	563.0	293.3	160
GZT	284.3	1.569 [43]	410.0 [14]	459.8	215
TAGZT	374.3	1.634 [43]	1062 [14]	1069	180
TAGN	167.1	1.594 [44]	−48.12 [45]	−120.1	216

a Molar mass.

b Densities determined by pycnometry.

c Experimentally determined enthalpy of formation.

d Calculated enthalpy of formation.

e Onset decomposition temperature.

The theoretical values calculated by quantum chemical calculations and empirical volume‐based thermodynamics (VBT) for the anhydrous salts **ATrAzTZ**, **DATrAzTz,** and **DAAmGTrAzTz** are in rather good agreement with the experimental data (Table 1). For more complex systems such as hydrates, these computational models, specifically VBT estimations, show substantial discrepancies. The reason is that water of crystallization significantly restructures the intermolecular hydrogen‐bonding networks in ways not captured by additive empirical constants. A notably higher theoretical overestimation is observed for compound **HMTAzTz·H**_**2**_ **O** (calc. 891.4 vs. exp. 138.3 kJ mol^−1^), as standard models parameterized for planar ions struggle to accurately account for the strain and unique lattice packing of bulky, 3D cage structures.

The second method we used to determine the enthalpies was bomb calorimetry, which avoids computational limitations, and we preferred it for calculating ballistic parameters. Based on calorimetric data, the experimental values range from −736.1 kJ mol^−1^ for the trihydrate **ODHAzTz·3H**_**2**_ **O** (given by the strong stabilizing hydrogen‐bond network of its oxygen‐rich cation) to highly positive values reaching 1054 kJ mol^−1^ for **ATrAzTz**. To put the energetic potential of the synthesized compounds in context, the results were compared against well‐known reference energetic salts: guanidinium azotetrazolate GZT (410 kJ mol^−1^), triaminoguanidinium azotetrazolate TAGZT (1062 kJ mol^−1^), and triaminoguanidinium nitrate TAGN (−48 kJ mol^−1^). Notably, the majority of newly prepared anhydrous salts exhibit significantly higher enthalpy of formation of both TAGN and GZT. For instance, compounds **ImAzTz·H**_**2**_ **O** (712.3 kJ mol^−1^), **DATrAzTz** (825.3 kJ mol^−1^), **MBATrAzTz·H**_**2**_ **O** (741.7 kJ mol^−1^), and **DAAmGTrAzTz** (739.9 kJ mol^−1^) exhibit substantially higher endothermic enthalpies than the GZT baseline. Furthermore, compound **ATrAzTz** (1054 kJ mol^−1^) demonstrates an exceptional enthalpy of formation, effectively matching the highly endothermic nature of the high energetic TAGZT. This confirms that combining the nitrogen‐rich azotetrazolate anion with appropriately tailored high‐nitrogen cations yields highly competitive energetic materials.

From an industrial processing perspective, when substances are mixed to form pyrotechnic compositions, particle morphology and bulk density are fundamental for mixing and homogenization. Except for the imidazole salt **ImAzTz·H**_**2**_ **O**, which forms needles up to several hundred micrometers long, all other substances consist of heterogeneous, rounded agglomerates of smaller crystals, ranging in size from a few micrometers to a few hundred micrometers. The rounded agglomerate shape is desirable, as it results in good flowability and a higher bulk density than the reference substances GZT, TAGZT, and TAGN, which are composed of separate needle‐shaped particles (Figures S37–S39). All studied compounds have a higher or the same bulk density as the reference GZT, TAGZT, and TAGN; materials **DAAmGTrAzTz** and **GBAAmHAzTz·2H**_**2**_ **O** have an excellent bulk density exceeding 0.5 g cm^−3^, with compound **GBAAmHAzTz·2H**_**2**_ **O** reaching as high as 0.77 g cm^−3^ (Table 2).

**TABLE 2:** Bulk densities and hygroscopicity of all compounds studied and reference substances GZT, TAGZT, and TAGN.

Compound	Bulk density,g cm^−3^	Weight gain after 20 days, %	
		RH 64%	RH 84%
ImAzTz·H_2_O	0.320	0.188	0.526
ATrAzTz	0.338	0.121	0.216
DATrAzTz	0.334	0.187	0.071
MBATrAzTz·H_2_O	0.472	0.181	1.308
DAAmGTrAzTz	0.545	0.141	3.947
HMTAzTz·H_2_O	0.383	4.603	10.552
ODHAzTz·3H_2_O	0.235	0.021	1.635
DMBGAzTz·H_2_O	0.490	0.078	0.074
GBAmHAzTz·2H_2_O	0.363	0.237	2.064
GBAAmHAzTz·2H_2_O	0.774	0.537	3.014
GZT	0.245	0.113	0.206
TAGZT	0.270	0.001	0.003
TAGN	0.236	0.121	0.083

The hygroscopicity of the studied substances was determined at 64% and 84% relative humidity, as reported in the literature [13]. Except for **GBAmHAzTz·2H**_**2**_ **O** and **GBAAmHAzTz·2H**_**2**_ **O**, all compounds have low hygroscopicity at 64% air humidity (Table 2). Based on the weight gain measured at 84% relative humidity, the compounds were classified into three groups. Substances with a weight gain of up to 0.20% (**DATrAzTz**, **DMBGAzTz·H**_**2**_ **O**, TAGZT, and TAGN) were assigned to the low‐hygroscopicity group. Materials showing values between 0.21% and 0.55% (**ImAzTz·H**_**2**_ **O**, **ATrAzTz**, **GBAmHAzTz·2H**_**2**_ **O**, and GZT) were categorized as moderately hygroscopic. Compounds exceeding 0.55% (**MBATrAzTz·H**_**2**_ **O**, **DAAmGTrAzTz**, **HMTAzTz·H**_**2**_ **O**, **ODHAzTz·3H**_**2**_ **O**, **GBAmHAzTz·2H**_**2**_ **O**, and **GBAAmHAzTz·2H**_**2**_ **O**) were placed in the higher‐hygroscopicity group. The classification thresholds of 0.20% and 0.55% were chosen to reflect the natural distribution of the measured data. Overall, the hygroscopicity values observed at 64% and 84% RH indicate that most materials remain suitable for processing under standard laboratory and industrial conditions.

### 3.4 Sensitivity to Mechanical Stimuli

In terms of safety characteristics related to processing and handling, sensitivity to impact and friction is essential. The sensitivities of our substances were compared with the sensitivity of GZT, TAGZT, and TAGN, often mentioned as promising gas‐generating agents and commonly industrially produced explosives, pentaerythritol tetranitrate (PETN), and 1,3,5‐trinitro‐1,3,5‐triazinane (RDX).

**FIGURE 14:**
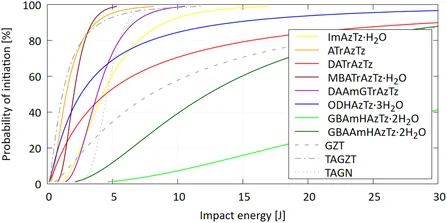
Impact sensitivity curves for *N*‐rich salts of 5,5′‐azotetrazole compared to the sensitivity of guanidinium 5,5′‐azotetrazolate (GZT), triaminoguanidinium 5,5′‐azotetrazolate (TAGZT), and triaminoguanidine nitrate (TAGN).

The impact sensitivity ranged widely, from substances that are completely insensitive to substances with the sensitivity on the level of primary explosives (Figure 14, Tables S20 and S22). The 5,5′‐azotetrazole salts with the hexamine **HMTAzTz·H**_**2**_ **O** and dimethylbiguanide **DMBGAzTz·H**_**2**_ **O** are insensitive to impact, not providing any reaction even under an impact energy of 50 J. The 5,5′‐azotetrazole salts with glyoxalbisamidinohydrazones, **GBAmHAzTz·2H**_**2**_ **O** and **GBAAmHAzTz·2H**_**2**_ **O** have very low impact sensitivity. These salts are even less sensitive than the gas‐generating agents GZT and TAGN. The compounds **ImAzTz·H**_**2**_ **O**, **DATrAzTz**, and **DAAmGTrAzTz** have sensitivity at the level of secondary explosives, within the sensitivity between the sensitivity of pentaerythritol tetranitrate (PETN) and 1,3,5‐trinitro‐1,3,5‐triazinane (RDX). The sensitivity of **ATrAzTz**, **MBATrAzTz·H**_**2**_ **O**, and **ODHAzTz·3H**_**2**_ **O** is at the level of primary explosives such as mercury fulminate, lead styphnate [46], or tetrazene [47]. However, TAGZT is even more sensitive than these three compounds.

Contrary to expectation, the generally known positive impact of crystalline water content on reducing sensitivity to mechanical stimuli (e.g., for the sodium salt of 5,5′‐azotetrazole, recently published [37, 48]) was not evident in the case of *N*‐rich salts of 5,5′‐azotetrazole. This is most evident in compound **ODHAzTz·3H**_**2**_ **O**, containing three molecules of water of crystallization, and showing higher sensitivity than compounds **ImAzTz·H**_**2**_ **O**, **DATrAzTz, DAAmGTrAzTz**, **HMTAzTz·H**_**2**_ **O**, and **DMBGAzTz·H**_**2**_ **O** with zero or only one molecule of water of crystallization.

An attempt to explain a relationship between chemical structure and impact sensitivity in nitrogen‐rich compounds was reported by Forquet et al. [49]. Their study focused on a series of 2,2‐dimethyltriazanium salts, including the 5,5′‐azotetrazolate salt. The authors explained the differences in impact sensitivity among the investigated salts in terms of hydrogen‐bond stabilization, which plays a significant role in crystal packing. In the present work, crystal structures were obtained for five compounds. However, three of them, namely **HMTAzTz**, **GBAmHAzTz**, and **DMBGAzTz**, crystallized as hydrates that differed in number of water of crystallization from the corresponding bulk materials, whereas a fourth compound, **DAAmGTrAzTz**, underwent cyclization during single‐crystal growth. Consequently, **DATrTzAz** was the only compound found to contain the same number of water molecules of crystallization in both the single‐crystal and bulk samples. Owing to the absence of crystal water, **DATrTzAz** adopts a layered structure with a planar azotetrazolate anion and 3,5‐diamino‐4*H*−1,2,4‐triazol‐1‐ium cation. This architecture is stabilized by extensive H‐bonding network and **DATrTzAz** falls within the category of moderately sensitive compounds (*E*_50_ = 3.83 J). For **HMTAzTz**, **GBAmHAzTz**, and **DMBGAzTz**, the single crystals contained approximately twice as much crystal water as the corresponding bulk materials (2 and 4 water molecules versus 1 and 2, respectively). Therefore, a reliable evaluation of the influence of crystal structure on impact sensitivity cannot be made for these compounds. Nevertheless, it is noteworthy that most other nitrogen‐rich 5,5′‐azotetrazole salts reported in the literature, for which single‐crystal X‐ray diffraction (scXRD) data are also available, have been described as impact‐insensitive despite being predominantly anhydrous. Examples include salts of hydrazine and its derivatives [43, 50], guanylurea a biuret [51, 52], 3,4,5‐triamino‐1,2,4‐triazole and its methyl derivate [53, 54, 55, 56], as well as 5‐aminotetrazole and its amino‐ and methyl‐substituted derivates [57, 58]. In contrast, impact‐sensitive 5,5′‐azotetrazole salts have been reported for a range of substituted triazoles, including 1‐amino‐3‐methyl‐1,2,3‐triazole (28.2 J) [59], 5,5′‐(ethene‐1,2‐diyl)bis(3,4‐diamino‐1,2,4‐triazole) (10 J) [60], and 1‐guanyl‐1,2,4‐triazole (10 J) [61], as well as amino derivatives of guanidinium azotetrazolate (3 to >40 J) [34] and dimethyltriazania salts [49]. In all these cases, the compounds are also anhydrous. The impact sensitivities of most of these salts fall within the moderately sensitive range, typically around 10 J. A markedly different trend was observed for the salts prepared in the present study. Six compounds were classified as sensitive or very sensitive, with impact sensitivities ranging from 1.44 to 4.5 J. Half of these compounds are anhydrous, whereas the other half crystallize as hydrates. Taken together, these observations demonstrate that nitrogen‐rich 5,5′‐azotetrazole salts can exhibit a remarkably wide range of impact sensitivities. At present, the available data do not allow a clear and unambiguous correlation to be established between chemical structure, crystal water content, and impact sensitivity. These findings suggest that the factors governing the mechanical sensitivity of nitrogen‐rich 5,5′‐azotetrazole salts are more complex and warrant further systematic investigation, particularly with respect to the role of crystal packing and intermolecular interactions.

From a processing standpoint, sensitivity to friction is most significant. None of the measured compounds was initiated by even the highest friction force of 360 N and are therefore considered insensitive to friction. However, sensitivity to friction was observed in two reference substances, TAGN and TAGZT, with 50% initiation probabilities of 144 N for TAGN and 171 N for TAGZT, which are slightly lower than those of RDX [62].

### 3.5 Applications in Gas Generator Compositions

Azotrazolates are efficient sources of gaseous products with high gas yield, particularly nitrogen, which is essential for the operation of gas generators. To ensure rapid and reliable release of gaseous products with a favorable composition at the required rate, azotetrazolate must be mixed with a suitable oxidizer. Another key component of the pyrotechnic composition is a binder, which ensures the required mechanical strength of the final product in the form of tablets or granules.

In the first step, key parameters of the individual substances were calculated using the REAL thermodynamic software [63]. The initial screening of pure compounds reveals their inherent energetic potential, independent of a propellant matrix. Performance was evaluated primarily through the force (*f*), also known as impetus or thermodynamic force, one of the fundamental energetic characteristics of a propellant. It is commonly expressed as *f *= *n*_g_ *R T*, where *n*_g_ is the molar number of gaseous products generated per unit mass of the compound, *R* is the universal gas constant, and *T* is the adiabatic flame temperature. Force represents the “strength” or work capability of the propellant. As shown in Table 3, there is a clear dependency between force and the structural and thermochemical characteristics of the compounds. While high nitrogen content promotes a large gas volume *n*_g_, the enthalpy of formation dictates the flame temperature *T*, acting as a key driving factor. Thus, both factors are relevant for practical application. For example, although **DAAmGTrAzTz** possesses slightly more nitrogen than **ATrAzTz** (76.2% vs. 75.4%), **ATrAzTz** achieves a significantly higher force (841.7 vs. 698.6 kJ kg^−1^). High force of **ATrAzTz** is directly attributable to the exceptionally high positive enthalpy of formation of **ATrAzTz** (+3154 kJ kg^−1^), which raises the combustion temperature by more than 400 °C above that of **DAAmGTrAzTz**. Overall, while TAGZT and TAGN achieve the highest absolute values, several synthesized salts significantly outperform the GZT baseline, confirming their strong potential for practical gas‐generating formulations.

**TABLE 3:** Powder force of investigated compounds, GZT, TAGZT, and TAGN, along with other related important parameters.

Compound	OB, %^a^	*N*, %^b^	Δ_f_ *H* ^°^, kJ kg^−^^1^ c	*n*_g_, mol kg^−^^1^ d	*T* °,C^e^	*f*, kJ kg^−1^ f
ImAzTz·H_2_O	−104.9	61.2	2224	42.31	1815	734.5
ATrAzTz	−81.4	75.4	3154	41.40	2172	841.7
DATrAzTz	−79.1	76.9	2266	42.70	1744	716.1
MBATrAzTz·H_2_O	−83.5	69.2	2036	42.74	1784	731.1
DAAmGTrAzTz	−80.2	76.2	2246	41.78	1738	698.6
HMTAzTz·H_2_O	−141.2	54.3	297.6	40.10	1015	429.4
ODHAzTz·3H_2_O	−52.6	55.3	−1613	46.48	1126	540.7
DMBGAzTz·H_2_O	−115.7	63.3	−10.76	42.01	939	423.5
GBAmHAzTz·2H_2_O	−77.4	67.7	1106	47.76	1486	698.5
GBAAmHAzTz·2H_2_O	−75.6	69.6	1399	49.49	1590	766.6
GZT	−78.8	78.8	1442	45.28	1335	605.4
TAGZT	−72.7	82.3	2850	52.04	1957	965.0
TAGN	−33.5	58.7	−288	53.84	2306	1154

a OB based on CO_2_ formation.

b Nitrogen content in wt percents.

c Experimentally determined enthalpy of formation.

d Molar amount of gaseous products per unit mass of the compound.

e Adiabatic flame temperature.

f Force (*f *= *n*_g_ *R T*).

In the second step, it is necessary to calculate the parameters of the final gas generator composition, which includes both the oxidizer and the binder. We decided to use ammonium perchlorate/strontium nitrate (wt. ratio 1:1) as an oxidizer and sodium carboxymethyl cellulose (NaCMC) as a binder based on a well‐established green propellant matrix employing GZT [64]. To ensure a fair comparison and minimize the formation of toxic and under‐oxidized products, all formulations were balanced to a slightly positive OB of +0.5%. This specific value was chosen to match exactly the OB of the cited reference formulation containing GZT (the exact compositions of all mixtures are detailed and shown in Table S4).

The reference composition containing GZT yielded a force of 765.1 kJ kg^−1^. Most of the newly synthesized salts outperformed this baseline (Table 4). The highest performance among the novel series was achieved by formulations containing compounds **ATrAzTz** and **GBAAmHAzTz·2H**_**2**_ **O**, yielding force values of 793.5 and 792.3 kJ kg^−1^, respectively. The driving factors behind their superior performance perfectly illustrate the dual dependence of *f* on *T* and *n*_g_. The performance of the mixture containing the compound **ATrAzTz** is thermally driven, achieving the highest combustion temperature (2990 °C) in the new series, owing to the exceptional positive enthalpy of formation of the neat salt. In contrast, the formulation with compound **GBAAmHAzTz·2H**_**2**_ **O** relies on a high gas‐generating capacity, providing the highest molar gas yield (30.23 mol kg^−1^).

**TABLE 4:** The calculated parameters for gas generator formulations azotetrazolate/NH_4_ClO_4_/Sr(NO_3_)_2_/NaCMC are balanced to +0.5% OB (exact compositions of all mixtures are detailed and shown in Table S4).

Azotetrazolate in NH_4_ClO_4_/Sr(NO_3_)_2_/NaCMC	Content of azotatrazolate, wt%^a^	*Q,*kJ kg^−^^1^ b	*n*_g,_mol kg^−^^1^ c	*T,*°C^d^	*f,*kJ kg^−^^1^ e
ImAzTz·H_2_O	20.4	4399	28.57	2958	767.7
ATrAzTz	24.6	4388	29.25	2990	793.5
DATrAzTz	25.0	4177	29.43	2890	773.8
MBATrAzTz·H_2_O	24.0	4268	29.09	2921	772.4
DAAmGTrAzTz	24.8	4168	29.26	2890	769.2
HMTAzTz·H_2_O	16.2	4188	28.51	2819	733.1
ODHAzTz·3H_2_O	32.4	3839	30.70	2663	749.5
DMBGAzTz·H_2_O	19.0	4054	29.21	2757	735.9
GBAmHAzTz·2H_2_O	25.4	4202	29.92	2858	778.8
GBAAmHAzTz·2H_2_O	25.8	4259	30.23	2880	792.3
GZT	25.0	4023	30.03	2792	765.1
TAGZT	26.4	4401	31.14	2947	833.6
TAGN	41.2	4470	34.15	2889	897.7

a Mass fraction of the investigated compound in the calculated mixture (for OB = +0.5%).

b Calculated heat of combustion.

c Molar amount of gaseous products per unit mass of the mixture.

d Adiabatic flame temperature.

e Powder force (*f *= *n*_g_ *R T*).

The OB of the investigated compounds (Table 3) also plays a critical role in the final performance of the mixture. Compounds with a more favorable OB, such as the reference TAGN (−33.5%), can be incorporated at significantly higher mass fractions (41.2 wt%) to reach the target mixture OB (+0.5% in our case). This maximizes the compound's gas‐generating contribution, leading to the highest absolute *f* (897.7 kJ kg^−1^, Table 4). The reference TAGZT demonstrated the second‐highest overall performance (833.6 kJ kg^−1^) by combining a high nitrogen content with a high enthalpy of formation. On the other hand, compounds **HMTAzTz·H**_**2**_ **O** and **DMBGAzTz·H**_**2**_ **O** suffer from highly negative OB values. This severely limits their mass content in the formulation, which, combined with their lower enthalpies of formation, inevitably yields the lowest *f* values. Overall, the data demonstrate that achieving optimal gas generator performance requires a delicate balance among the compound's nitrogen content, heat of formation, and OB, with compounds **ATrAzTz** and **GBAAmHAzTz·2H**_**2**_ **O** representing the optimal candidates from the presented series.

Based on its favorable physicochemical and ballistic properties, **ATrAzTz** was identified as a highly promising candidate for gas‐generating compositions. Therefore, this compound was selected to demonstrate that the combustion of a pyrotechnic composition containing azotetrazolate, an oxidizer, and a binder produces nontoxic gaseous products. As an oxidizer, a mixture of NH_4_ClO_4_ and Sr(NO_3_)_2_ was employed, where ammonium perchlorate provides high performance due to the formation of a large amount of gaseous products and its exothermic decomposition, while strontium nitrate primarily acts as a scavenger for chlorine and hydrogen chloride. Thermodynamic equilibrium modeling confirmed that adjusting the OB to +0.5% in formulations prevents the formation of hazardous under‐oxidized gases (Table 5). The predicted exhaust is essentially free of CO, HCN, NH_3_, and other toxic gases. Furthermore, the NH_4_ClO_4_/Sr(NO_3_)_2_ matrix provides a significant environmental advantage through chlorine scavenging. In a theoretical binary mixture of compound **ATrAzTz** with NH_4_ClO_4_ alone, the combustion produces a highly toxic and corrosive exhaust containing approximately 20.8 wt% of HCl and 0.6 wt% of Cl_2_. However, in the investigated mixtures containing Sr(NO_3_)_2_, the strontium cations effectively trap the released chlorine species, forming SrCl_2_. This mechanism significantly reduces gaseous HCl to a low level (1.5 ppm, 1.5 10^−^^4^ wt%). The same HCl content was also predicted for the composition GZT/NH_4_ClO_4_/Sr(NO_3_)_2_/NaCMC which was previously investigated experimentally [64], revealing that the HCl content in the real combustion products reached 4.5 ppm. Therefore, a similar reduction in HCl content in the real combustion products can be expected for other azotetrazolates. Consequently, the investigated compounds represent highly promising alternatives and energetic components for advanced, high‐nitrogen, environmentally friendly gas‐generating formulations. Moreover, as a final validation step, the calculated values must be verified through analysis of the final combustion product to ensure compliance with relevant standards and regulatory limits such as the U.S. CAR requirements [65].

**TABLE 5:** Calculated combustion products for ATrAzTz and reference GZT compositions.

Compound	Propellant composition, wt%		
NH_4_ClO_4_	35.0	35.3	71.7
Sr(NO_3_)_2_	35.0	35.3	—
GZT	25.0	—	—
ATrAzTz	—	24.6	23.3
NaCMC	5.0	5.0	5.0

Combustion product	Combustion product concentration, wt%		
N_2_ (g)	28.45	27.21	26.12
H_2_O (g)	22.29	19.45	25.56
CO_2_ (g)	21.67	25.48	25.67
O_2_ (g)	0.3120	0.3389	—
HCl (g)	1.5 ppm	1.5 ppm	19.24
Cl_2_ (g)	0.33 ppm	0.40 ppm	2.197
SrCl_2_ (s)	21.98	22.18	—
SrCO_3_ (s)	3.622	3.58	—
NaCl (s)	1.121	1.207	1.201
Sr(NO_3_)_2_	0.4711	0.5638	—

## 4 Conclusion

A series of ten nitrogen‐rich 5,5′‐azotetrazole salts was successfully synthesized and evaluated as promising gas‐generating materials. A significant advancement in their preparation is the use of chlorine in the synthesis of the starting sodium 5,5′‐azotetrazolate. This scalable approach replaces the traditional permanganate method, eliminating poorly filterable by‐products and providing a high‐yield, easily isolable product.

The study of physicochemical and energetic properties focused on parameters relevant to practical applications. In general, the compounds exhibit high heats of formation and favorable bulk densities. Diazole and triazole derivatives are mostly low‐hygroscopic, whereas the remaining compounds are more hygroscopic. None of the studied compounds is sensitive to friction, while their impact sensitivity varies widely, ranging from low‐sensitivity materials to those with sensitivities comparable to those of primary explosives. Despite the strict requirements for the use of compounds in gas generators, several promising candidates have been identified, particularly azotetrazole salts of diazole and triazole derivatives.

Calculation of the tetrazolate/oxidizer/binder system in pyrotechnic compositions designed for gas‐generating systems confirms high energetic parameters. Crucially, thermodynamic calculations confirmed that utilizing an optimized oxidizer matrix produces nontoxic gases; owing to strontium nitrate, it effectively eliminates hydrogen chloride and chlorine.

Ultimately, the present study highlights the significant potential of tailored 5,5′‐azotetrazole derivatives with favorable crystal morphology and high bulk density, as easily accessible, high‐performance, and environmentally friendly propellants.

## Funding

This study was supported by Ministry of Education, Youth and Sports of the Czech Republic (projects SGS_2026_003 and LM2023037).

## Conflicts of Interest

The authors declare no conflicts of interest.

## Supporting information

Supplementary Material

## Data Availability

The data that support the findings of this study are available in the supplementary material of this article.

## References

[1] V. E. Melnikov , Sovremennaya Pirotekhnika, (Nauka, 2014), 271–277.

[2] J. A. Conkling and C. Mocella , Chemistry of Pyrotechnics, (CRC Press, 2019), 111–113.

[3] A. H. Parrot and H. Round , Air‐Cushion, US1331359, (1920).

[4] T. T. Griffiths , U. Krone , and R. Lancaster , “Pyrotechnics,” in Ullmann's Encyclopedia of Industrial Chemistry, (Wiley‐VCH, 2018), 1–21.

[5] J. W. Betrick , Safety Cushion Assembly for Automotive Vehicles, US 2649311, (1953).

[6] W. Linderer , Einrichtung Zum Schulze von in Fahrzeugen Befindlichen Personen gegen Verletzungen Bei Zusammenstößen, DE 896312, (1953).

[7] B. Halford , “What Chemicals Make Airbags Inflate, and How Have They Changed Over Time?,” Chemical & Engineering News 100 (2022): 1.

[8] E. A. Betterton , “Environmental Fate of Sodium Azide Derived From Automobile Airbags,” Critical Reviews in Environmental Science and Technology 33 (2010): 423–458.

[9] R. Meyer , J. Köhler , and A. Homburg , Explosives, 7^th^ ed. (Wiley‐VCH Verlag GmbH, 2015), 5.

[10] B. Gierczyk , M. Zalas , and T. Otłowski , “High‐Energetic Salts and Metal Complexes: Comprehensive Overview with a Focus on Use in Homemade Explosives (HME),” Molecules 29 (2024): 5588. 39683747 10.3390/molecules29235588 PMC11643457

[11] K. Hara , T. Yoshida , T. Misawa , et al., “Concept and Performance of a Non‐Azide Propellant for Automotive Airbag Inflators,” Propellants, Explosives, Pyrotechnics 23 (1998): 28–33.

[12] G. Jeyabalaganesh , S. P. Sivapirakasam , K. R. Balasubramanian , and S. L. Aravind , “Evaluation on Substitution of Conventional Azide Based Fuel Materials with an Alternate One in an Airbag System – A Review,” Materials Today: Proceedings 46 (2021): 9544–9549.

[13] U. Reimann and S. Zeuner , “Guanidinium Nitrate ‐ A Particularly Suitable Fuel in Propellants,” International Pyrotechnic Automotive Safety Symposium (IPASS), (Association Française de Pyrotechnie and Fraunhofer Institute for Chemical Technology, 2005).

[14] M. A. Hiskey , N. Goldman , and J. R. Stine , “High‐Nitrogen Energetic Materials Derived from Azotetrazolate,” Journal of Energetic Materials 16 (1998): 119–127.

[15] W. S. McEwan and M. W. Rigg , “The Heats of Combustion of Compounds Containing the Tetrazole Ring,” Journal of the American Chemical Society 73 (1951): 4725–4727.

[16] V. A. Ostrovskii , E. A. Popova , and R. E. Trifonov , “Developments in Tetrazole Chemistry (2009‐16),” in Advances in Heterocyclic Chemistry, eds. E. F. V. Scriven, and C. A. Ramsden (Academic Press, 2017). 1–62.

[17] R. E. Trifonov , I. Alkorta , V. A. Ostrovskii , and J. Elguero , “A Theoretical Study of the Tautomerism and Ionization of 5‐Substituted NH‐Tetrazoles,” Journal of Molecular Structure: THEOCHEM 668 (2004): 123–132.

[18] M. S. Akdemi , “Tetrazole‐Containing Polymers: Synthesis and Characterization,” (dissertation, Karlsruher Instituts für Technologie, 2023).

[19] Y. Liu , M. Lv , G. Zhang , Z. Dong , and Z. Ye , “Combination of Energetic Tetrazole and Triazole: Promising Materials with Exceptional Stability and Low Mechanical Sensitivity as Propellants and Gas Generators,” ACS Applied Materials & Interfaces 15 (2023): 15311–15320. 36926825 10.1021/acsami.2c20871

[20] M. Thoenen , A. Merino , J. E. Zuckerman , M. Zeller , E. F. C. Byrd , and D. G. Piercey , “High‐Nitrogen Materials Derived from 5‐Nitromethyl‐1*H*‐Tetrazole,” Inorganic Chemistry 64 (2025): 16454–16460. 40749140 10.1021/acs.inorgchem.5c02225

[21] Y. Wang , X. Li , Z. Han , and T. Zhang , “Monocrystalline of a Fused Ring Nitrogen‐Rich Compound and Applied in Gas Generator,” Journal of Energetic Materials 40 (2022): 421–428.

[22] S. R. Ganta and G. K. Williams , Gas Generating Compositions, US 9045380, (TK Holdings Inc., 2015).

[23] R. Matyáš and J. Pachman , Primary Explosives, (Springer, 2013), 203–207.

[24] T. M. Klapötke , Chemistry of High‐Energy Materials, (De Gruyter, 2022), 322–328.

[25] W. H. Gilligan and M. J. Kamlet , Synthesis of Mercuric 5‐Nitrotetrazole, Report No. NSWC/WOL TR. 76‐146, (Navel Surface Weapons Center, 1976).

[26] W. H. Gilligan and M. J. Kamlet , Method of Preparing the Acid Copper Salt of 5‐Nitrotetrazole, US 4093623, (US Navy, 1978).

[27] M. A. Ilyushin , A. V. Smirnov , V. N. Andreev , I. V. Tselinskii , I. V. Shugalei , and O. M. Nesterova , “Environmental Issues in the Synthesis and Purification of 5‐Nitrotetrazole Sodium Salt,” Russian Journal of General Chemistry 85 (2015): 2878–2885.

[28] J. Kralj , E. Murphy , K. Jensen , M. Williams , and R. Renz , “Preparation of Sodium Nitrotetrazolate Using Microreactor Technology,” in 41st AIAA/ASME/SAE/ASEE Joint Propulsion Conference & Exhibit, (American Institute of Aeronautics and Astronautics, 2005), 3516.

[29] J. C. Bottaro , M. A. Petrie , J. G. Bragg , J. W. Fronabarger , and D. H. Williams , Facile Method for Preparation of 5‐Nitrotetrazoles Using a Batch System, US 9598380, (SRI International; Pacific Scientific Energetic Materials, 2017).

[30] J. W. Fronabarger and M. D. Williams , Facile Method for Preparation of 5‐Nitrotetrazles Using Flow System, WO 2014/116654, (Pacific Scientific Energetic Materials, 2014).

[31] N. Mehta , K. Oyler , G. Cheng , J. Salan , and S. Lenahan , Synthesis of Copper(I) 5‐Nitrotetrazolate, US 9440934, (Secretary of the Army, 2016).

[32] R. H. Muir , J. Bragg , A. Pearsall , et al., “Development of a Safe Continuous Process to Sodium Nitrotetrazolate via Solid Phase “Catch and Release”,” Organic Process Research & Development 25 (2021): 1882–1888.

[33] D. D. Ford , S. Lenahan , M. Jörgensen , et al., “Development of a Lean Process to the Lead‐Free Primary Explosive DBX‐1,” Organic Process Research & Development 19 (2015): 673–680.

[34] A. Hammerl , M. A. Hiskey , G. Holl , et al., “Azidoformamidinium and Guanidinium 5,5′‐Azotetrazolate Salts,” Chemistry of Materials 17 (2005): 3784–3793.

[35] J. Thiele , “Ueber Nitro‐ Und Aminoguanidin,” Justus Liebigs Annalen der Chemie 270 (1892): 1–63.

[36] A. Hahma , K. Ereo , J. Stierstorfer , and T. M. Klapötke , 5,5′‐Azotetrazolat‐Sprengstoff, DE. 102010025104, (Diehl BGT Defence GmbH, 2011).

[37] M. Labaj , Z. Jalový , R. Matyáš , J. Nesveda , J. Mikuláštík , and A. Votýpka , “One‐Pot Synthesis of guanidinium 5,5′‐Azotetrazolate Avoiding Isolation of Hazardous Sodium 5,5′‐Azotetrazolate,” Organic Process Research & Development 28 (2024): 4091–4098.

[38] Y. H. Wang , Z. M. Du , C. L. He , X. M. Cong , H. S. Wang , and C. Y. Wang , “Synthesis and Characterization of GZT,” Hanneng Cailiao 16 (2008): 581–584.

[39] N. Kumbhakarna , S. T. Thynell , A. Chowdhury , and P. Lin , “Analysis of RDX‐TAGzT Pseudo‐Propellant Combustion with Detailed Chemical Kinetics,” Combustion Theory and Modelling 15 (2011): 933–956.

[40] K. M. Bucerius , F.‐W. Wasmann , and K. Menke , Stable, Nitrogen‐Rich Composition, US 5198046, (Fraunhofer‐Gesellschaft Zur Forderung Der Angewandten Forschund e.V., 1993).

[41] W. Ma , J. Li , C. He , and S. Pang , “Synthesis of Two Series of Isomeric Triazolo‐Triazine Fused Energetic Salts: High Thermal Stability, Insensitivity, and High Performances,” Crystal Growth & Design 23 (2023): 3463–3470.

[42] Z. Zeng , R. Wang , B. Twamley , D. A. Parrish , and J. M. Shreeve , “Polyamino‐Substituted Guanyl‐Triazole Dinitramide Salts with Extensive Hydrogen Bonding: Synthesis and Properties as New Energetic Materials,” Chemistry of Materials 20 (2008): 6176–6182.

[43] A. Hammerl , G. Holl , M. Kaiser , et al., “New Hydrazinium Salts of 5,5′‐Azotetrazolate,” Zeitschrift für Naturforschung B 56 (2001): 857–870.

[44] A. J. Bracuti , The Crystal Structure Determination of Triaminoguanidinium Nitrate: A Burning Rate Modifier for Nitramine Propellants, Report No. ARLCD‐TR‐78050, (Large Caliber Weapon Systems Laboratory, 1979).

[45] K.‐Y. Lee and M. M. Stinecipher , “Synthesis and Initial Characterization of Amine Salts of 3‐Nitro‐1,2,4‐Triazol‐5‐One,” Propellants, Explosives, Pyrotechnics 14 (1989): 241–244.

[46] T. Musil , R. Matyáš , R. Vala , A. Růžička , and M. Vlček , “Silver Salt of 4,6‐Diazido‐*N*‐Nitro‐1,3,5‐Triazine‐2‐Amine – Characterization of This Primary Explosive,” Propellants, Explosives, Pyrotechnics 39 (2014): 251–259.

[47] J. Ryšavý , R. Matyáš , Z. Jalový , et al., “Tetrazene–Characterization of Its Polymorphs,” Molecules 26 (2021): 7106. 34885685 10.3390/molecules26237106 PMC8658986

[48] J. Mikuláštík , V. A. M. Labaj , J. Nesveda , et al., “Characterizing the Decomposition and Hydrate Stability of Sodium 5,5′‐Azotetrazolate,” in 27th Seminar on New Trends in Research of Energetic Materials, (University of Pardubice, 2025): 431–438.

[49] V. Forquet , C. M. Sabaté , G. Jacob , Y. Guelou , H. Delalu , and C. Darwich , “Energetic 2,2‐Dimethyltriazanium Salts: A New Family of Nitrogen‐Rich Hydrazine Derivatives,” Chemistry An Asian Journal 10 (2015): 1668–1675. 25975814 10.1002/asia.201500397

[50] A. Hammerl , T. M. Klapötke , H. Nöth , et al., “[N_2_H_5_]^+^2[N_4_C−NN−CN_4_]^2‐^: A New High‐Nitrogen High‐Energetic Material,” Inorganic Chemistry 40 (2001): 3570–3575. 11421707 10.1021/ic010063y

[51] T. M. Klapötke and M. C. Sabaté , “Low Energy Monopropellants Based on the Guanylurea Cation,” Zeitschrift für Anorganische Und Allgemeine Chemie 636 (2010): 163–175.

[52] T. M. Klapötke and C. M. Sabaté , “Energetic Salts with Guanylurea Cation,” in 11th Seminar on New Trends in Research of Energetic Materials, (University of Pardubice, 2008), 672–677.

[53] C. Darwich , T. M. Klapötke , and C. M. Sabaté , “Structural and Preliminary Explosive Property Characterizations of New 3,4,5‐Triamino‐1,2,4‐Triazolium (Guanazinium) Salts,” Propellants, Explosives, Pyrotechnics 33 (2008): 336–346.

[54] C. Darwich , T. M. Klapötke , and C. M. Sabaté , “1,2,4‐Triazolium‐Cation‐Based Energetic Salts,” Chemistry – A European Journal 14 (2008): 5756–5771. 18523937 10.1002/chem.200800340

[55] T. M. Klapötke and C. M. Sabaté , “Bistetrazoles: Nitrogen‐Rich, High‐Performing, Insensitive Energetic Compounds,” Chemistry of Materials 20 (2008): 3629–3637.

[56] T. M. Klapötke and C. M. Sabaté , “Bridged‐Bistetrazole Derivates,” in 11th Seminar on New Trends in Research of Energetic Materials, (University of Pardubice, 2008), 234–245.

[57] T. M. Klapötke and C. M. Sabaté , “Nitrogen‐Rich Tetrazolium Azotetrazolate Salts: A New Family of Insensitive Energetic Materials,” Chemistry of Materials 20 (2008): 1750–1763.

[58] Z. Zhao , Z. Du , Z. Han , Y. Zhang , and C. He , “Nitrogen‐Rich Energetic Salts: Both Cations and Anions Contain Tetrazole Rings,” Journal of Energetic Materials 34 (2016): 183–196.

[59] Q.‐H. Lin , Y.‐C. Li , Y.‐Y. Li , et al., “Energetic Salts Based on 1‐Amino‐1,2,3‐Triazole and 3‐Methyl‐1‐Amino‐1,2,3‐Triazole,” Journal of Materials Chemistry 22 (2012): 666–674.

[60] Q. Ma , Y. Chen , L. Liao , H. Lu , G. Fan , and J. Huang , “Energetic π‐Conjugated Vinyl Bridged Triazoles: A Thermally Stable and Insensitive Heterocyclic Cation,” Dalton Transactions 46 (2017): 7467–7479. 28561100 10.1039/c7dt01261f

[61] R. Deblitz , C. G. Hrib , and F. T. Edelmann , “Structures and Energetic Properties of Two New Salts Comprising the 5,5′‐Azotetrazolate Dianion,” Crystals 5 (2015): 405–417.

[62] M. Künzel , R. Matyáš , O. Vodochodský , and J. Pachman , “Explosive Properties of Melt Cast Erythritol Tetranitrate (ETN),” Central European Journal of Energetic Materials 14 (2017): 418–429.

[63] G. V. Belov , “Thermodynamic Analysis of Combustion Products at High Temperature and Pressure,” Propellants, Explosives, Pyrotechnics 23 (1998): 86–89.

[64] J. Zigmund , P. Kohlíček , and I. Varga , “Development of a Green Propellant,” SAFEX Newsletter 65 (2018): 18–25.

[65] USCAR inflator technical requirements and validation, SAE/USCAR‐24 , (SAE International, 2023).

