# Supplementary material for: Nitrogen‐Rich 5,5′‐Azotetrazolates: A Broad Spectrum of Technologically Accessible Gas‐Generating Pyrotechnic Materials
Source: ChemistryOpen. 2026 Aug 31;15(9):e70288. doi: 10.1002/open.70288 (PMC13527586; doi:10.1002/open.70288)
Supplement: Supplementary file 1 [file OPEN-15-e70288-s001.pdf]

Nitrogen-Rich 5,5'-Azotetrazoles – A Broad Spectrum of Technologically Accessible Gas-Generating Pyrotechnic Materials

Adam Votýpka,[a] Robert Matyáš,*[a] Jakub Mikuláštík,[a] Aleš Růžička,[b] Zdeňka Růžičková,[b] Jan Zigmund,[a] Miroslav Labaj,[a] Martin Adam,[c] Zdeněk Jalový[a]

[a] Institute of Energetic Materials
Faculty of Chemical Technology, University of Pardubice
Studentská 573, CZ-532 10, Pardubice, Czech Republic
E-mail: robert.matyas@upce.cz

[b] Department of General and Inorganic Chemistry
Faculty of Chemical Technology, University of Pardubice
Studentská 573, CZ-532 10, Pardubice, Czech Republic

[c] Department of Analytical Chemistry
Faculty of Chemical Technology, University of Pardubice
Studentská 573, CZ-532 10, Pardubice, Czech Republic

Table of Contents

General Methods

Fourier Transform Infrared Spectroscopy S3
Elemental Analysis S3
Karl-Fischer titration S3
Single-crystal X-ray diffraction S3
Differential thermal analysis S4
Scanning electron microscopy S4
Density S4
Bulk density S4
Hygroscopicity S4
Combustion calorimetry S4
Friction and impact sensitivity S4

Synthesis

Caution S6

Sodium 5,5-azotetrazolate pentahydrate S6

3-Amino-1,2,4-triazole 5,5'-azotetrazolate (ATrAzTz) S6

3,5-Diamino-1,2,4-triazole 5,5'-azotetrazolate (DATrAzTz) S7

5,5´-Methylenebis(3-amino-1*H*-1,2,4-triazol) 5,5'-azotetrazolate monohydrate (MBATrAzTz·H_2O) S7

3,5-Diamino-*N*-amidino-1-guanyl-1,2,4-triazole azotetrazolate (DAAmGTrAzTz) S7

Hexamethylenetetramine 5,5'-azotetrazolate monohydrate (HMTAzTz·H_2O) S8

Oxalyldihydrazide 5,5´-azotetrazolate trihydrate (ODHAzTz·3H_2O) S8

1,1'-Dimethylbiguanide 5,5'-azotetrazolate hydrate (DMBGAzTz·H_2O) S8

Glyoxal bis(amidinohydrazone) 5,5'-azotetrazolate dihydrate (GBAmHAzTz·2H_2O) S9

Glyoxal bis(aminoamidinohydrozone) 5,5'-azotetrazolate dihydrate (GBAAmHAzTz·2H_2O) S9

Guanidine 5,5'-azotetrazolate (GZT) S9

Triaminoguanidine 5,5'-azotetrazolate (TAGZT) S9

Triaminoguanidine nitrate (TAGN) S10

Preparation of crystals for X-ray analysis S10

Computational methods

Heat of formation calculation S11

Calculation for pyrotechnic S13

Experimental data

FTIR spectrum S16

Karl fisher titration data S23

DTA records S24

SEM data S31

Crystallographic data from single-crystal X-ray diffraction S36

Heat of combustion and heat of formation data S46

Hygroscopicity data S47

Sensitivity data S48

References S52

EXPERIMENTAL SECTION

1. General Methods

Fourier Transform Infrared Spectroscopy

Infrared spectra were collected using a Nicolet iS50 FT-IR spectrometer (Thermo Fisher, USA) with an iS50ATR diamond. Measurement parameters were the spectral regions of 4000−400 cm^{-1}, the resolution of 4 cm^{-1}, and 64 scans. Measured FTIR spectra are included in the Supplementary Information section (Fig. S1-S10).

Elemental analysis

Elemental analysis was carried out using a UNICUBE automatic elemental analyzer (Elementar, Germany) on 1−2 mg samples.

Karl Fisher titration

For the determination of the water content in prepared compounds, a KF 831 Coulometer (Metrohm, Herisau, Switzerland) was used. The sample amount (10 to 50 mg depending on the expected water content) was placed into the coulometric vessel containing the Aquastar™ CombiCoulomat fritless Karl Fischer reagent. All titrations were performed in triplicate. The results were expressed as % m/m

Single-crystal X-ray diffraction

Full-sets of diffraction data were collected at 150(2)K with a Bruker D8-Venture diffractometer equipped with a Cu (Cu/Kα radiation; λ =1.54178 Å) or Mo (Mo/Kα radiation; λ = 0.71073 Å) microfocus X-ray (IµS) source, Photon III CMOS detector and an Oxford Cryosystem cooling device was used for data collection. The frames were integrated with the Bruker SAINT software package using a narrow-frame algorithm. Data were corrected for absorption effects using the Multi-Scan method (SADABS) [1]. Obtained data were treated by XT-versions 2014/5, SHELXT 2018/2 [2] and SHELXL-2018/3 software [3] implemented in the APEX3 / APEX4 (Bruker AXS) system [4]. The hydrogen atoms were placed at calculated positions and refined using the "riding model". Some H atoms were localized on a difference Fourier map. Heavy atoms were refined anisotropically. Hydrogen atoms were mostly located on the difference Fourier map, however, for the final solution of the crystal structure, all hydrogen atoms were recalculated into ideal positions (riding model) according to the assigned temperature factors Hiso(H) = 1.2 Ueq for aryl groups and Hiso(H) = 1.5Ueq for aliphatic groups with C-H bond lengths = 0.96; 0.97; 0.98 and 0.93 Å for methyl, methylene, methine and hydrogen atoms of aromatic rings, respectively, and 0.86 or 0.82 Å for N-H or O-H bonds. Disordered parts of molecules were treated by standard methods, splitting azenyl group or a heterocycle (with minimum occupancy of major component of 80%) to two positions.

Crystallographic data for structural analysis of all compounds have been deposited with the Cambridge Crystallographic Data Centre, CCDC nos. 2551349-2551353. Copies of this information may be obtained free of charge from The Director, CCDC, 12 Union Road, Cambridge CB2 1EY, UK (fax: +44-1223-336033; e-mail: deposit@ccdc.cam.ac.uk or www: http://www.ccdc.cam.ac.uk).

Differential Thermal Analysis

Thermal analysis was performed using a DTA 550Ex differential thermal analyser (OZM Research, Czech Republic). The samples were tested in open glass microtest tubes in contact with air at a heating rate of 5 °C min^{-1}. The sample weights were 30 mg.

Scanning electron microscopy

Investigated compounds were visualized using a scanning electron microscope (SEM; Jeol JSM 5500 LV, JEOL, Akishima, Japan). The accelerating voltage of the primary electron beam was 10 kV using a secondary electron detector (SEI).

Density

Densities of solid materials were determined using a Helium pycnometer (Accu Pyc II 1340, Micromeritics, USA) at a temperature of 23.6°C.

Bulk density

The bulk density was measured in a small-scale custom block of 5.12 cm^3 in volume. Measurement was repeated seven times.

Hygroscopicity

For the determination of the hygroscopicity, defined samples were exposed to the two different humidities at a temperature of 25 °C. Every sample was weighed in a desiccator whose bottom was coated with saturated salt solution. A sodium nitrite solution generates 64 % and a potassium chloride solution generates 84 % relative humidity. The samples were stored in the desiccator for 20 days at each humidity and the resulting hygroscopicity was analyzed by means of the weight difference of the samples.

Combustion calorimetry (heat of combustion)

The heat of combustion Q_c was measured using the BCA 500L bomb combustion calorimeter (produced by OZM Research). Calibration was performed with benzoic acid (BA) to yield a temperature rise of approximately 0.5 K (0.25g of BA). Burning conditions were 3-3.1 MPa O_2, the ignition was done by cotton fiber connected to resistance wire bridge, the heat of combustion of the cotton fiber was subtracted from the result before further processing. Investigated compounds were measured compressed in a tablet weighing 0.5 g.

Friction and impact sensitivity

The sensitivity to impact was measured using a Kast fall hammer. The measurement apparatus and related supplies (guide rings BFH-SR and steel cylinders BFH-SC) were manufactured by OZM Research (Hrochův Týnec, Czech Republic). We used a 0.5, 1, 2 and 5 kg hammer for the measurement. A probit analysis was used to measure and evaluate the data. [5] Five energy levels were measured for 15 trials for each energy level.

The sensitivity to friction was determined using a BAM FSA-12 type FSA-12. The measurement apparatus and related supplies (porcelain plates BFST Pt 100 25 × 25 mm and porcelain BFST Pn 200 pegs) were manufactured by OZM Research (Hrochův Týnec, Czech

Republic). Probit analysis was used for measuring and evaluating [5]. Five energy levels were measured for 15 trials for each friction force level.

Friction and impact sensitivity of studies samples were compared with sensitivity of GZT, TAGZT and TAGN.

2. Synthesis

Caution!

Energetic tetrazole compounds are sensitive compounds; proper safety precautions should be taken when handling the materials. Safety equipment, such as goggles or a face shield, gloves, and equipment, should be properly grounded. Samples of 5,5′-azotetrazolate salts and complexes should be stored in a conductive rubber cup.

Sodium 5,5-azotetrazolate pentahydrate

To the solution of 5-aminotetrazole (8.5 g, 0.1 mol) and sodium hydroxide (35.0 g, 0.9 mol) in 150 mL of water, a moderate stream of gaseous chlorine (22 g, 0.3 mol) was introduced for 60 min. The reaction is strongly exothermic and it is cooled with ice brine to keep the temperature between -10 and 0°C. After the chlorine addition, the reaction was stirred for an additional 30 min. The color turned yellow and the fine solid precipitated. Then the reaction mixture was warmed to 75 °C and slowly cooled to room temperature to allow the larger crystals to form. The precipitate was filtered, washed with distilled water, and air-dried to yield 10.7 g (71.4 %) of the yellow solid. Anal. Calcd. for $C_2H_{10}N_{10}Na_2O_5$ (%): C 8.00, H 3.36, N 46.67. Found: C 8.00, H 3.33, N 46.21. Water determined by Karl Fischer (%): Calcd. 30.0. Found 29.2. DTA: endotherm 1: 81 °C (onset), endotherm 2: 122 °C (onset), exotherm: 212 °C (start), 239 °C (maximum). FTIR (cm^{-1}): 3584m, 3538m, 3368s, 3243s, 3100sh, 2832w, 2447w, 2107w, 1684m, 1625s, 1480w, 1452w, 1400s, 1204m, 1165m, 1097w, 1065m, 1044w, 926vw, 774m 739s 730s, 559sh, 508m.

Imidazole 5,5'-azotetrazolate monohydrate (ImAzTz·H_2O)

Sodium 5,5'-azotetrazolate pentahydrate (3.0 g, 10 mmol) was dissolved in 25 mL of water at 70 °C. A solution of imidazole hydrochloride (2.5 g, 24 mmol) in 5 mL of water was added dropwise and the reaction mixture was stirred for 30 minutes. After cooling to room temperature, the solid was filtered, washed with water, and air-dried to yield 3.0 g (94%) of yellow needles. Anal. Calcd. for $C_8H_{12}N_{14}O$ (%): C 30.00, H 3.78, N 61.23. Found: C 30.22, H 3.67, N 61.24. DTA: endotherm: 117°C (start), 119°C (onset), 148°C (minimum), exotherm: 164°C (start), 166°C (onset), 167.5°C (maximum). Density: 1.45 g.cm^{-3} (1.48 g.cm^{-3} [6]). FTIR (cm^{-1}) 3114m, 3031w, 2952m, 2742m, 2644m, 1933m, 1798w, 1742w, 1698w, 1666w, 1589m, 1538w, 1461m, 1393m, 1321m, 1228w, 1195m, 1162w, 1130w, 1101w, 1059m, 1000m, 903w, 866s, 784s, 733s, 694w, 635s, 563m.

3-Amino-1,2,4-triazole 5,5'-azotetrazolate (ATrAzTz)

Sodium 5,5'-azotetrazolate pentahydrate (3.0 g, 10 mol) was dissolved in 50 mL of water at 40 °C. A solution of 3-amino-1,2,4-triazole (2.52 g, 30 mmol) with hydrochloric acid (35%, 3.15 g, 30 mmol) in 10 mL of water was added dropwise. The mixture was stirred for 30 minutes, then cooled. The light-yellow precipitate was filtered, washed with distilled water, and air-dried to yield 3.3 g (99%) of yellow solid. Anal. Calcd. for $C_6H_{10}N_{18}$ (%): C 21.56, H 3.02, N 75.43. Found: C 21.82, H 2.83, N 75.35. DTA: exotherm: 163°C (start), 169°C (onset),

169.5°C (maximum). Density: 1.63 g.cm^{-3}. FTIR (cm^{-1}) 3318w, 3276w, 3117m, 2975w, 2903w, 2662w, 2449m, 1783w, 1675s, 1632w 1570m, 1476m, 1458m, 1408s, 1261w, 1202w, 1161w, 1147m, 1115w, 1050m, 1037m, 1014m, 937s, 871m, 772m, 727sh, 636sh, 560s, 531sh, 426s.

3,5-Diamino-1,2,4-triazole 5,5'-azotetrazolate (DATrAzTz)

3,5-Diamino-1,2,4-triazol [7] (4 g, 40 mmol) was suspended in 25 mL of water and hydrochloric acid (35%, 4.2 g, 40 mmol) was added. The mixture was heated to 50 °C and stirred for 10 min, then cooled to 30 °C and a solution of sodium 5,5'-azotetrazolate pentahydrate (3 g, 10 mmol) in 50 mL of water was slowly added. The mixture was stirred for 10 min, cooled to 10°C, the precipitate was filtered, washed with 10 mL of cold water, and air-dried to yield 3.55 g (97.5%) of yellow solid. Anal. Calcd for $C_6H_{10}N_{20}$ (%): C 19.78, H 3.32, N 76.90. Found: C 19.88, H 3.34, N 76.29. DTA: exotherm: 195°C (start), 200°C (onset), 200.5°C (maximum). Density: 1.63 g.cm^{-3}. FTIR (cm^{-1}): 3405sh, 3332m, 3209s, 3139m, 3032w, 2974w, 2911w, 2634sh, 1690sh, 1650s, 1638s, 1611sh, 1546m, 1480w, 1401s, 1365m, 1208m, 1180w, 1152w, 1072w, 1049m, 1037m, 1011m, 841s, 785s, 768w, 736s, 665m, 563sh, 511sh, 479w, 442m. The crystals suitable for X-ray analysis were prepared by silica acid gel method described in general methods.

5,5´-Methylenebis(3-amino-1*H*-1,2,4-triazol) 5,5'-azotetrazolate monohydrate (MBATrAzTz·H₂O)

5,5´-Methylenebis(3-amino-1*H*-1,2,4-triazol) [8-9] (11.7 g, 64.9 mmol) was suspended in 110 mL of water, hydrochloric acid (35%, 13.5 g, 130 mmol) was added dropwise and the mixture was stirred for 2 h until a clear solution was obtained. Then, a solution of sodium 5,5'-azotetrazolate pentahydrate (9.8 g, 32.6 mmol) in 110 mL of water was prepared, heated to 50 °C, and the 5,5´-Methylenebis(3-amino-1*H*-1,2,4-triazole) dihydrochloride solution was gradually added. A yellow precipitate formed immediately, the mixture was stirred at 50 °C for 10 min, then cooled to 10°C. The precipitate was filtered, washed with water, and air-dried to yield 11.4 g. (95.8 %) Anal. Calcd for $C_7H_{12}N_{18}O$ (%): C 23.08, H 3.32, N 69.21. Found: C 23.22, H 3.44, N 68.69. DTA: exotherm: 134°C (start), 144°C (onset), 144.5°C (maximum). Density: 1.71 g.cm^{-3}. FTIR (cm^{-1}): 3403m, 3176m, 3073m, 2772m, 1690s, 1672s, 1621m, 1585m, 1503w, 1432w, 1396m, 1312m, 1239m, 1202m, 1182m, 1152w, 1104w, 1038m, 1029m, 1016m, 994m, 928w, 808m, 772sh, 745m, 736m, 708m, 672m, 647m, 564m, 461m.

3,5-Diamino-*N*-amidino-1-guanyl-1,2,4-triazole 5,5´-azotetrazolate (DAAmGTrAzTz)

3,5-Diamino-1,2,4-triazole (2.6 g, 26 mmol) hydrochloric acid (35%, 2,7 g, 26 mmol) and cyanoguanidine (2.2 g, 26 mmol) were dissolved in 250 mL of ethanol and heated under reflux for 6 h. After cooling, the solvent was removed under reduced pressure at 40 °C, and the solid residue was dissolved in 90 mL of water. A solution of sodium 5,5'-azotetrazolate pentahydrate (3.8 g, 12.6 mmol) in 100 mL of water was added, the precipitate was filtered, washed with water, and air-dried to yield 4.13 g (93.4 %) of yellow solid. Anal. Calcd for $C_6H_{11}N_{19}$ (%): C 20.63, H 3.17, N 76.19. Found: C 19.83, H 3.40, N 74.27. DTA: exotherm: 171°C (start), 176.5°C (onset), 190°C (maximum). Density: 1.59 g.cm^{-3}. FTIR (cm^{-1}): 3404m, 3310m, 3281m, 3209m, 3009m, 2766m, 1710m, 1698m, 1665s, 1590w, 1548m, 1459m, 1398m, 1254w, 1208m, 1163w, 1045m, 1034m, 1004m, 929w, 792m, 773m, 738s, 721m, 685s, 624m,

568m, 414s. By crystallization of 6 by silica acid gel method in order to prepare the crystals suitable for X-ray analysis, 1,2,4]triazolo[1,5-a][1,3,5]triazine-2,5,7-triamine was formed instead.

Hexamethylenetetramine 5,5'-azotetrazolate monohydrate (HMTAzTz·H_2O)

Sodium 5,5'-azotetrazolate pentahydrate (3.0 g, 10 mmol) was dissolved in 25 mL of water and a solution of hexamethylenetetramine (5.61 g, 40 mmol) with hydrochloric acid (35%, 4.2 g, 40 mmol) in 25 mL of water was added dropwise at 70°C. After 90 minutes of stirring, the product was cooled, filtered, and air-dried to yield 6.77 g (87.5%). Anal. Calcd for $C_{14}H_{28}N_{18}O$ (%): C 36.20, H 6.08, N 54.28. Found: C 37.10, H 6.10, N 50.53. DTA: endotherm: 120°C (start), 123°C (onset), 127°C (minimum), exotherm: 149°C (start), 153°C (onset), 154.5°C (maximum). Density: 1.44 g.cm^{-3}. FTIR (cm^{-1}) 3489w, 3401w, 2916w, 2816w, 2774w, 2632w, 2555w, 2487w, 2410w, 2356w, 2324w, 1605w, 1466m, 1431w, 1389m, 1366w, 1295m, 1258m, 1210m, 1153w, 1058m, 1007s, 978s, 863m, 818s, 794m, 771m, 731m, 692w, 658s, 562w, 523w, 505w, 493m. The crystals suitable for X-ray analysis were prepared by the procedure when 1:1 molar ratio of starting materials was used, followed by standing the reaction mixture overnight.

Oxalyldihydrazide 5,5′-azotetrazolate trihydrate (ODHAzTz·3H_2O)

Sodium 5,5'-azotetrazolate pentahydrate (4.0 g, 13.4 mmol) and oxalyldihydrazide hydrochloride [10] (6.2 g, 40 mmol) were suspended in 240 mL of water, and the mixture was stirred at room temperature for 1 h. The yellow solid was filtered, washed with 50 mL of water, and air-dried to yield 5.98 g (98.3 %). Anal. Calcd. for $C_6H_{20}N_{18}O_7$ (%): C 15.79, H 4.42, N 55.25. Found: C 15.62, H 4.37, N 54.82. DTA: exotherm: 88°C (start), 93°C (maximum). Density: 1.60 g.cm^{-3}. FTIR (cm^{-1}): 3177m, 2939m, 2748m, 2562m, 2458m, 2324w, 2162w, 2129m, 2051m, 1705s, 1634m, 1556s, 1532s, 1462sh, 1407s, 1289m, 1251m, 1221m, 1206s, 1176m, 1163m, 1097w, 1067w, 1052w, 1026w, 996w, 783sh, 734s, 721s, 640s, 601s, 563s, 513s, 439m.

1,1'-Dimethylbiguanide 5,5'-azotetrazolate hydrate (DMBGAzTz·H_2O)

Sodium 5,5'-azotetrazolate pentahydrate (3.0 g, 10.0 mmol) was dissolved in 25 mL water at 70 °C and a solution of 1,1'-dimethylbiguanide hydrochloride (6.6 g, 39.9 mmol) in 10 mL of water was added dropwise. After 90 minutes of stirring, the product was cooled, filtered, and air-dried to yield 4.04 g (91.3%) of yellow crystalline material. Anal. Calcd. for $C_{10}H_{26}N_{20}O$ (%): C 27.15, H 5.92, N 63.31. Found: C 25.84, H 5.90, N 60.75. DTA: endotherm: 121°C (start), 152°C (minimum), exotherm: 182°C (start), 193°C (maximum). Density: 1.35 g.cm^{-3}. FTIR (cm^{-1}) 3479w, 3368m, 3288m, 3093sh, 2439w, 1669w, 1620m, 1567s, 1487sh, 1416m, 1394s, 1284w, 1195w, 1158w, 1110w, 1081w, 1057m, 1044sh, 934m, 843w, 775m, 728sh, 637s, 609sh, 586sh, 557s, 536s, 515s, 471s, 439sh, 420s. The crystals suitable for X-ray analysis were prepared by the procedure when 1:1 molar ratio of starting materials was used, followed by standing the reaction mixture overnight.

Glyoxal bis(amidinohydrazone) 5,5'-azotetrazolate dihydrate (GBAmHAzTz·2H_2O)

Aminoguanidine hydrochloride (3.0 g, 27 mmol) was dissolved in 7.5 mL of water, and 40% water solution of glyoxal (1.9 g, 13 mmol) was added dropwise. The mixture was stirred for 20 min, then heated to 60 °C, and a solution of sodium 5,5'-azotetrazolate pentahydrate (4.1 g, 13.7 mmol) in 30 mL of hot water was added. The mixture was stirred for 10 min, the precipitate was filtered, washed with 20 mL of water, and air-dried to yield 4.7 g (96%) of yellow solid. Anal. Calcd for $C_6H_{16}N_{18}O_2$ (%): C 19.36, H 4.33, N 67.77. Found: C 19.62, H 4.38, N 67.77. DTA: exotherm: 185°C (start), 197°C (onset), 199°C (maximum). Density: 1.59 g.cm^{-3}. FTIR (cm^{-1}): 3589w, 3511w, 3356m, 3180m, 3059m, 3006m, 2861m, 2773m, 2423w 1692s, 1658m, 1618s, 1583sh, 1467m, 1395s, 1313w, 1200m, 1151s, 1114w, 1089m, 1075m, 1048m, 1034m, 1011w, 947m, 920w, 902w, 769m, 737s, 634s, 604s, 576s, 548s, 513s, 476sh, 442m. The crystals suitable for X-ray analysis were prepared by silica acid gel method described in general methods.

Glyoxal bis(aminoamidinohydrozone) 5,5'-azotetrazolate dihydrate (GBAAmHAzTz·2H_2O)

Diaminoguanidine hydrochloride (6.0 g, 48 mmol) was dissolved in 15 mL of water, and 40% glyoxal (3.4 g, 24 mmol) was added dropwise for 20 min. The mixture was then heated to 60 °C, and a saturated solution of sodium 5,5'-azotetrazolate pentahydrate (3.6 g, 12 mmol) in water was added dropwise. After that, the mixture was stirred at 60 °C for 10 min, cooled, the precipitate was filtered, washed with 50 mL of water and air-dried to yield 4.75 g (98.5%). Anal. Calcd for $C_6H_{18}N_{20}O_2$ (%): C 17.91, H 4.51, N 69.63. Found: C 18.13, H 4.55, N 64.41. DTA: exotherm: 160°C (start), 166°C (onset), 167°C (maximum). Density: 1.54 g.cm^{-3}. FTIR (cm^{-1}): 3172m, 1691m, 1629s, 1585s, 1390s, 1294m, 1191m, 1112m, 1077m, 1036m, 936m, 770m, 733s, 558s.

Guanidine 5,5'-azotetrazolate (GZT)

Compound was prepared according to Labaj et al. [11]. Yield 72%. Anal. Calcd. for $C_4H_{12}N_{16}$ (%): C 16.90, H 4.22, N 78.87 Found: C 16.80, H 4.12, N 76.48. DTA: exotherm: 215°C (start), 244.5°C (maximum). FTIR (cm^{-1}): 3437m, 3386m, 3318m, 3190m, 3078m, 2825w, 2428w, 1694w, 1647s, 1583w, 1568m, 1454w, 1397sh, 1196m, 1174w, 1160w, 1138w, 1080w, 1048m, 1039w, 1010w, 768m, 734sh, 687w, 569w, 490s, 433s

Triaminoguanidine 5,5'-azotetrazolate (TAGZT)

Sodium 5,5'-azotetrazolate pentahydrate (3.0 g, 10 mol) was dissolved in 175 mL of methanol at 55 °C. A hot solution of triaminoguanidine nitrate (3.5 g, 21 mmol) in 15 mL of water was added dropwise. The mixture was stirred for 30 minutes, then cooled. The precipitate was filtered, washed with distilled water, and air-dried to yield 2,8 (75%). Anal. Calcd. for $C_4H_{18}N_{22}$ (%): C 12.83, H 4.85, N 82.32 Found: C 13.04, H 4.71, N 80.79. DTA: exotherm: 180°C (start), 188°C (maximum). FTIR (cm^{-1}): 3351w, 3333m, 3199m, 2799w, 2398w, 1674s, 1630m, 1596w, 1567w, 1435w, 1385m, 1332m, 1186m, 1160w, 1135s, 1068w, 1033m, 990s, 941s, 770m, 730sh, 636sh, 584s, 559s, 444m, 418s

Triaminoguanidine nitrate (TAGN)

Compound was prepared according to Wagaman, Clark and Henderson (example 8) [12]. Yield (65%). Anal. Calcd. for $C_1H_9N_7O_3$ (%): C 7.19, H 5.43, N 58.67 Found: C 6.98, H 5.26, N 59.27. DTA: endotherm: 216°C (start), 221.5°C (maximum), exotherm: 223°C (maximum)[1]. FTIR (cm^{-1}): 3342m, 3230m, 2451w, 2324w, 1681sh, 1378m, 1300s, 1199w, 1132m, 1051m, 963sh, 926sh, 823m, 640w, 575sh, 430m.

Preparation of crystals for X-ray analysis

The single crystals suitable for X-ray analysis were prepared in silica acid gel by modified method [13-15]. Sodium silica (36-38%, 10 mL) and water (100 mL) were mixed to obtain a solution with a density of 1.03 $g.cm^{-3}$, and sodium azotetrazolate pentahydrate (1 g, 3,3 mmol) was added. After dissolving, the batch was divided into 33 portions, transferred into 10 mL bottles, 0,1 mL of 45% acetic acid was added, the solution was stirred, the bottles were closed, and they were let stand for 24 hours. Then, an aqueous solution at a concentration of about 0,5-1 % of the hydrochloride of the desired cationic base is added to the gel surface. After several weeks or months, the material suitable for X-ray analysis crystallizes. It was used for compounds **DATrAzTz** and **GBAmHAzTz·4H$_2$O**; in the case of attempted crystallization of **DAAmGTrAzTz**, the condensed product, [1,2,4]triazolo[1,5-a][1,3,5]triazine-2,5,7-triamine, was obtained instead of the expected **DAAmGTrAzTz**. **HMTAzTz·2H$_2$O** and **DMBGAzTz·2H$_2$O** single crystals were obtained directly from the reaction mixture.

[1] Melting with decomposition

3. Computational methods and supporting data

Heat of formation

A recently published method[16] for estimating the solid-state enthalpy of formation has proven highly promising and was successfully validated for guanidinium 5,5'-azotetrazolate (GZT).[17] However, since its application requires specific experimental precursor data and sublimation estimates that are not readily available for our newly prepared salts, we chose an alternative approach to ensure consistency across the dataset.

All quantum chemical calculations were performed using the ORCA program package (version 6.1.0) [18-19]. The geometries of isolated ions and molecules were optimized in the gas phase at the ωB97X-D4/def2-TZVPP level of theory. The RIJCOSX approximation with the def2/J auxiliary basis set was employed to accelerate these structural optimizations. To obtain highly accurate thermodynamic data, single-point electronic energies were subsequently calculated on the optimized geometries using the DLPNO-CCSD(T) method [20-25]. These electronic energies were extrapolated to the complete basis set (CBS) limit using the two-point extrapolation scheme Extrapolate(3/4,def2). The auxiliary basis sets required specifically for the resolution of identity (RI) approximation within the DLPNO framework were generated automatically via the AutoAux procedure [26].

Gas-phase enthalpies of formation $\Delta_f H^\circ$(g) were determined using the atomization energy method. First, the electronic energies of isolated atoms in their ground states were computed at the CCSD(T)/CBS level, with the exception of the hydrogen atom, where the Hartree-Fock (HF) method coupled with the CBS extrapolation was applied. The calculated energies and thermal corrections for the atoms are listed in Table S1.

Table S1. Calculated electronic enthalpies for atoms C, H, N and O and their literature experimental.

Atom	H^{ORCA}(atom) [Hartree]*	$\Delta_f H^\circ$(atom, g) [kJ mol^{-1}] [27]
C	-37.78751386	716.68
H	-0.497646691	217.998
N	-54.52839087	472.68
O	-75.00272678	249.17

*1 Hartree = 2625.50184 kJ.mol^{-1}

The gas-phase enthalpies of formation for the individual ions were then calculated based on the atomization reaction utilizing the following equation:

$$\Delta_f H^\circ(\text{ion, g}) = H^{\text{ORCA}}(\text{ion}) - \sum H^{ORCA}(\text{atom}) + \sum \Delta_f H^\circ(\text{atom, g}) \quad (1)$$

The resulting gas-phase enthalpies of formation for all investigated cations and anions are summarized in Table S2.

Table S2. Calculated electronic enthalpies for atoms C, H, N and O and their literature experimental.

Ion	H^{ORCA}(ion) [Hartree]	$\Delta_f H^\circ$(ion, g) [kJ mol^{-1}]
**$AzTz^{2-}$**	-622.8676504	886.28
**Im^{+}**	-226.2193543	741.26
**ATr^{+}**	-297.544232	823.94
**$DATr^{+}$**	-352.8418522	801.58
**$MBATr^{2+}$**	-633.0750414	1 841.92
**$DAAmGTr^{2+}$**	-650.3508959	1 717.90
**HMT^{+}**	-454.4309789	825.53
**ODH^{+}**	-448.9965878	572.58
**$DMBG^{+}$**	-432.5254879	595.20
**$GBAmH^{2+}$**	-596.1853572	1 817.12
**$GBAAmH^{2+}$**	-706.6877962	2 016.05
**NO_3^- anion**	-280.1114310	-289.10

For anhydrous energetic salts, the solid-state enthalpies of formation $\Delta_f H^\circ$(s) were derived from the respective gas-phase ion enthalpies and the calculated lattice enthalpies $\Delta_{lat} H^\circ$. The $\Delta_{lat} H^\circ$ values were estimated using the volume-based thermodynamics (VBT) approach parameterized by Habert et al. [28] using SMILE strings of salts.

$$\Delta_f H^\circ(\text{anhydr. salt, s}) = \Delta_f H^\circ(\text{AzTZ}^{2-}, \text{g}) + n\, \Delta_f H^\circ(\text{cation, g}) - \Delta_{lat} H^\circ(\text{salt}) \quad (2)$$

For hydrated energetic salts, the standard solid-state enthalpy of formation was estimated by applying the Thermodynamic Solvate Difference Rule [29] to the computed enthalpy of the anhydrous parent salt. According to this approach [30], each molecule of crystal water contributes a constant empirical increment of -298.6 kJ mol^{-1}, allowing the hydrate's enthalpy to be calculated via the following equation:

$$\Delta_f H^\circ(\text{salt}\cdot n\text{H}_2\text{O, s}) = \Delta_f H^\circ(\text{salt, s}) - 298.6\, n \quad (3)$$

The calculated lattice parameters and final solid-state enthalpies are provided in Table S3.

Table S3. Calculated electronic enthalpies for atoms C, H, N and O and their literature experimental.

Compound	$\Delta_f H^\circ$(salt, g) [kJ mol^{-1}]	$\Delta_{lat} H^\circ$(salt) [kJ mol^{-1}]	$\Delta_f H^\circ$(anhydr. salt, s) [kJ mol^{-1}]	$\Delta_f H^\circ$(salt·nH_2O, s) [kJ mol^{-1}]
**ImAzTz·H$_2$O**	2368.8	1554.6	814.2	515.6
**ATrAzTz**	2534.2	1575.8	958.4	
**DATrAzTz**	2489.4	1603.7	885.7	
**MBATrAzTz·H$_2$O**	2728.2	2017.2	711.0	412.4
**DAAmGTrAzTz**	2604.2	2009.9	594.3	
**HMTAzTz·H$_2$O**	2537.3	1347.3	1 190.0	891.4
**ODHAzTz·3H$_2$O**	2031.4	1464.1	567.3	-328.5
**DMBGAzTz·H$_2$O**	2076.7	1462.3	614.4	315.8
**GBAmHAzTz·2H$_2$O**	2703.4	2002.8	700.6	103.4
**GBAAmHAzTz·2H$_2$O**	2902.3	2011.8	890.5	293.3
**GZT**	2039.8	1580.1	459.8	-
**TAGZT**	2671.1	1602.4	1 068.7	-
**TAGN**	603.3	723.4	-120.1	-

Pyrotechnic characteristic calculation using REAL software

The prediction of combustion characteristics was conducted using the software REAL[31-32], which is a thermodynamic code that is used for computer simulation of chemical equilibrium in complex, chemically reacting systems. Experimentally measured enthalpies of formation, chemical formula, and component content in the mixture were used as input data. The adiabatic flame temperature and powder force (*FP*) were calculated at a constant pressure of 60 MPa, while the heat of combustion was calculated at a constant specific volume of 0.01 m^3 kg^{-1}. In all calculations, the virial equation of state was employed to account for gas non-ideality.

FP is calculated by the formula:

$$FP = n_g R T \quad (4)$$

, where n_g is the molar amount of gas product from 1 kg of composition, *R* is the universal gas constant, and *T* is the adiabatic flame temperature.

Calculations were performed for the pure synthesized compounds and reference compounds **GZT**, **TAGZT**, and **TAGN**. Additionally, calculations were performed for these compounds formulated into a pyrotechnic mixture based on a green propellant developed by Zigmund et al.[33]. This mixture consist of a 1:1 by mass ratio of NH_4ClO_4 and $Sr(NO_3)_2$, the respective high-nitrogen compound and sodium carboxymethylcellulose (NaCMC) as a binder. In all compositions, the oxidizer-to-fuel ratio was adjusted to achieve an oxygen balance +0.5% (consistent with the originally published green propellant). The propellant compositions used for calculation are shown in Table S4.

Table S4. Compositions of propellant with investigated compounds used for REAL calculation.

Compound in mixture	Propellant composition [wt %]			
	NH_4ClO_4	$Sr(NO_3)_2$	Compound	NaCMC
**ImAzTz·H$_2$O**	37.3	37.3	20.4	5.0
**ATrAzTz**	35.3	35.3	24.6	5.0
**DATrAzTz**	35.0	35.0	25.0	5.0
**MBATrAzTz·H$_2$O**	35.5	35.5	24.0	5.0
**DAAmGTrAzTz**	35.1	35.1	24.8	5.0
**HMTAzTz·H$_2$O**	39.4	39.4	16.2	5.0
**ODHAzTz·3H$_2$O**	31.3	31.3	32.4	5.0
**DMBGAzTz·H$_2$O**	38.0	38.0	19.0	5.0
**GBAmHAzTz·2H$_2$O**	34.8	34.8	25.4	5.0
**GBAAmHAzTz·2H$_2$O**	34.6	34.6	25.8	5.0
**GZT**	35.0	35.0	25.0	5.0
**TAGZT**	34.3	34.3	26.4	5.0
**TAGN**	26.8	26.8	41.2	5.0

The product composition of three selected propellant formulations was modeled at a constant specific volume of 0.01 $m^3\ kg^{-1}$ using the virial equation of state. To simulate final residue formation, the products were cooled to 298 K with the frozen expansion temperature set to 1500 K, to suppress further chemical reactions while allowing the condensation of the solid phase. Results are shown in Table S5.

Table S5. Calculated combustion products for reference **GZT** composition and composition with compound **ATrAzTz** with and without $Sr(NO_3)_2$.

**Compound**	**Propellant composition [wt %]**		
**NH_4ClO_4**	35.0	35.3	71.7
**$Sr(NO_3)_2$**	35.0	35.3	-
**GZT**	25.0	-	-
**ATrAzTz**	-	34.6	23.3
**NaCMC**	5.0	5.0	5.0
**Combustion product**	**Combustion product concentration [wt %]***		
**N_2 (g)**	28.51	27.32	26.12
**H_2O (g)**	22.25	19.24	25.16
**CO_2 (g)**	21.49	25.33	25.69
**O_2 (g)**	0.4835	0.4752	0.3463
**HCl (g)**	0.1326	0.1345	20.85
**Cl_2 (g)**	$3.261\ 10^{-5}$	$2.891\ 10^{-5}$	0.6035
**$SrCl_2$ (s)**	21.69	21.86	-
**$SrCO_3$ (s)**	4.218	4.24	-
**NaCl (s)**	1.121	1.121	1.121

*Only the product species with a concentration higher than 0.1% are shown, additionally with Cl_2.

Experimenta data and spectra

FTIR spectra

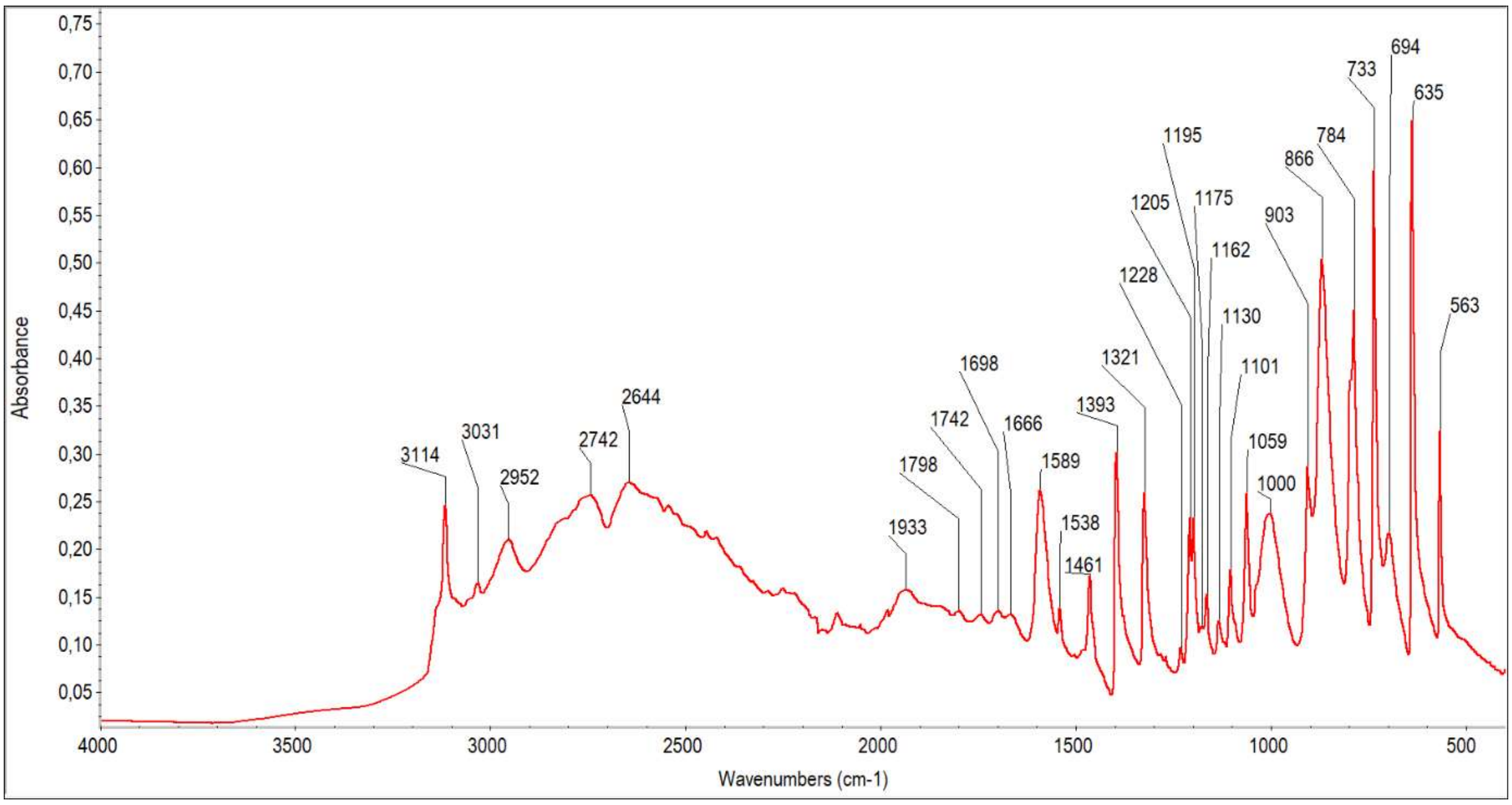

Figure S1. FTIR spectrum ImAzTZ·H_2O

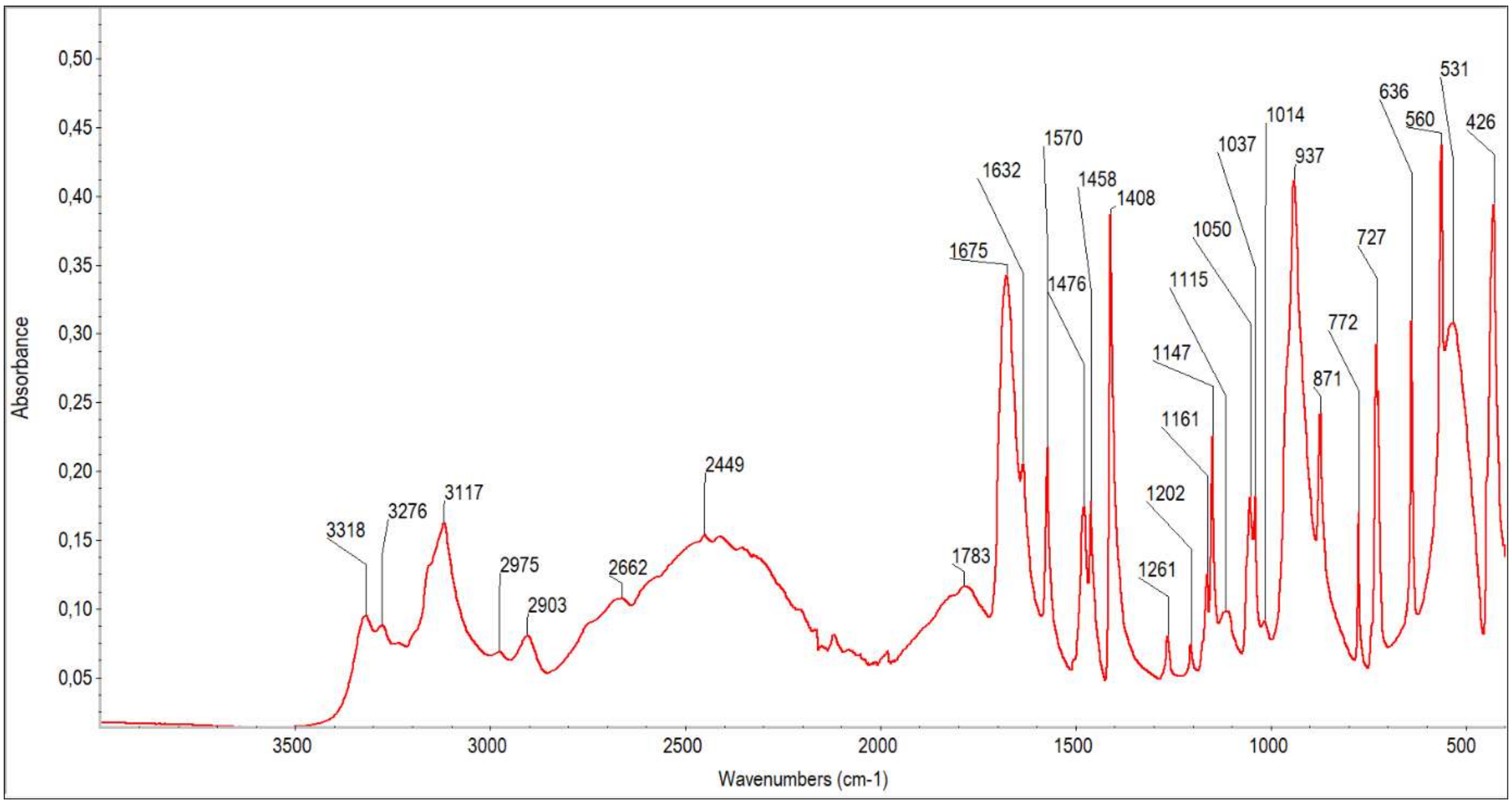

Figure S2. FTIR spectrum ATrAzTZ

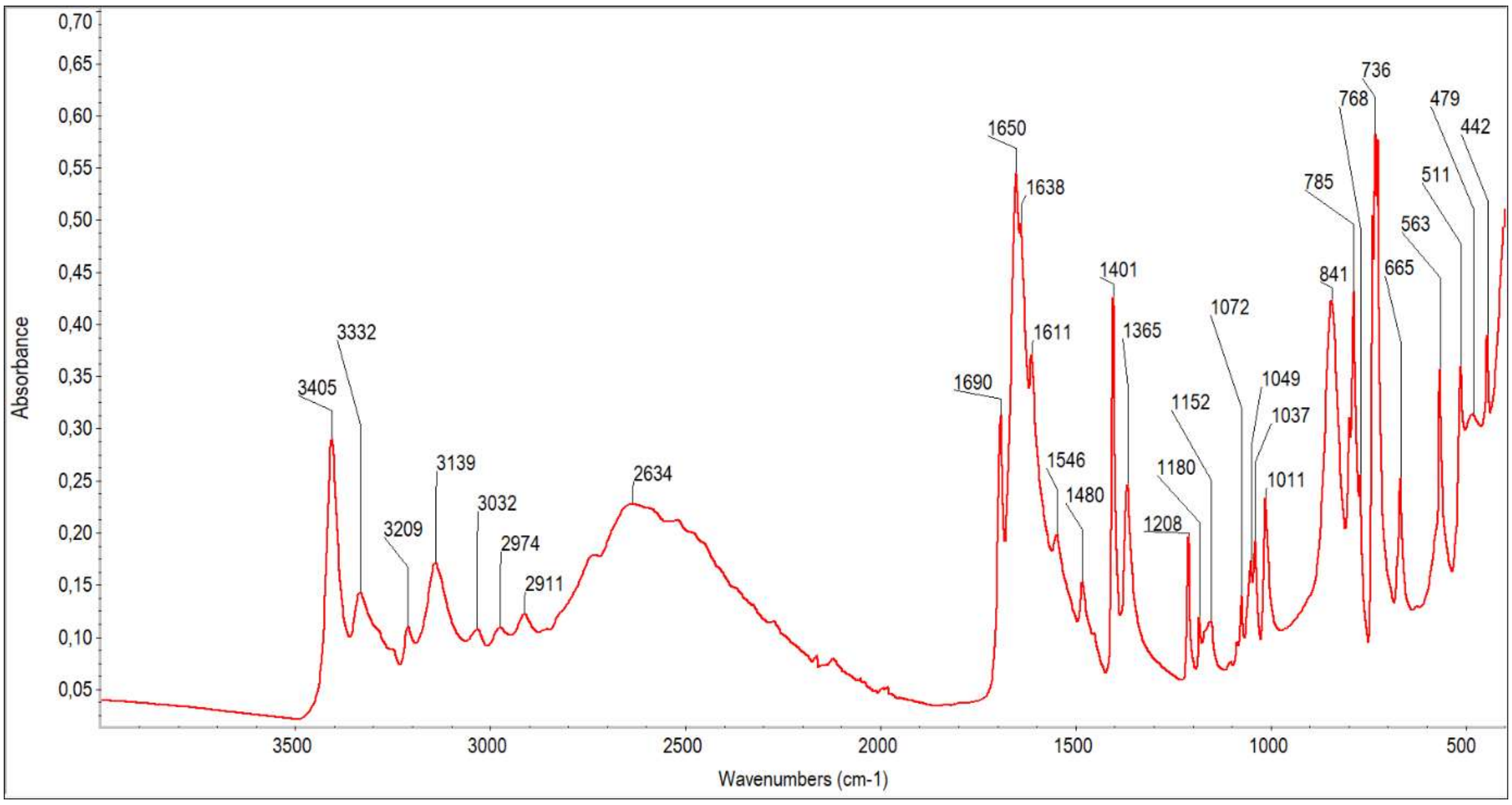

Figure S3. FTIR spectrum DATrAzTZ

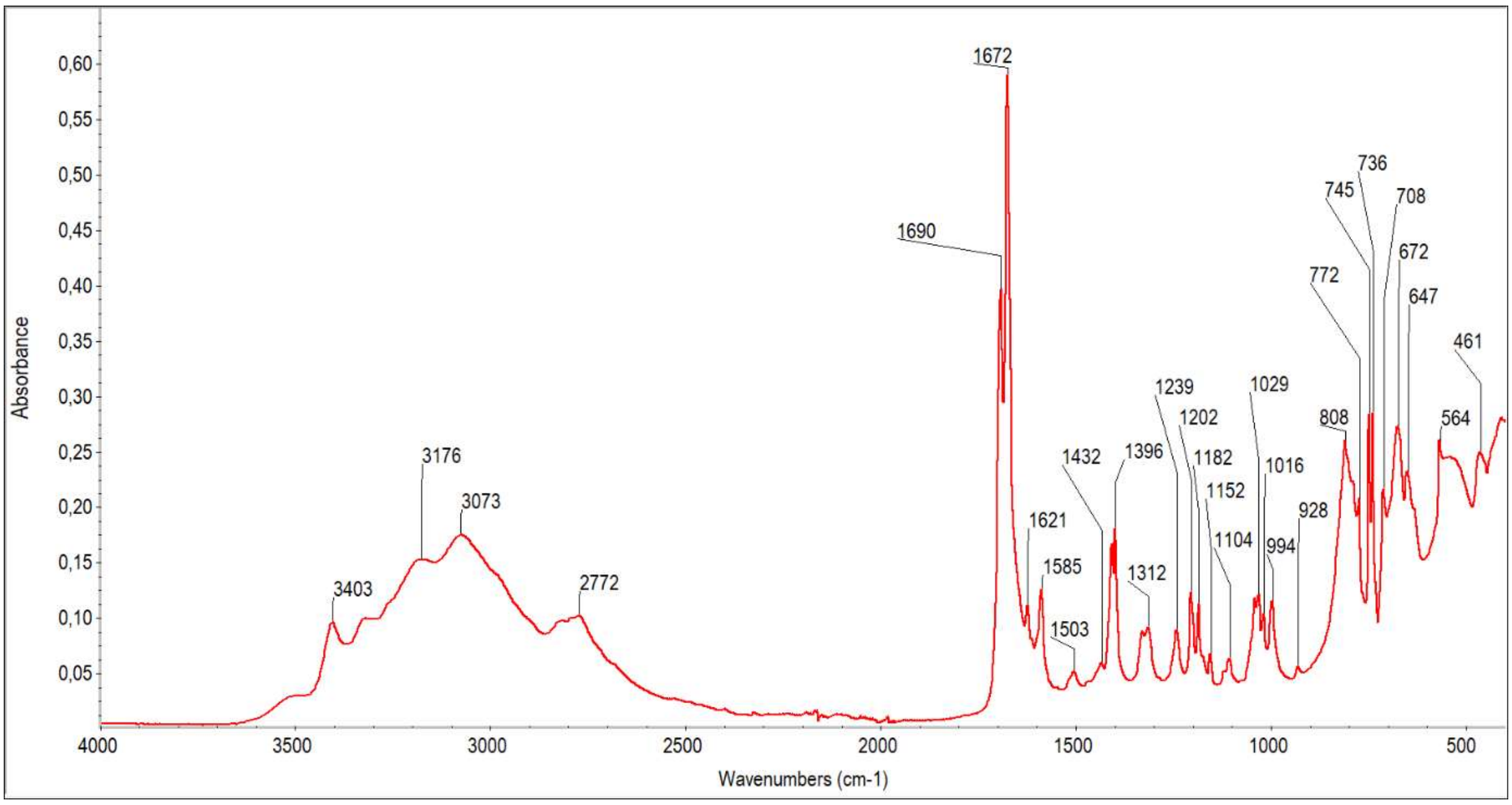

Figure S4. FTIR spectrum MBATrAzTZ·H_2O

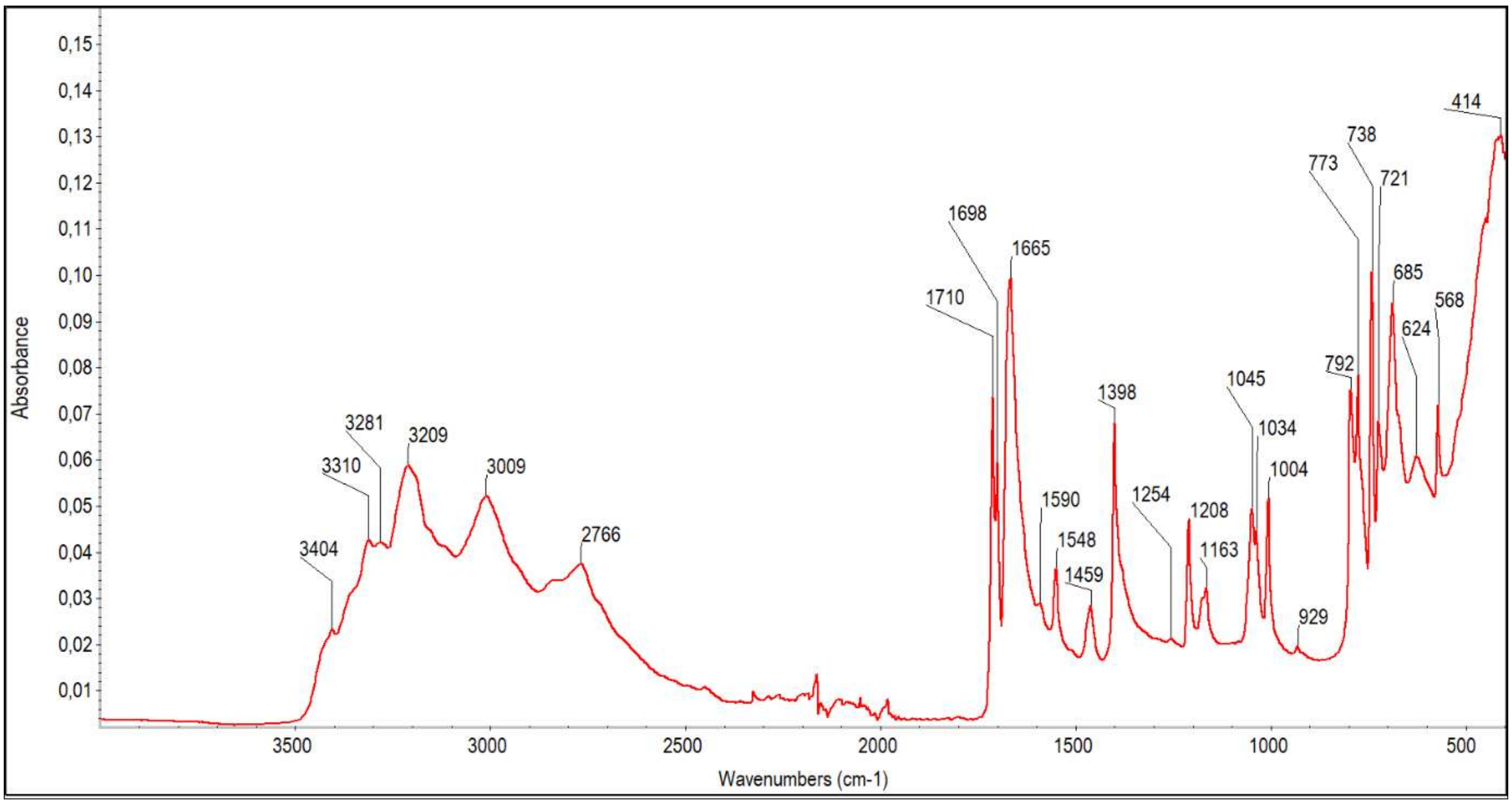

Figure S5. FTIR spectrum DAAmGTrAzTZ

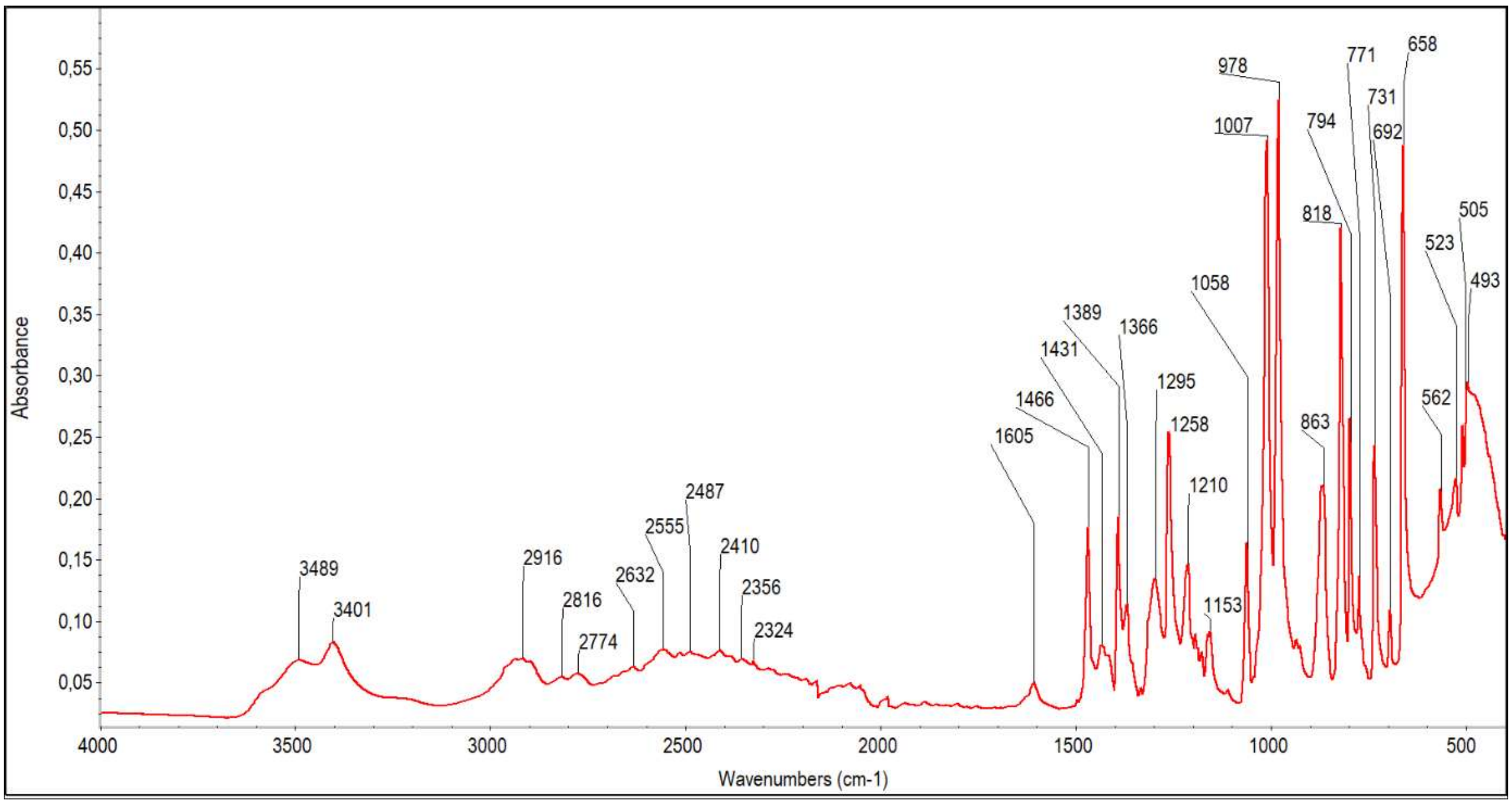

Figure S6. FTIR spectrum HMTAzTZ·H_2O

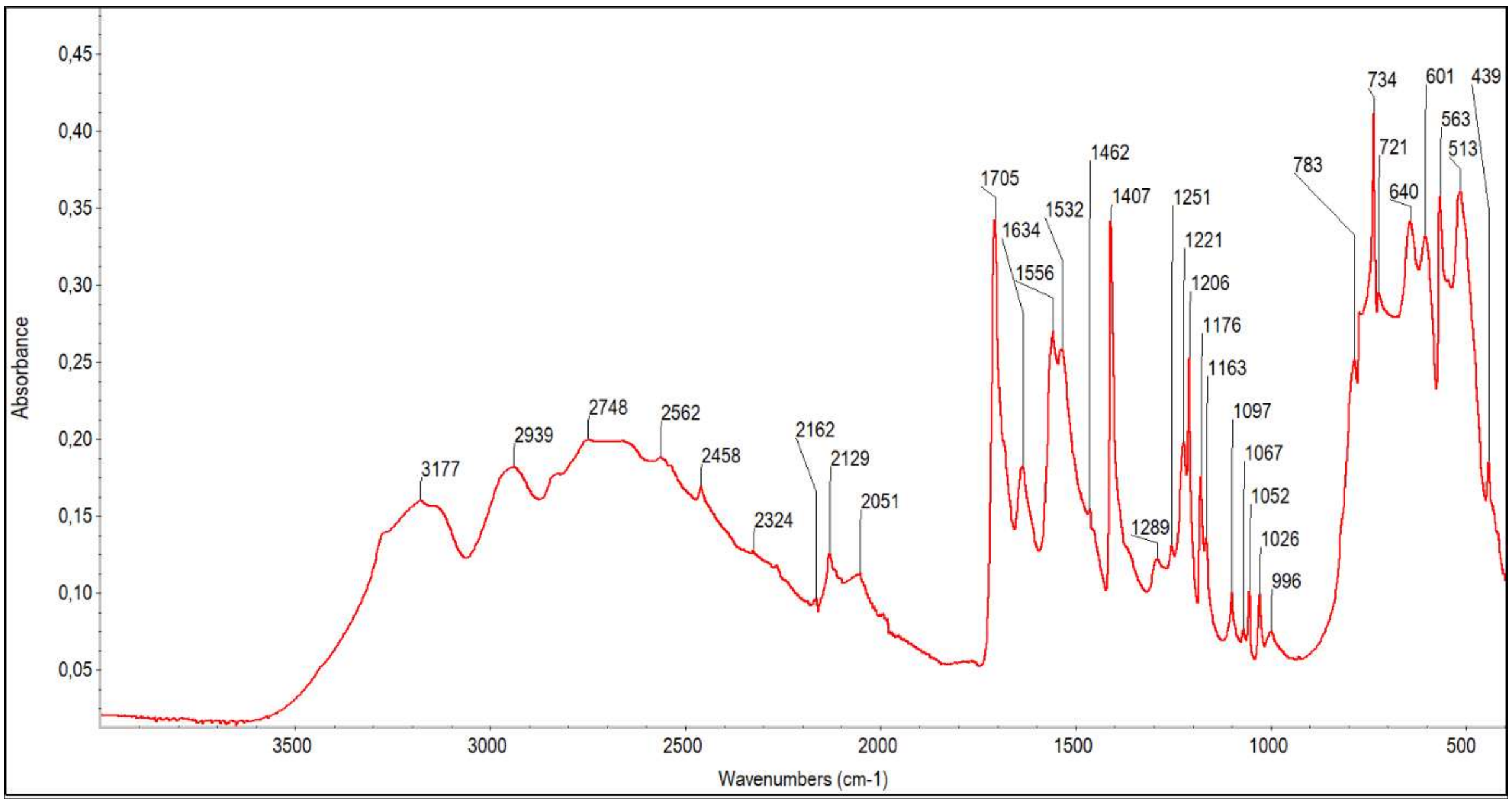

Figure S7. FTIR spectrum ODHAzTZ·3H_2O

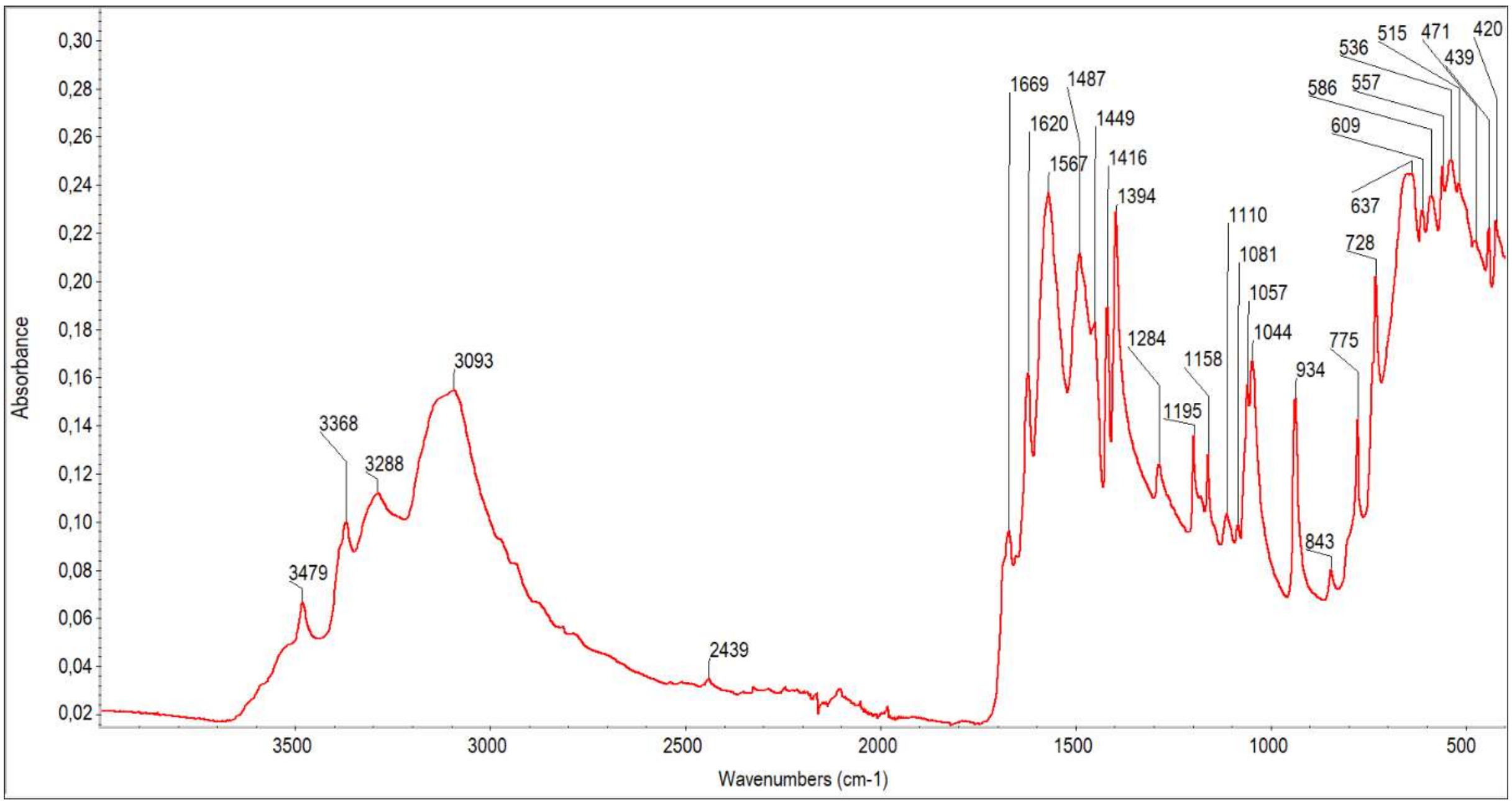

Figure S8. FTIR spectrum DMBGAzTZ·H_2O

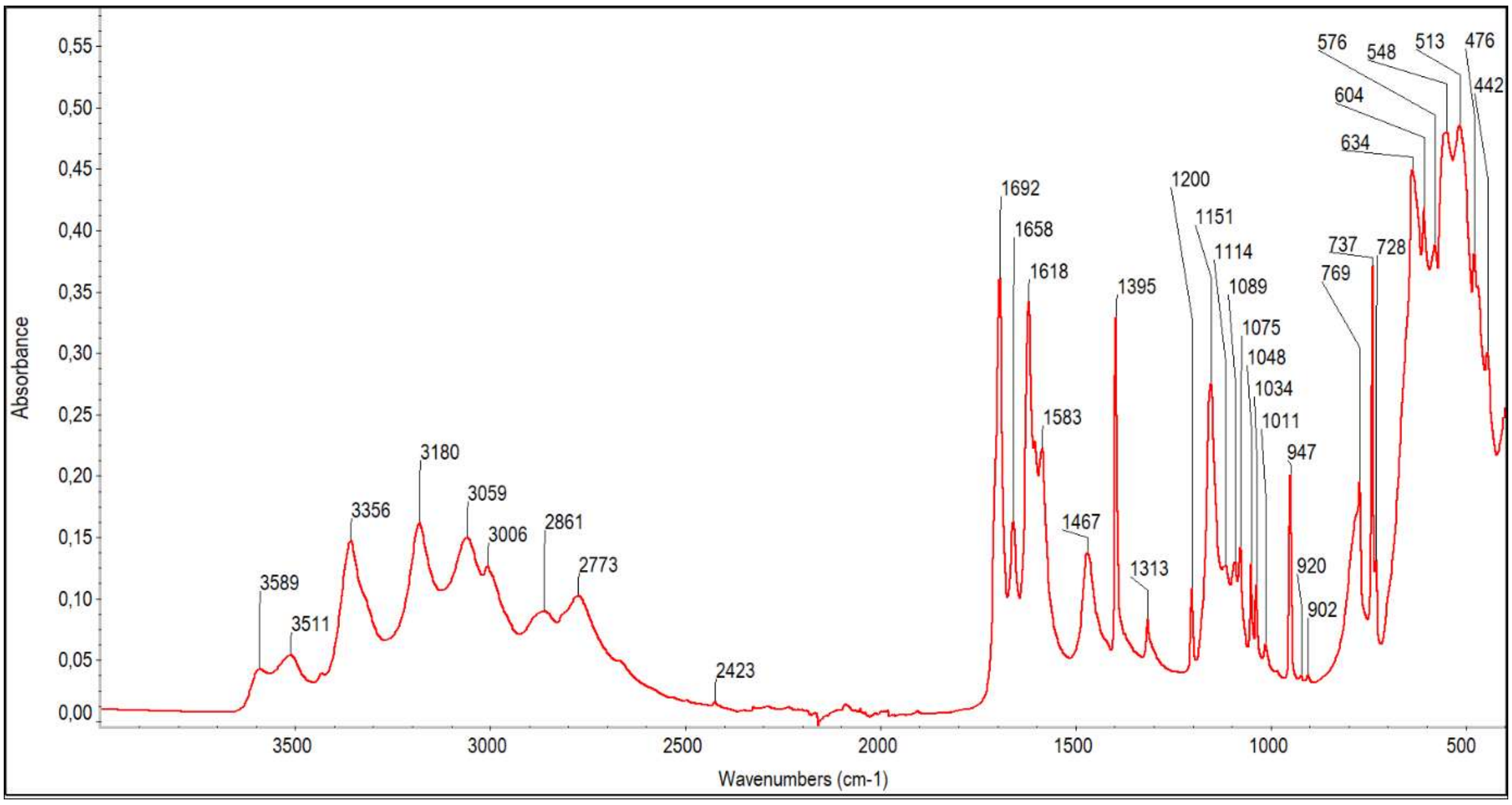

Figure S9. FTIR spectrum GBAmHAzTZ·$2H_2O$

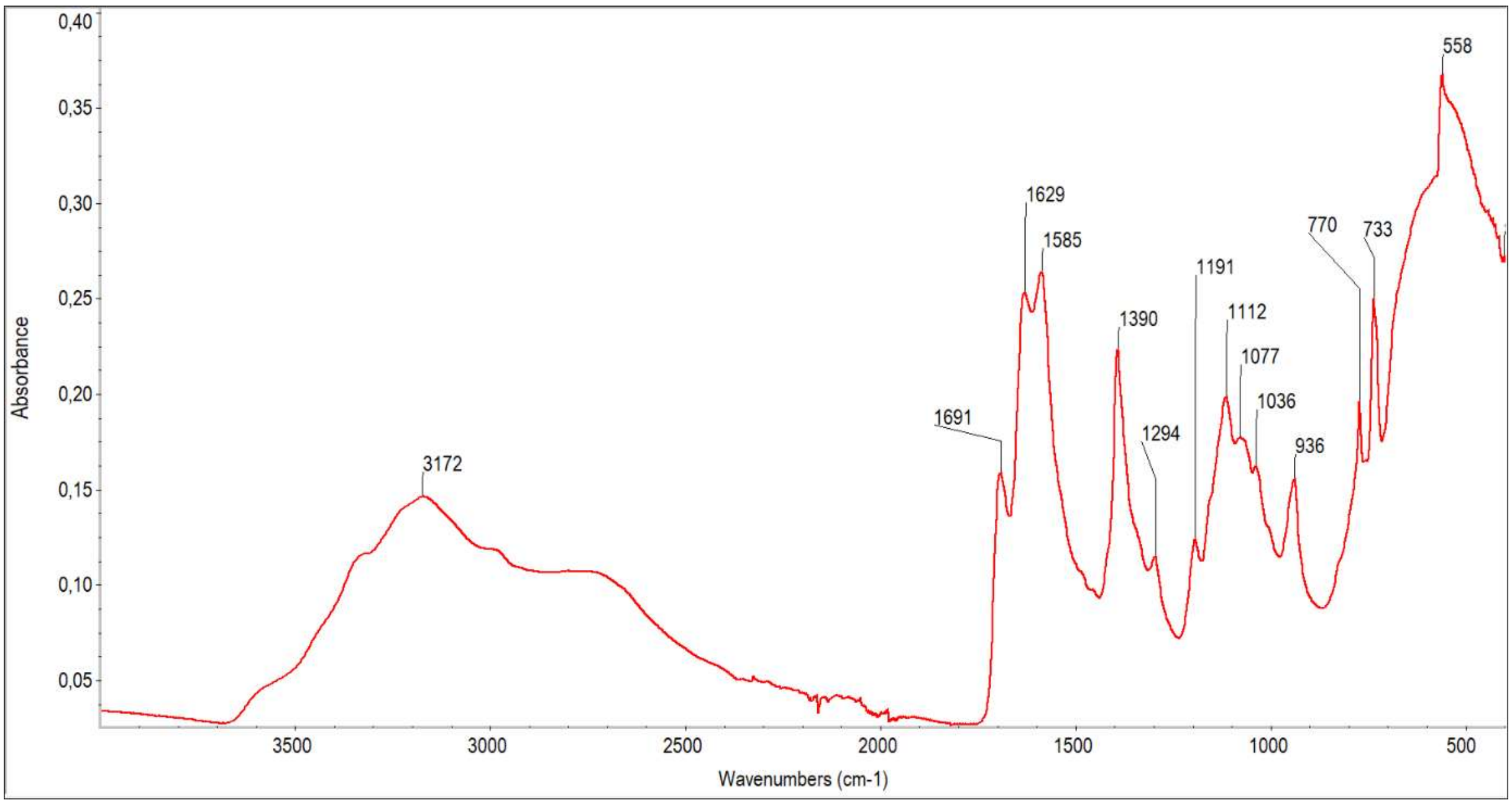

Figure S10. FTIR spectrum GBAAmHAzTZ·$2H_2O$

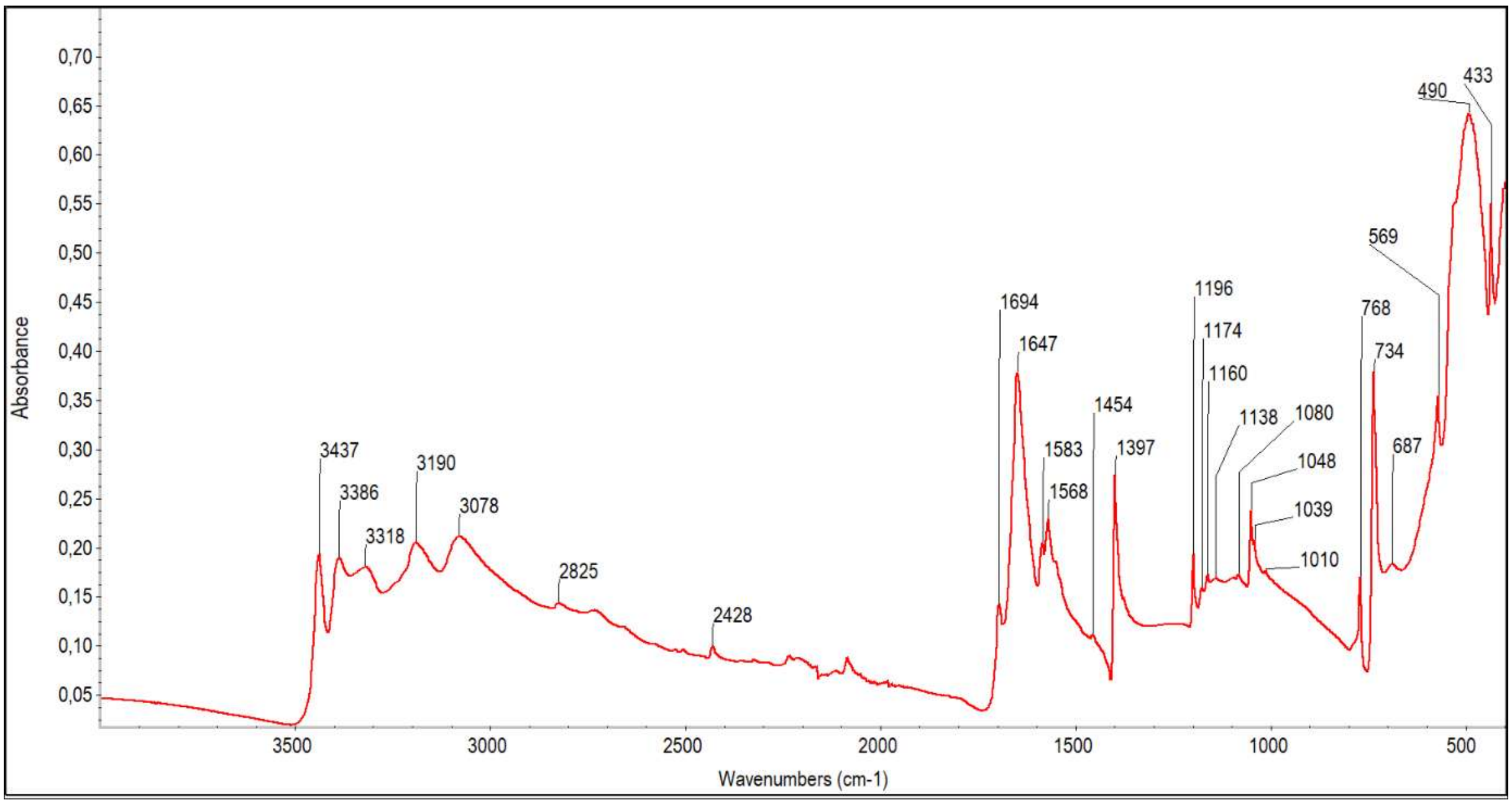

Figure S11. FTIR spectrum GZT

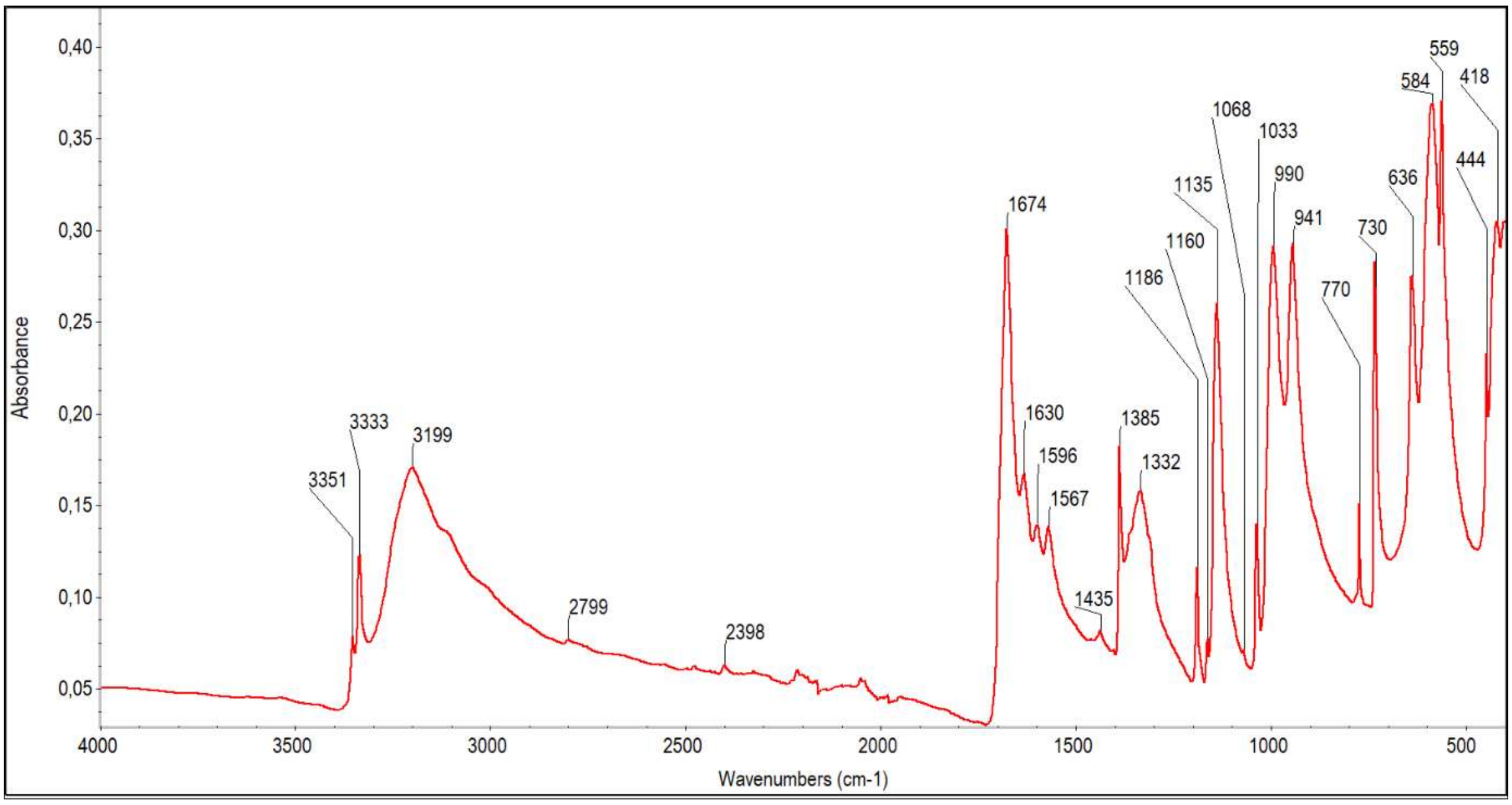

Figure S12. FTIR spectrum TAGZT

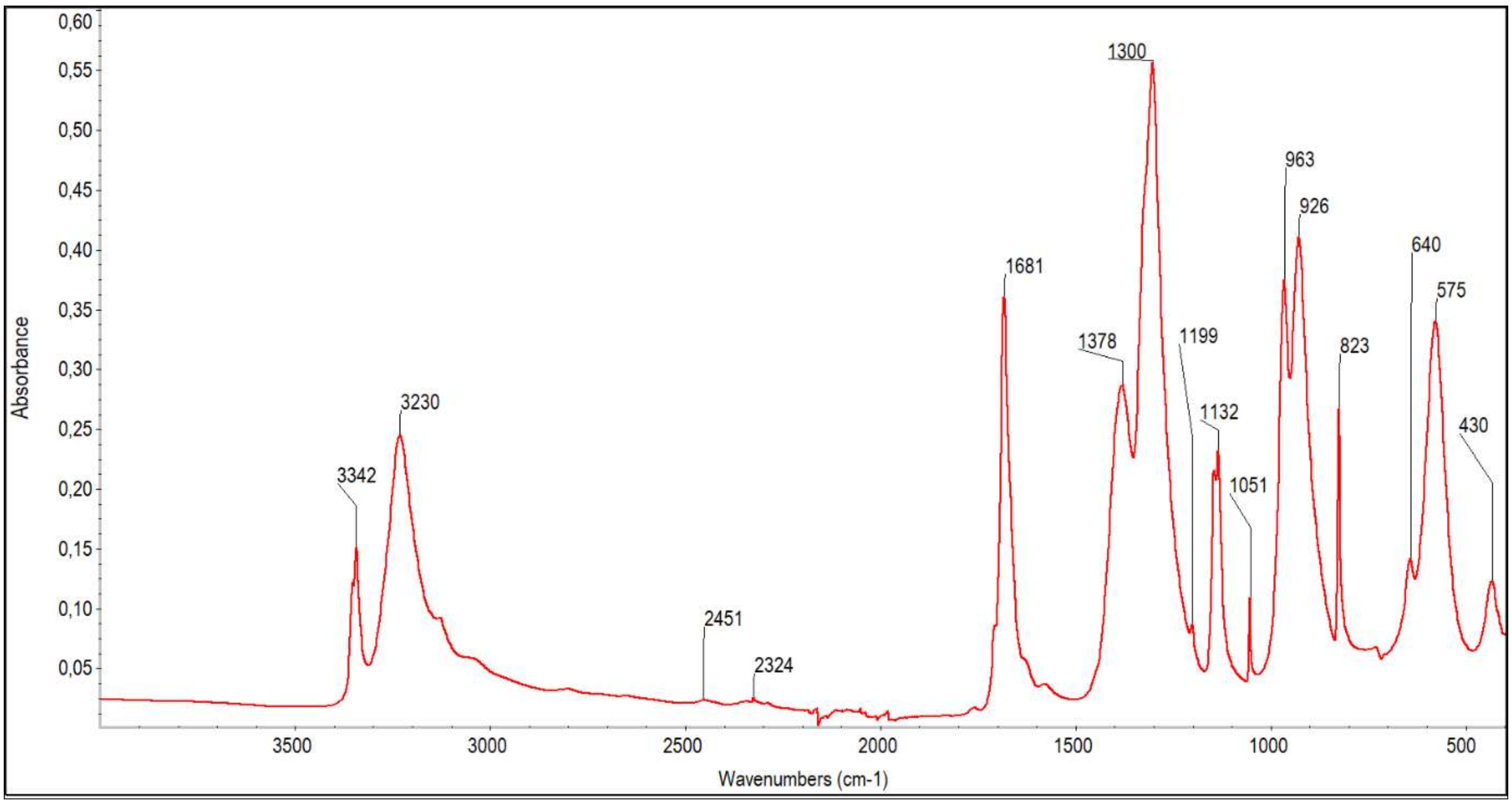

Figure S13. FTIR spectrum TAGN

Karl Fisher titration data

Table S6. Karl fisher titration data for investigated compounds. Water content, standard deviation, relative standard deviation, number of hydrates.

Compound	m/m [%]	S.D.	RSD	Hydrate
ImAzTz·H_2O	5.482	0.273	4.98	0.97
ATrAzTz	0.135	0.013	9.63	0.03
DATrAzTz	0.0215	0.0005	2.33	0.00
MBATrAzTz·H_2O	4.76	0.170	3.57	0.92
DAAmGTrAzTz	0.56	0.022	3.93	0.10
HMTAzTz·H_2O	4.351	0.107	2.46	1.12
ODHAzTz·3H_2O	11.57	0.214	1.85	2.58
DMBGAzTz·H_2O	-	-	-	-
GBAmHAzTz·2H_2O	8.546	0.591	6.92	1.60
GBAAmHAzTz·2H_2O	-	-	-	-

DTA records

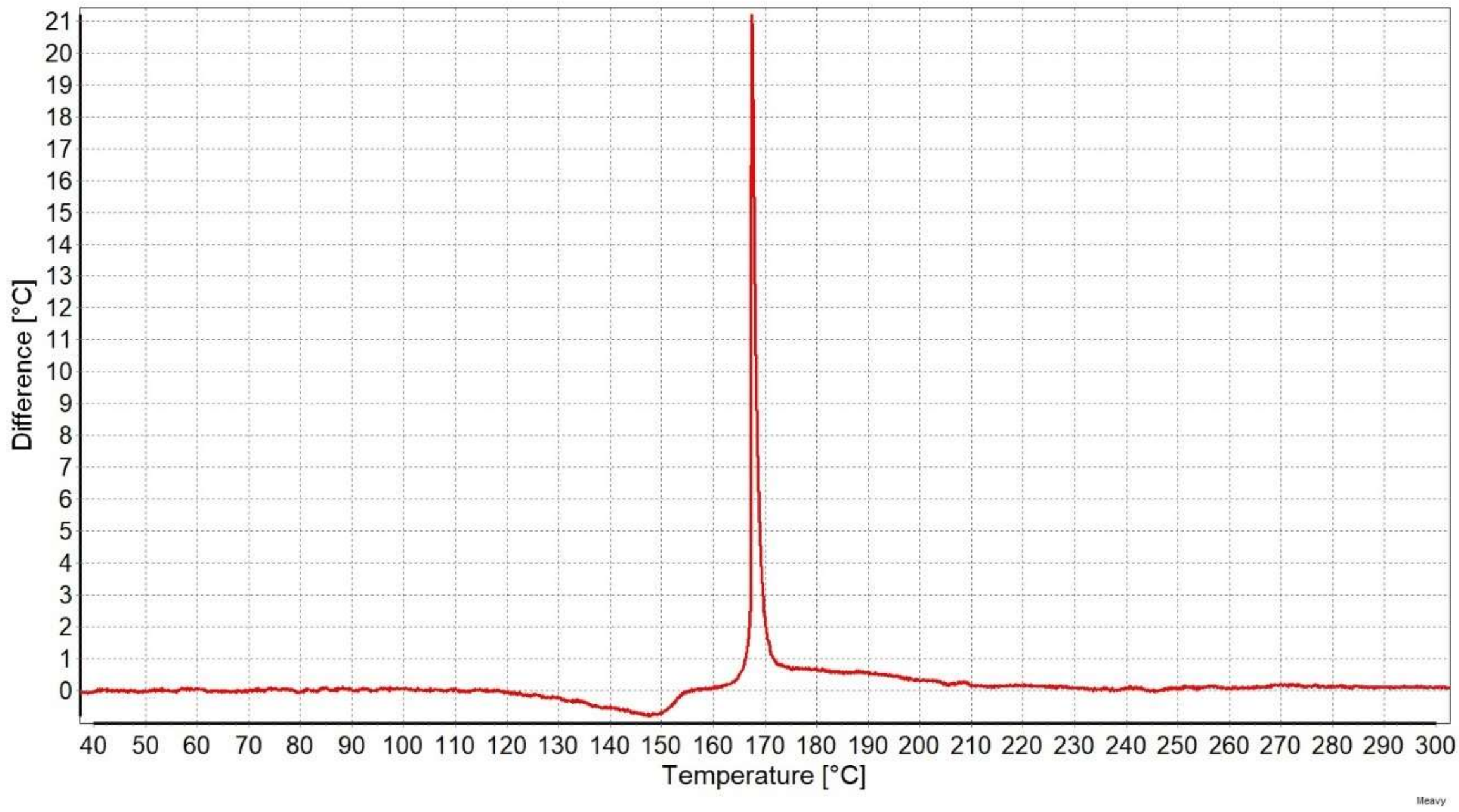

Figure S14. DTA record ImAzTZ·H_2O

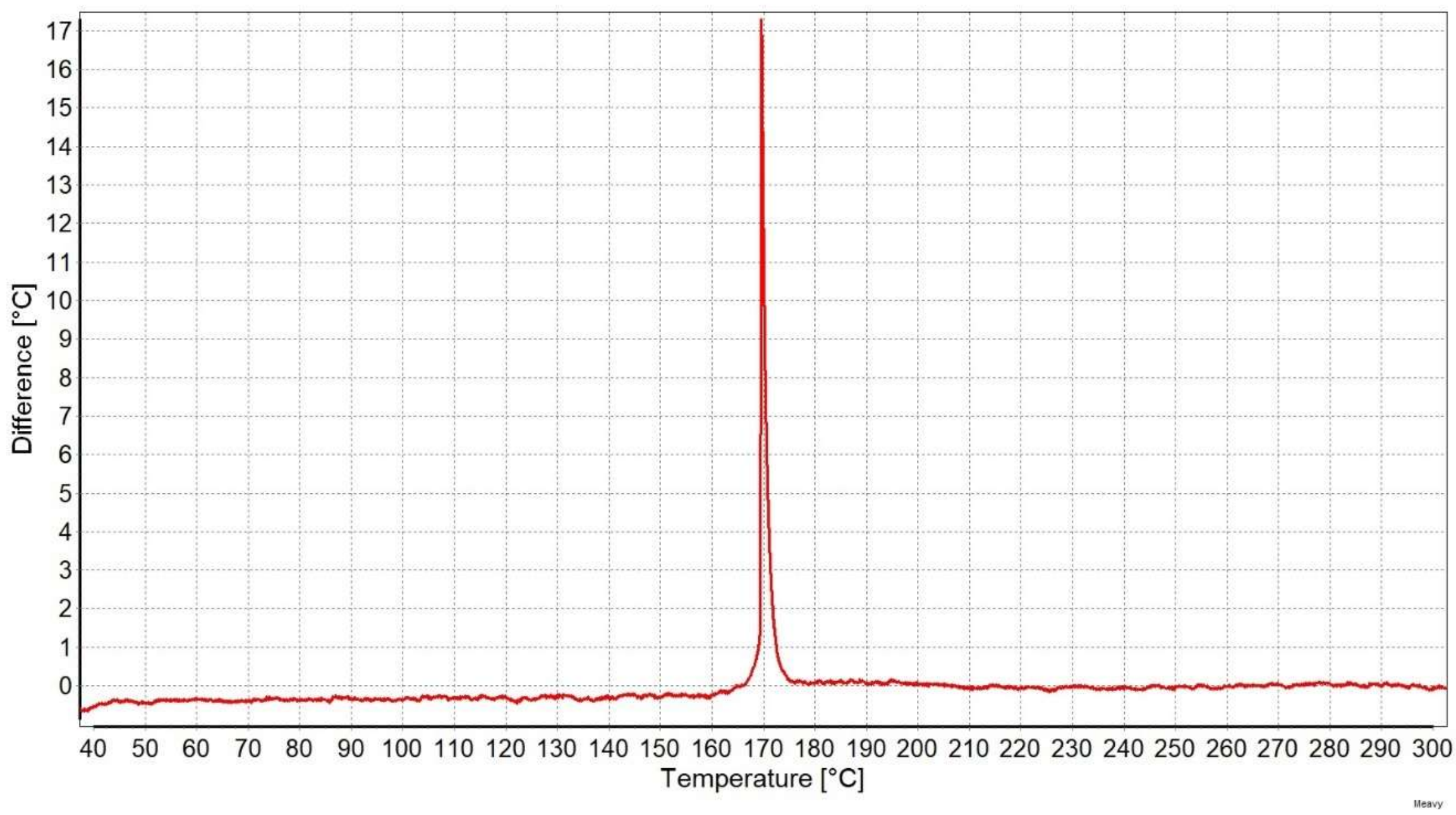

Figure S15. DTA record ATrAzTz

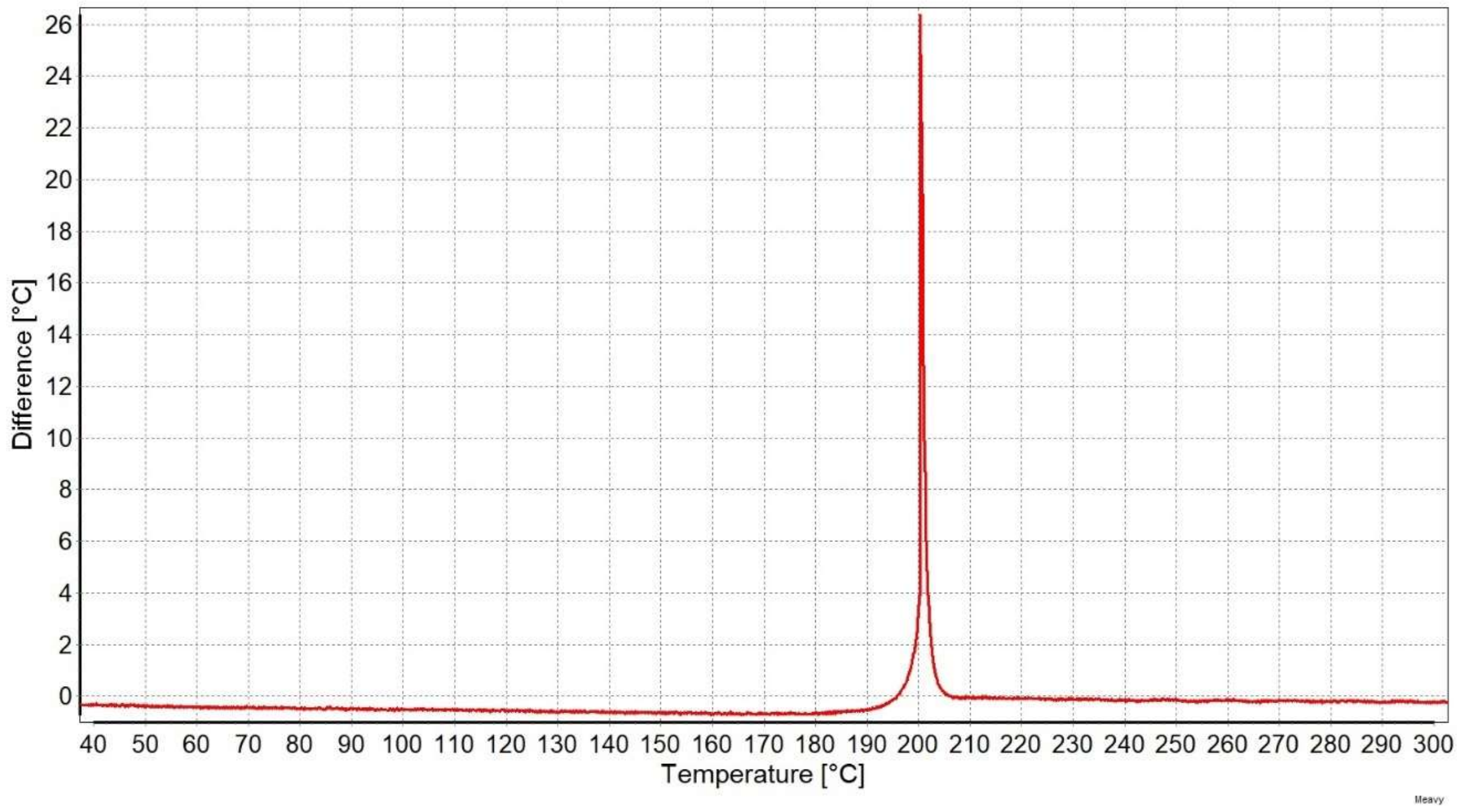

Figure S16. DTA record DATrAzTZ

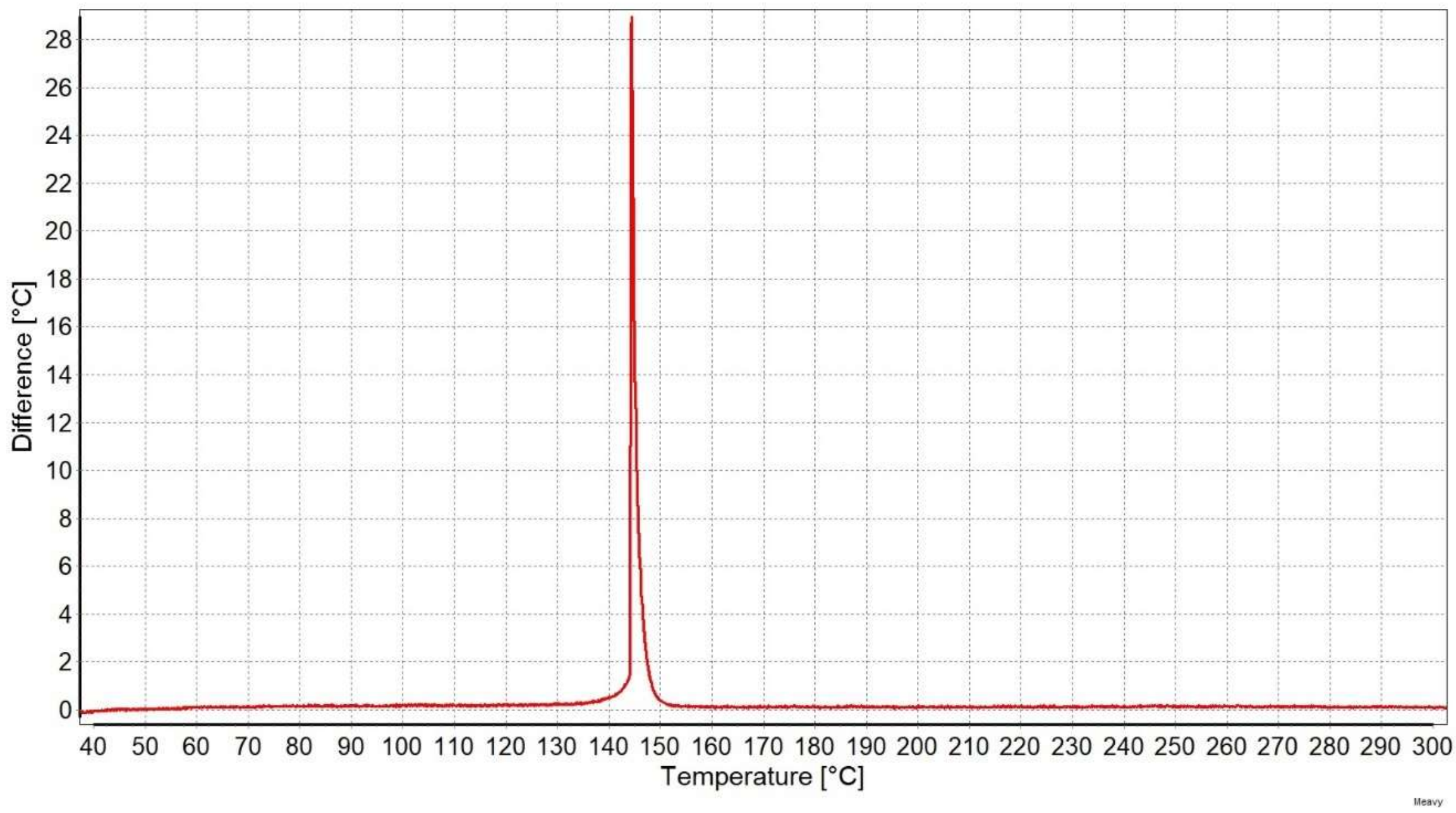

Figure S17. DTA record MBATrAzTZ·H_2O

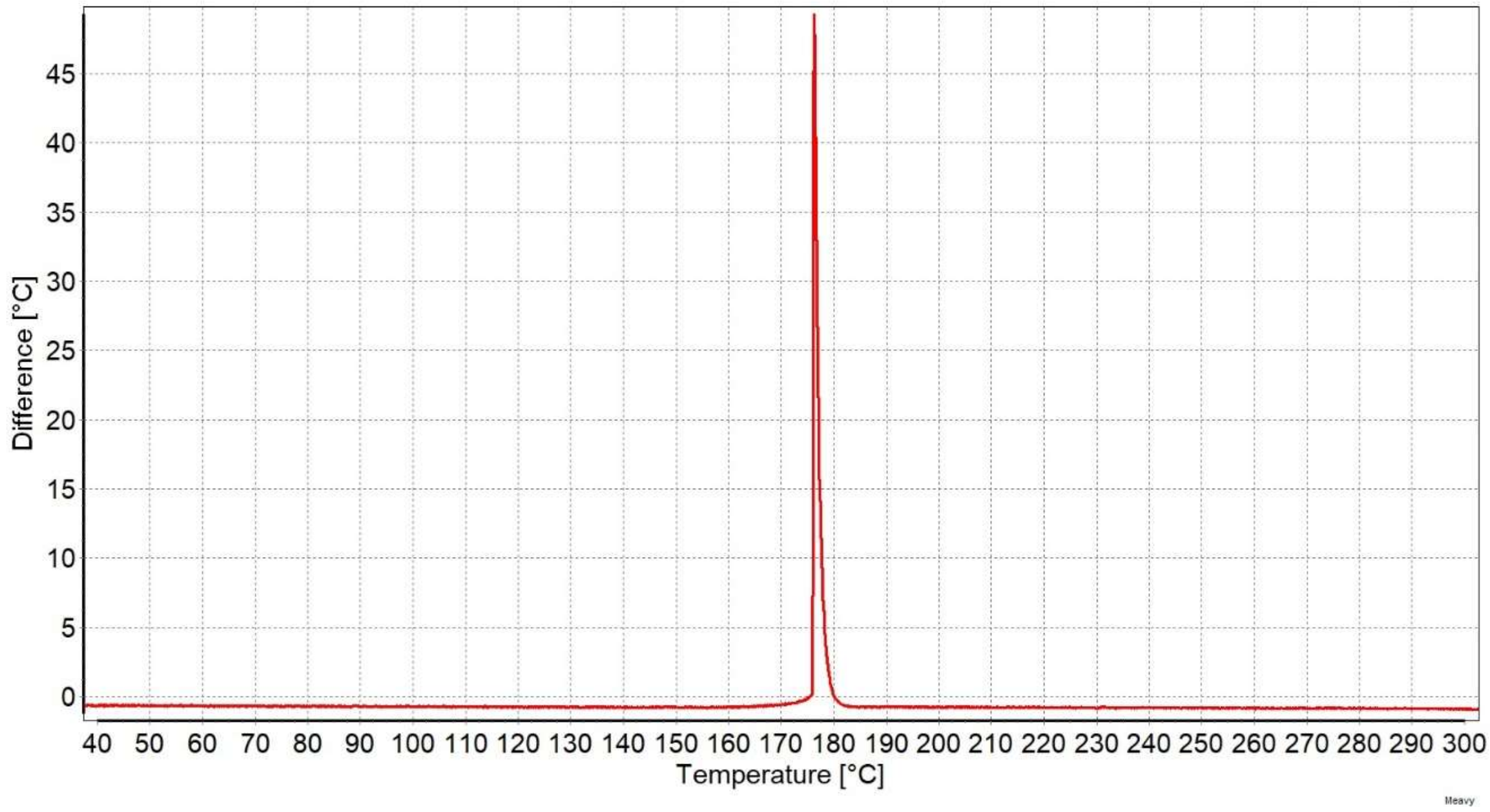

Figure S18. DTA record DAAmGTrAzTZ

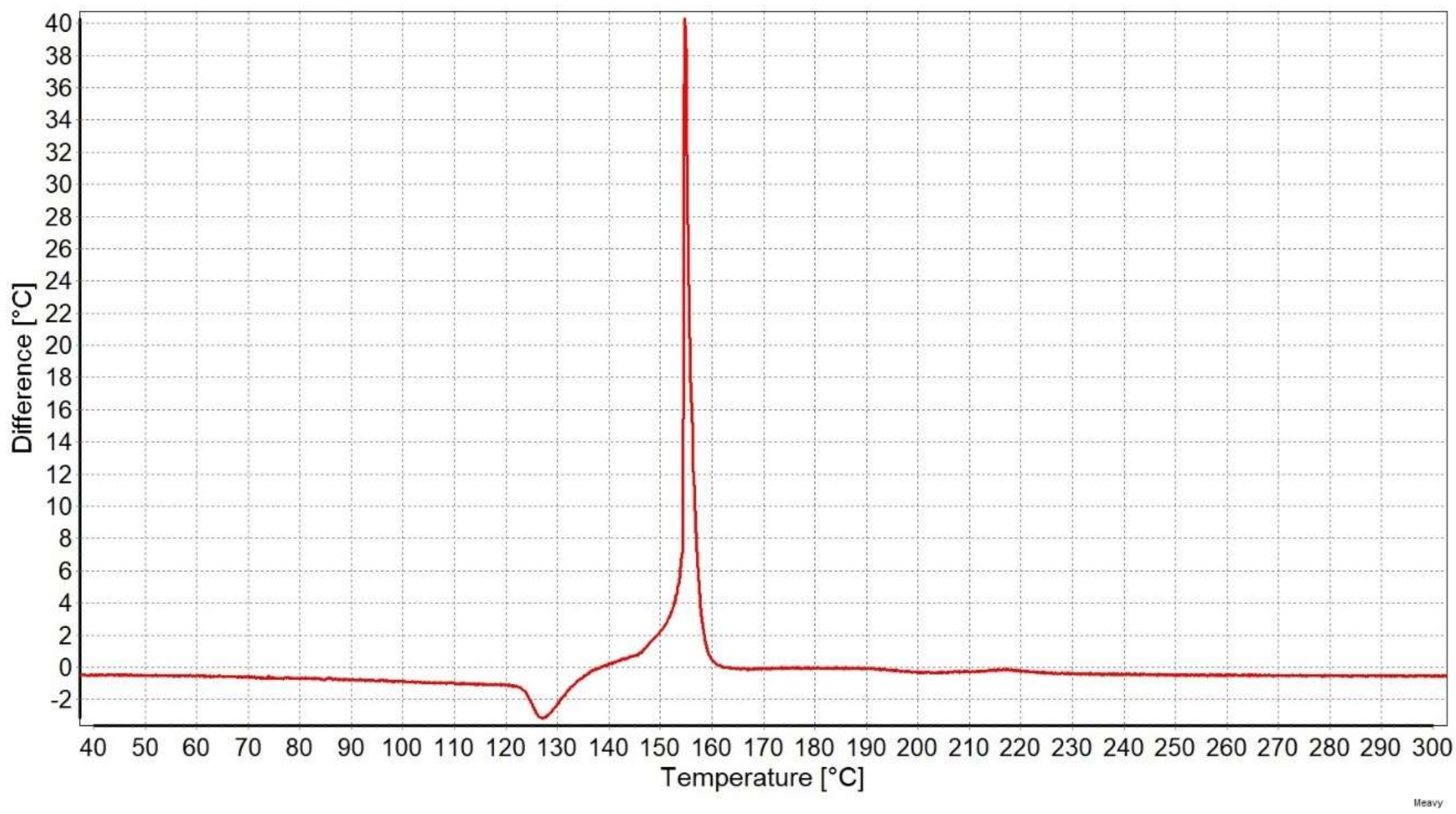

Figure S19. DTA record HMTAzTZ·H_2O

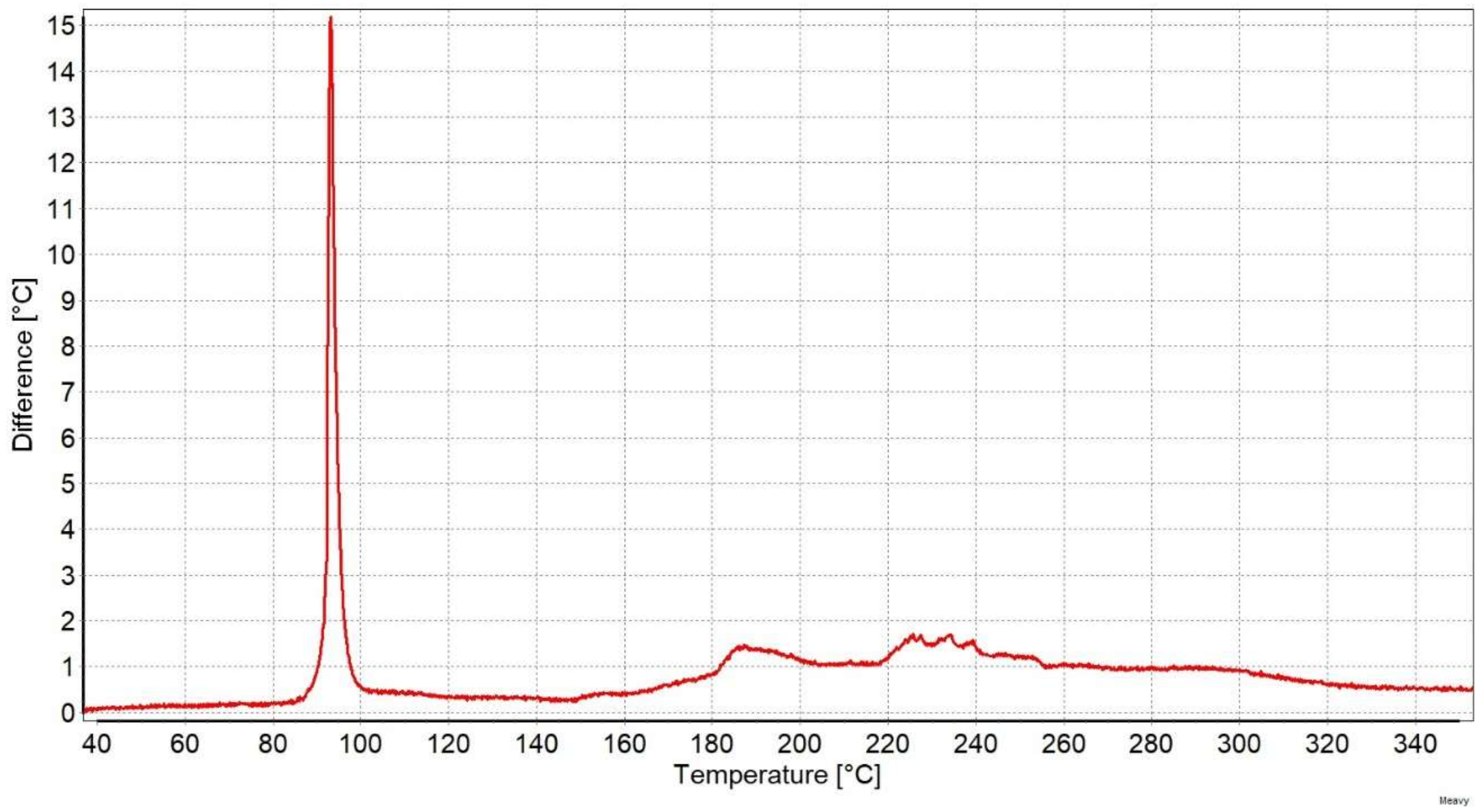

Figure S20. DTA record ODHAzTZ·3H_2O

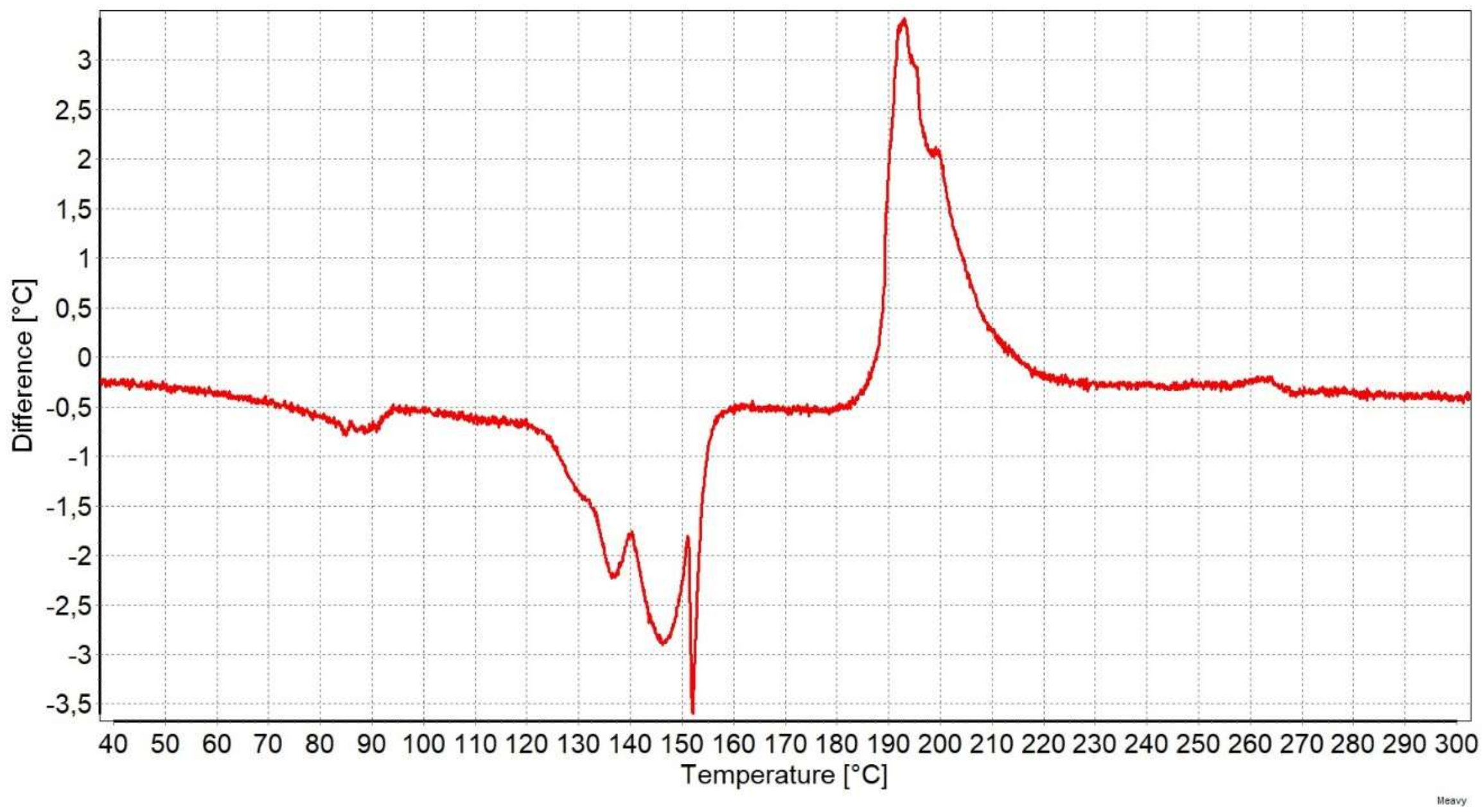

Figure S21. DTA record DMBGAzTZ·H_2O

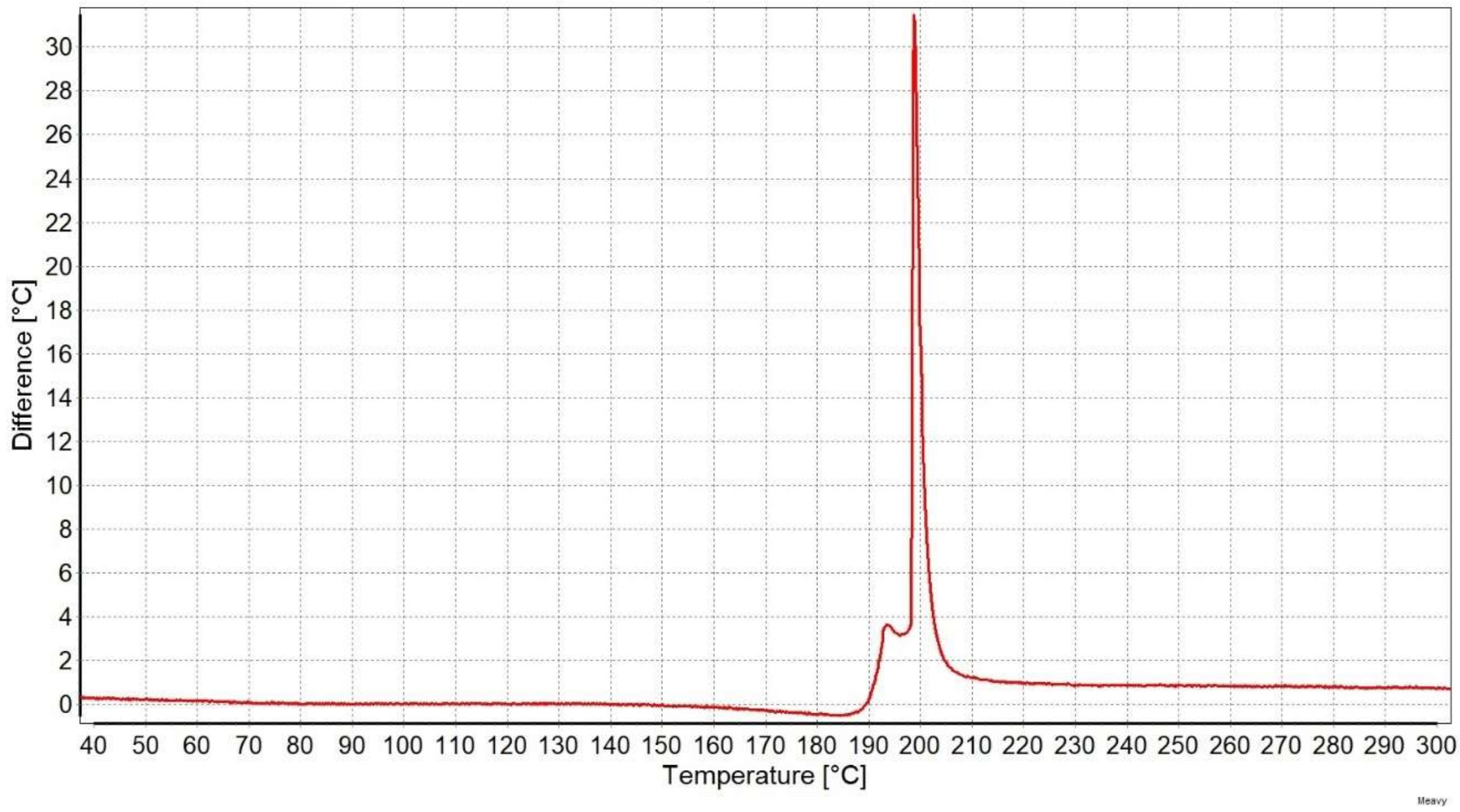

Figure S22. DTA record GBAmHAzTZ·$2H_2O$

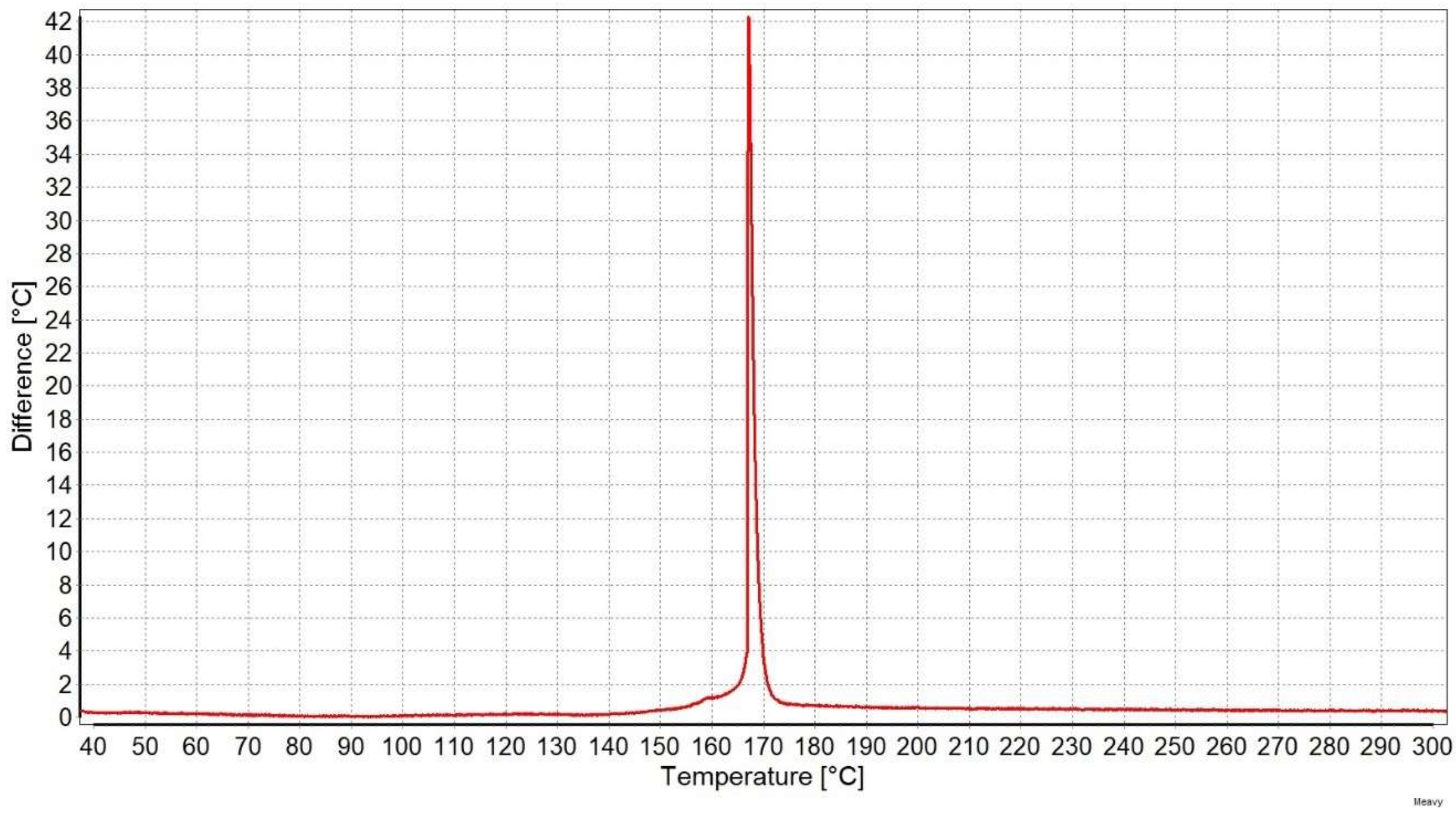

Figure S23. DTA record GBAAmHAzTZ·$2H_2O$

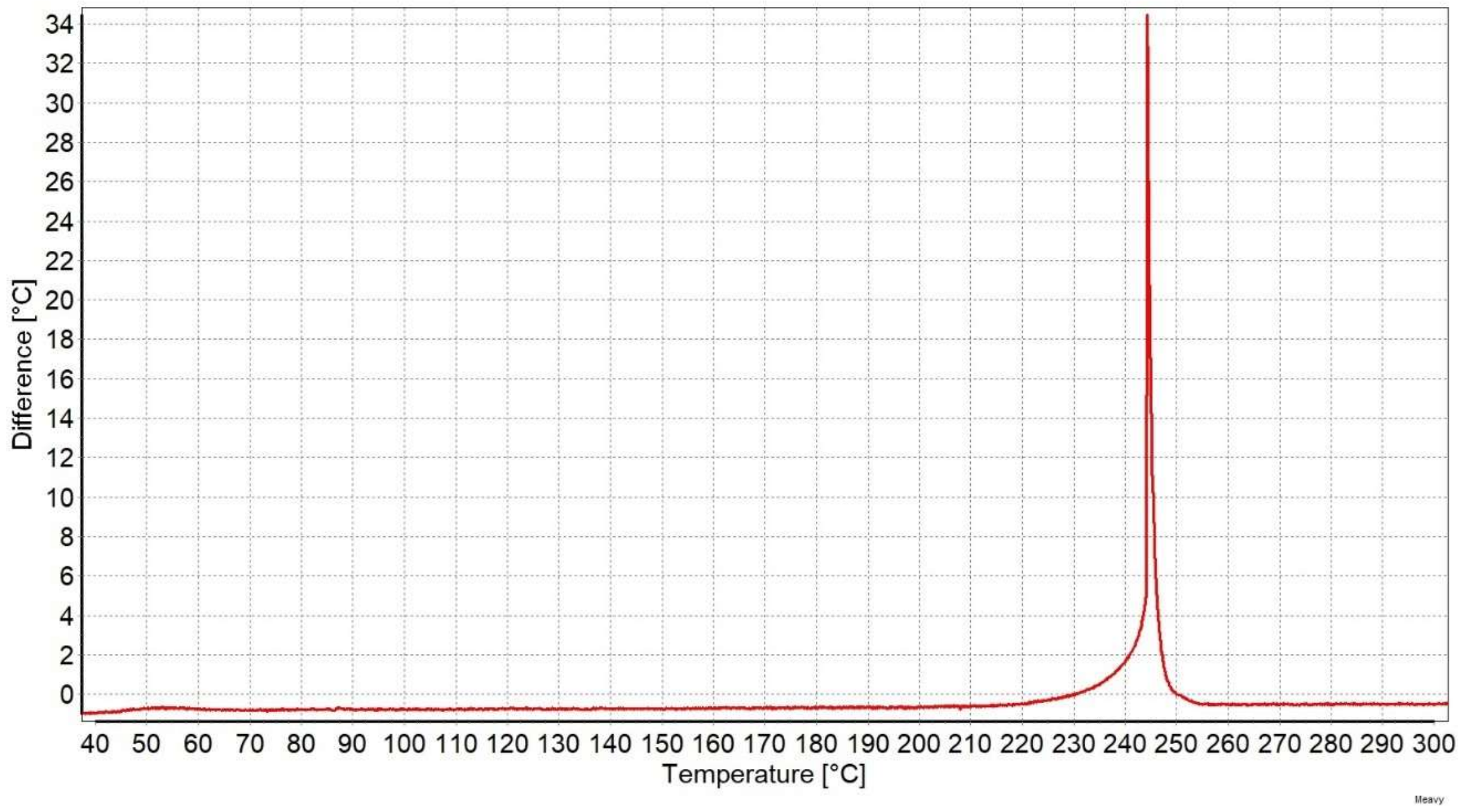

Figure S24. DTA record GZT

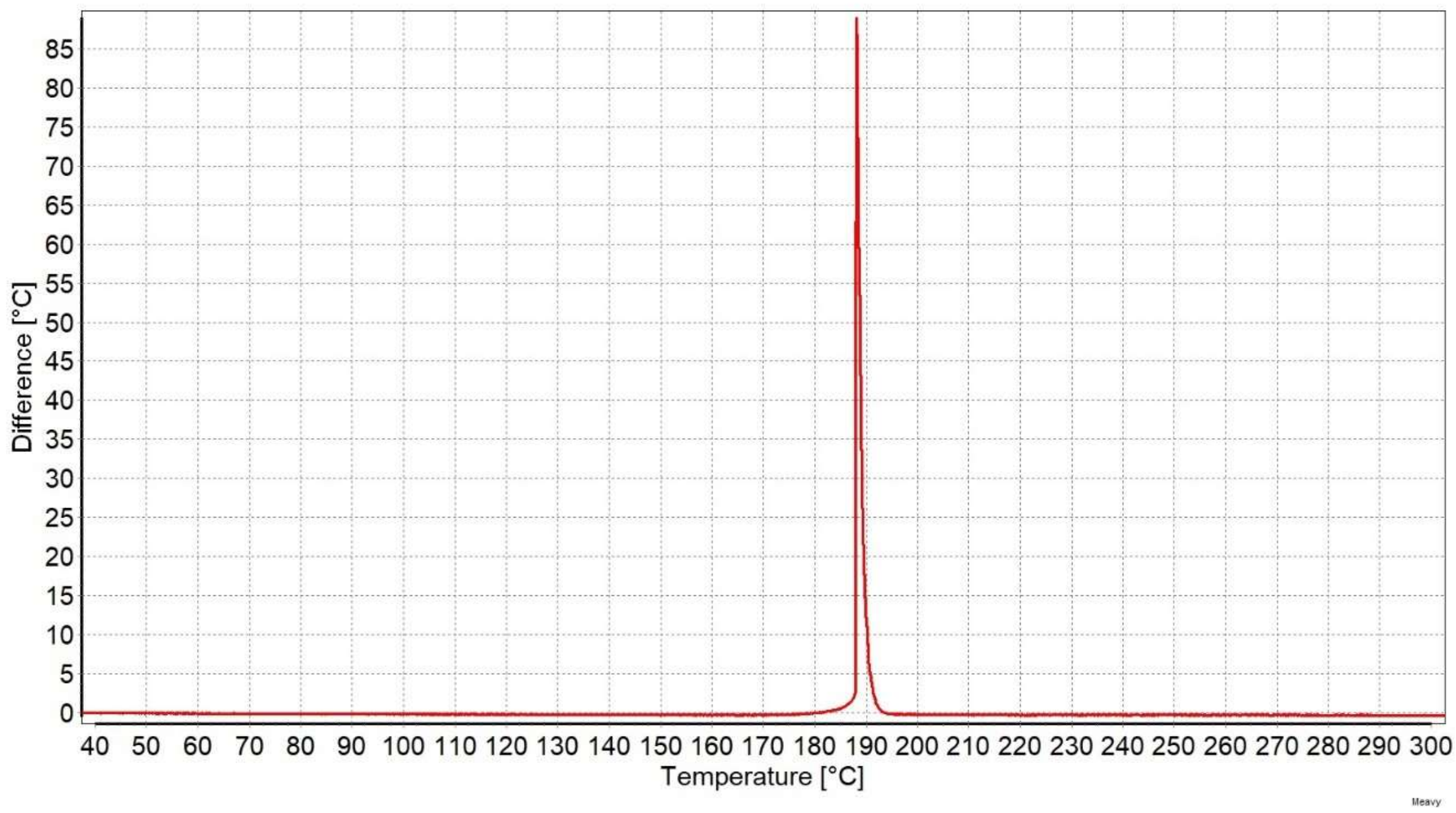

Figure S25. DTA record TAGZT

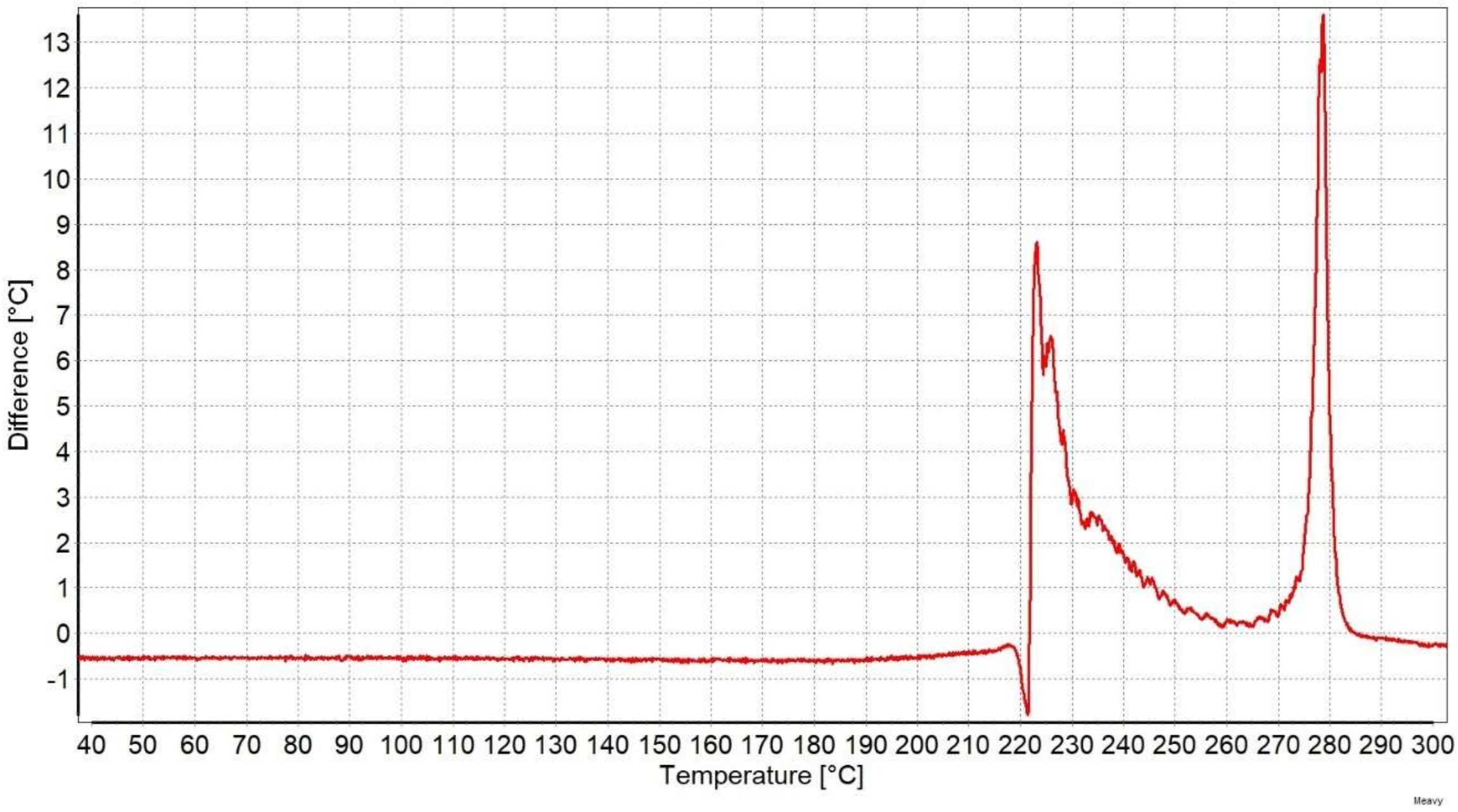

Figure S26. DTA record TAGN

Table S7. DTA Overview

**Compound**	**endotherm**		**exotherm**	
	**start [°C]**	**min [°C]**	**start [°C]**	**start [°C]**
ImAzTz·H_2O	117	148	164	167.5
ATrAzTz	-	-	163	169.5
DATrAzTz	-	-	195	200.5
MBATrAzTz·H_2O	-	-	134	144.5
DAAmGTrAzTz	-	-	171	176.5
HMTAzTz·H_2O	120	127	149	154.5
ODHAzTz·$3H_2O$	-	-	85	93
DMBGAzTz·H_2O	121	152	182	193
GBAmHAzTz·$2H_2O$	-	-	185	199
GBAAmHAzTz·$2H_2O$	-	-	160	167
GZT	-	-	215	244.5
TAGZT	-	-	180	188
TAGN	218	221.5	216	223

SEM data

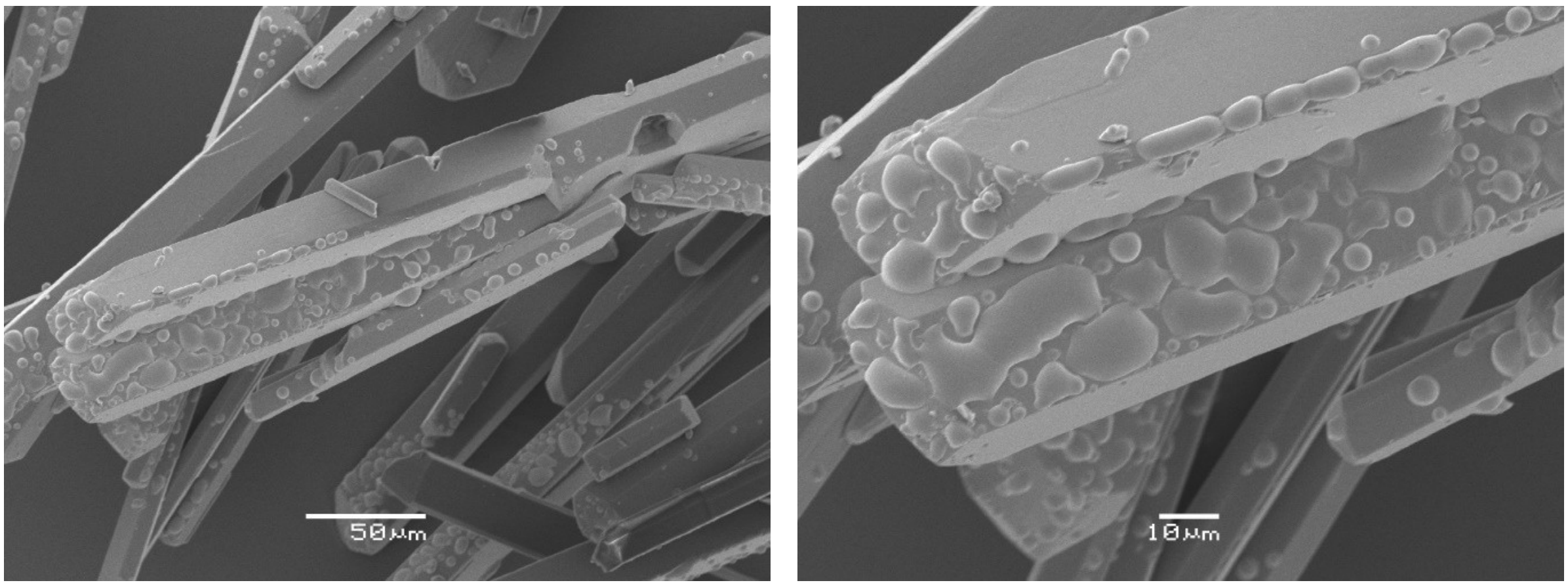

Figure S27. ImAzTZ·$2H_2O$ magnification 400x (left) and 1,000x (right)

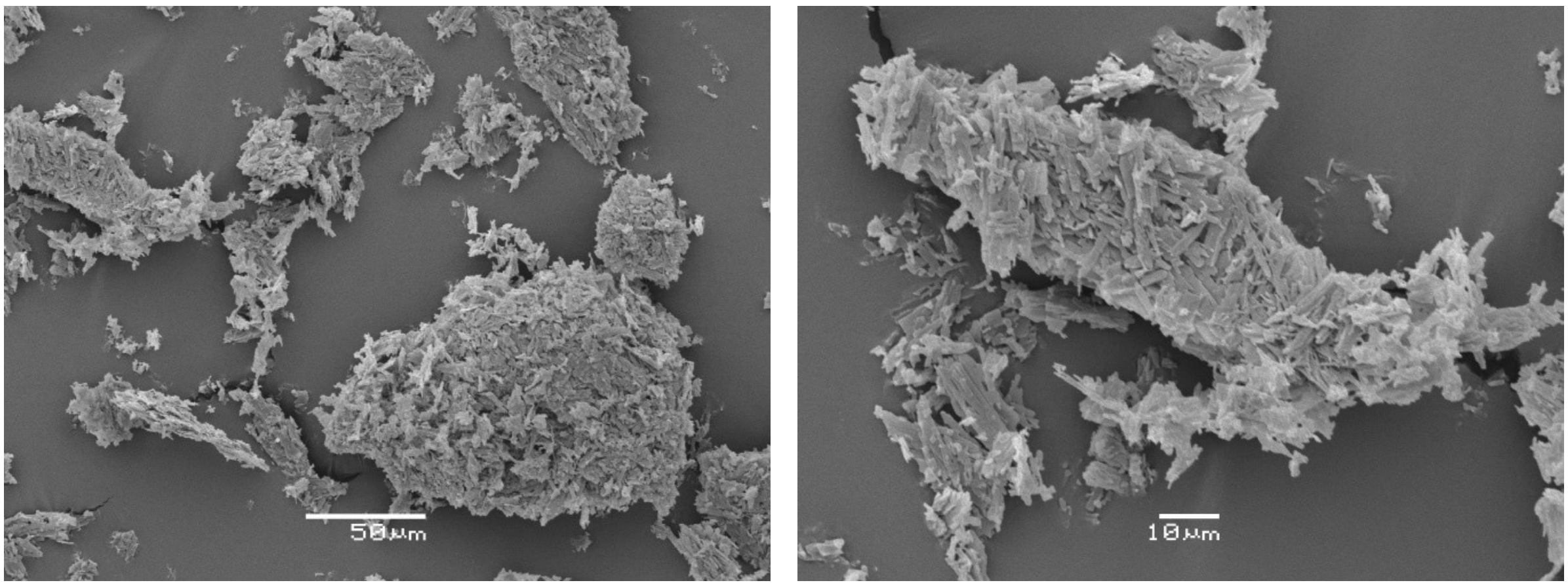

Figure S28. ATrAzTZ magnification 400x (left) and 1,000x (right)

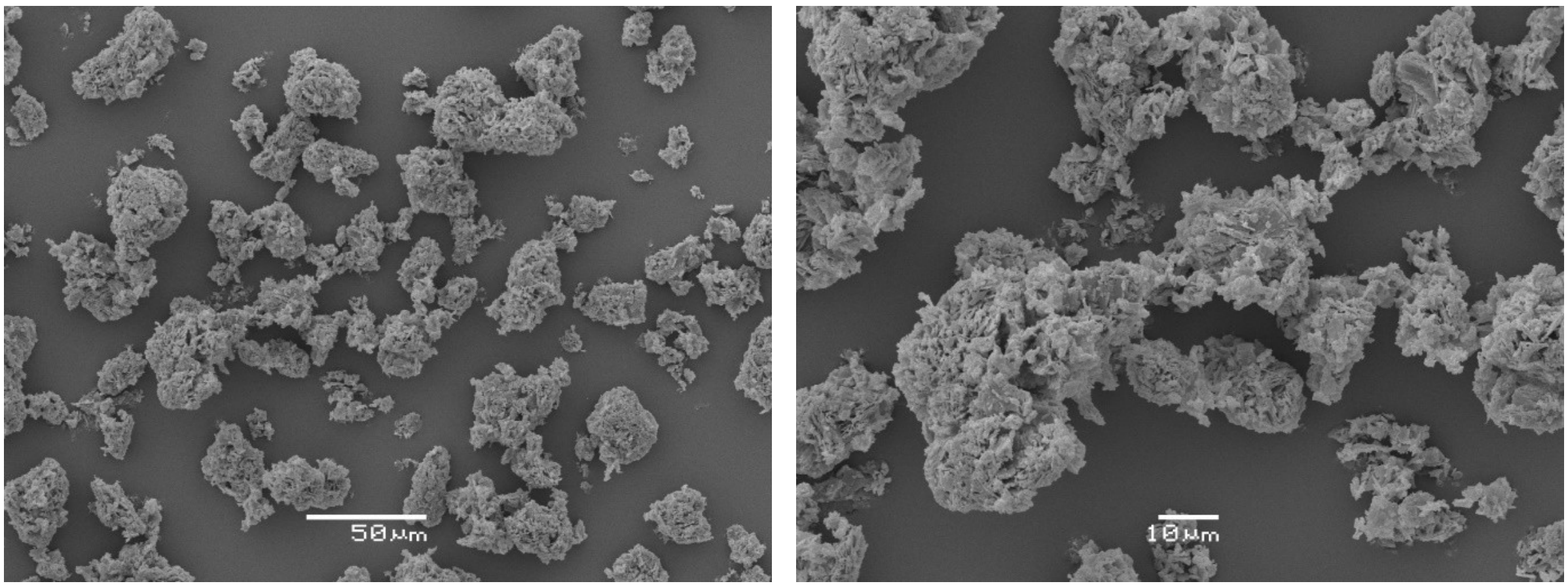

Figure S29. DATrAzTZ magnification 400x (left) and 1,000x (right)

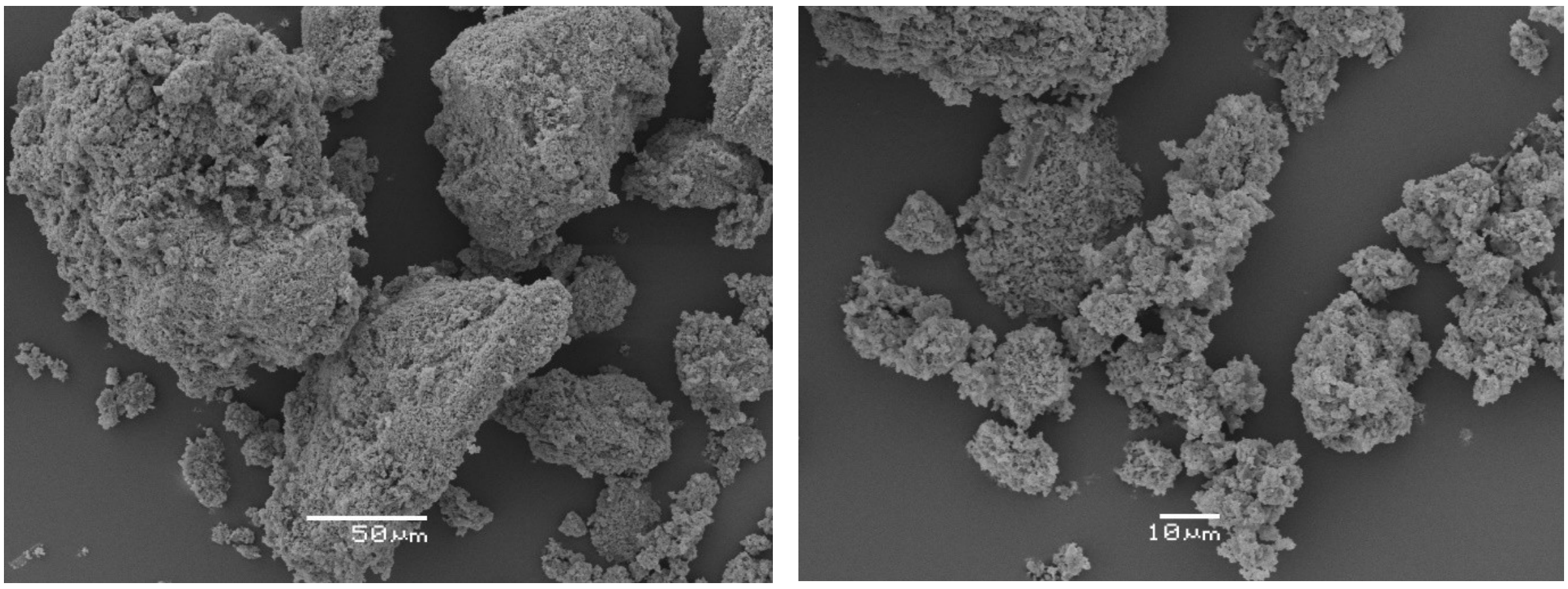

Figure S30. MBATrAzTZ·H_2O magnification 400x (left) and 1,000x (right)

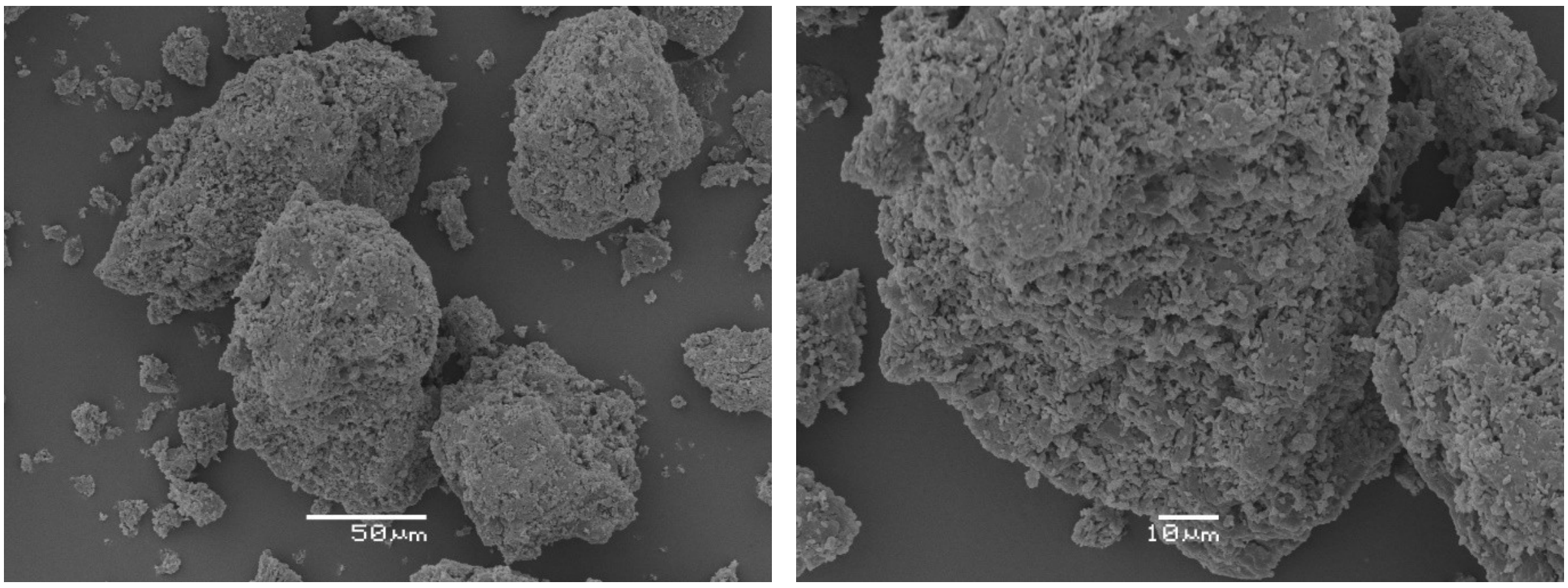

Figure S31. DAAmGTrAzTZ magnification 400x (left) and 1,000x (right)

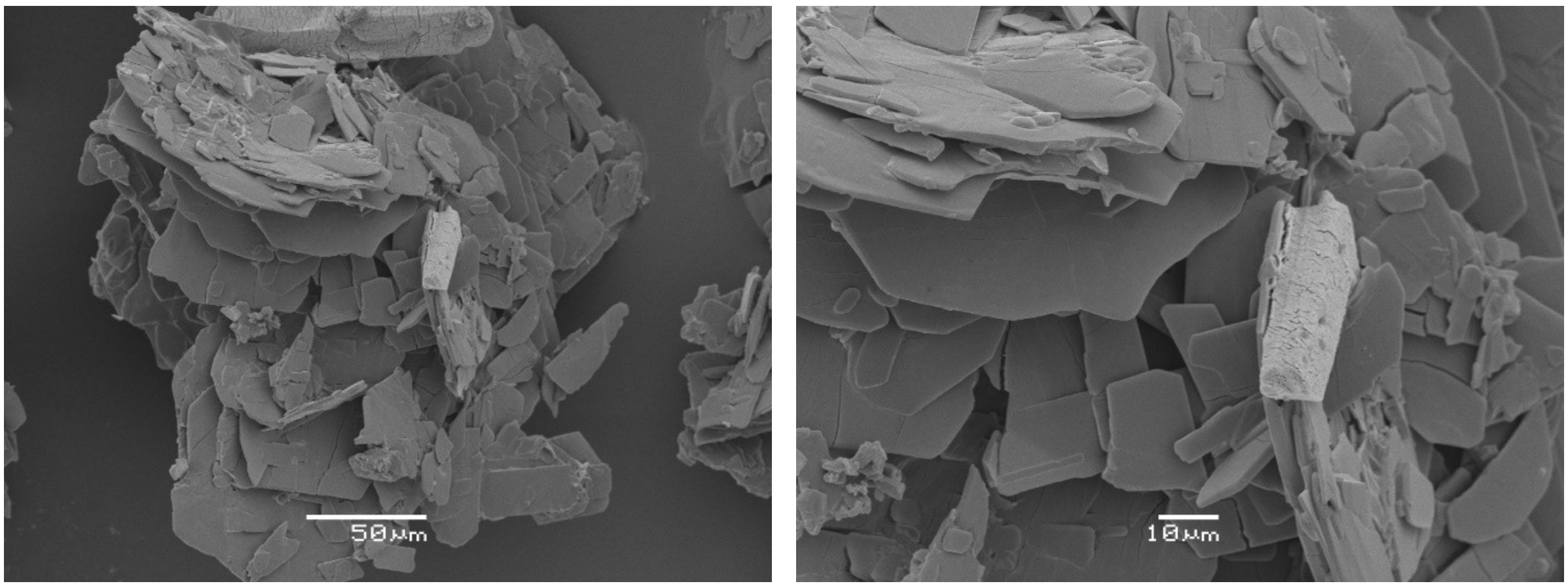

Figure S32. HMTAzTZ·H_2O magnification 400x (left) and 1,000x (right)

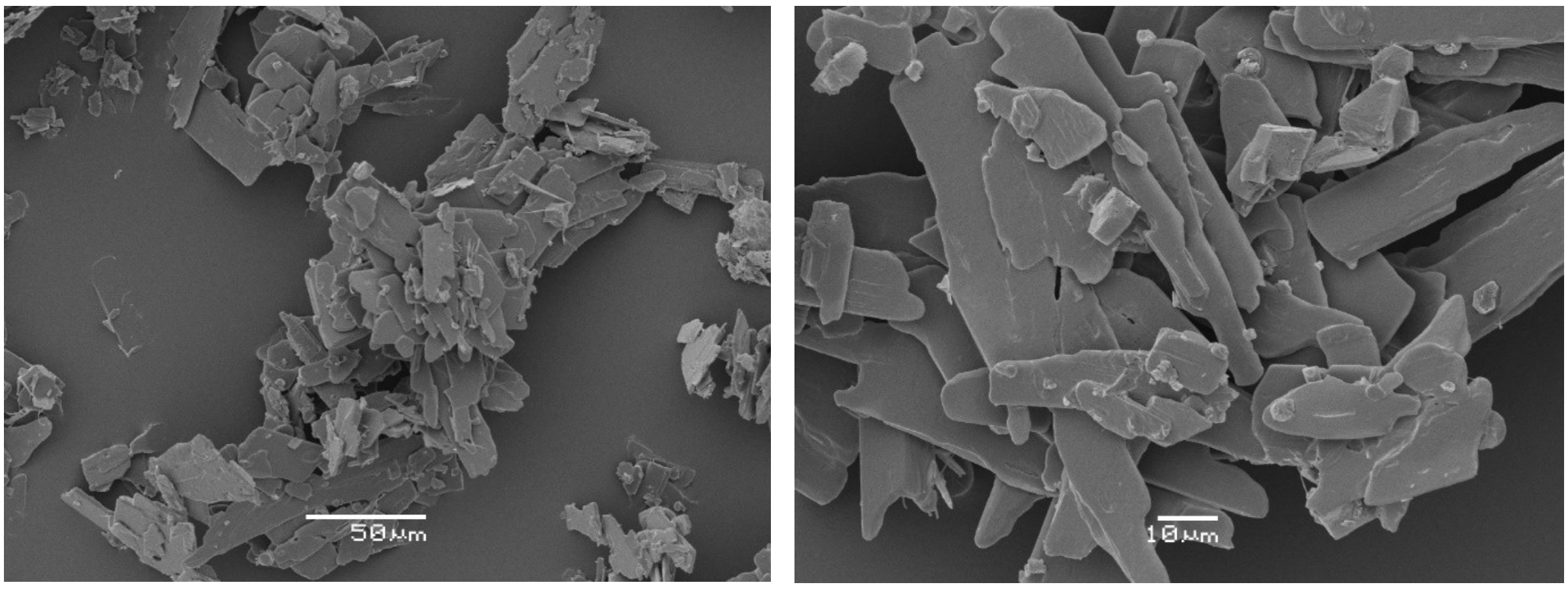

Figure S33. ODHAzTZ·$3H_2O$ magnification 400x (left) and 1,000x (right)

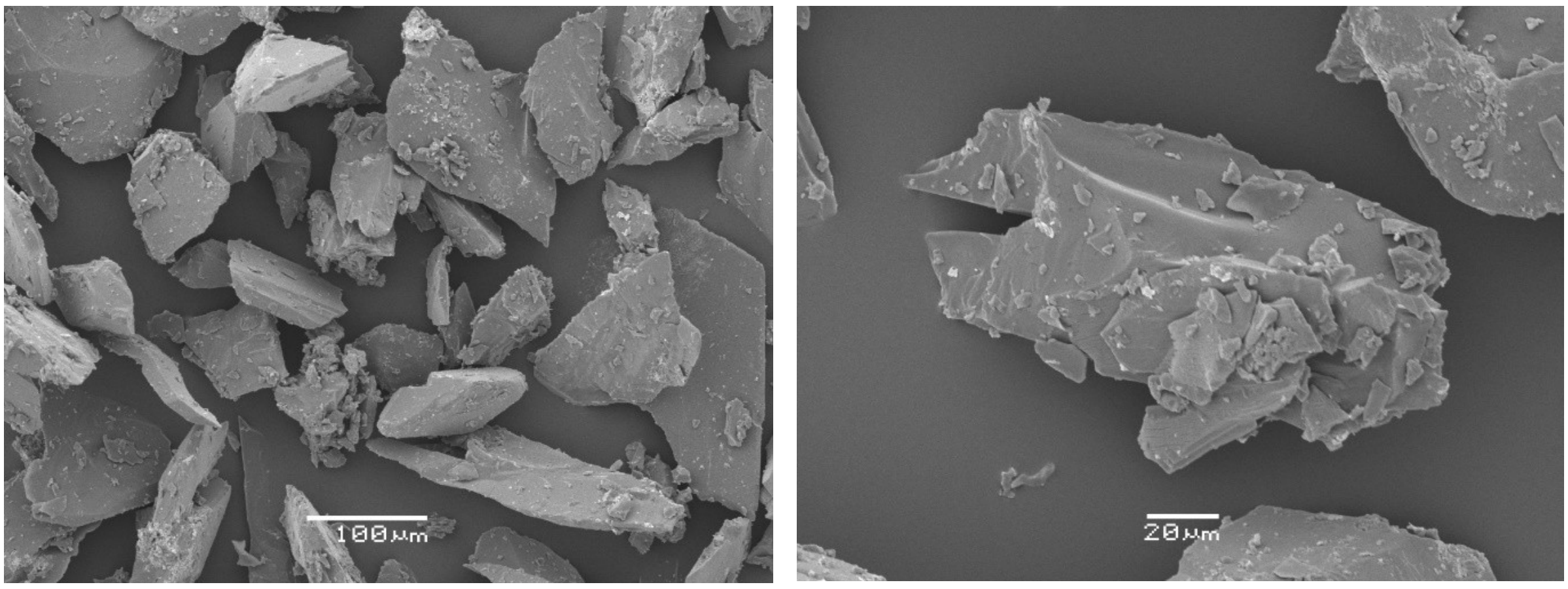

Figure S34. DMBGAzTZ·H_2O magnification 200x (left) and 600x (right)

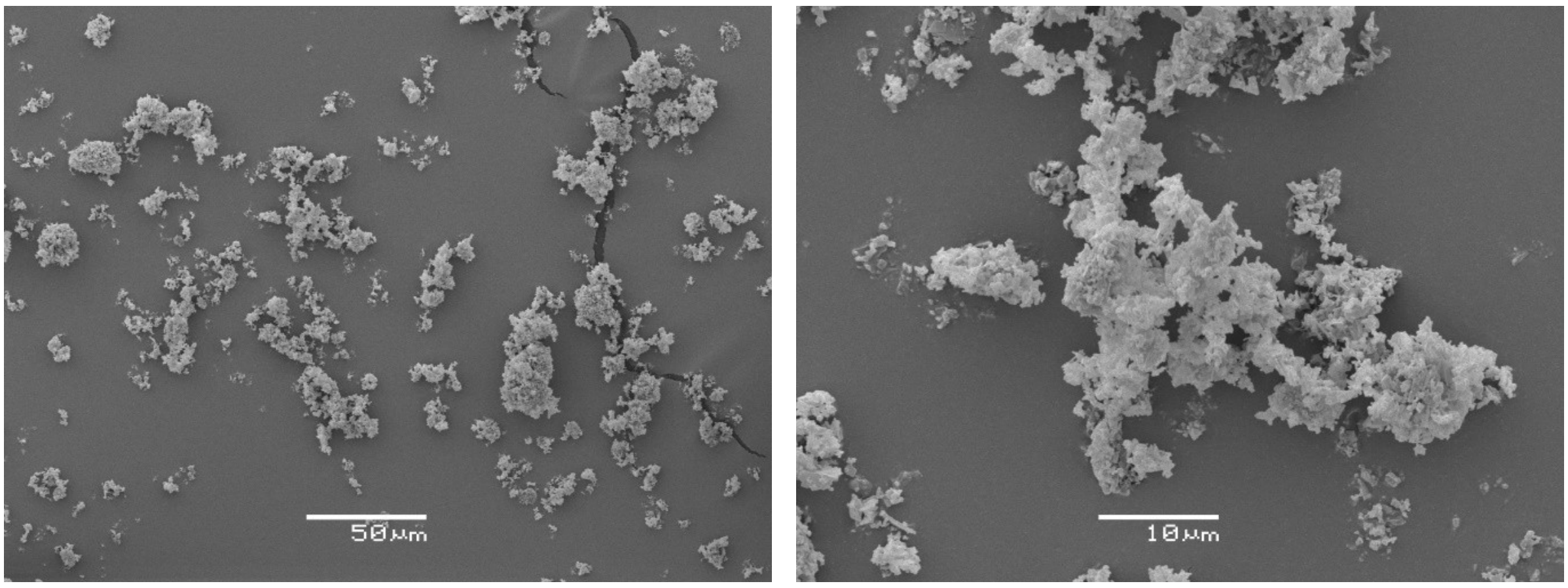

Figure S35. GBAmHAzTZ·2H_2O magnification 400x (left) and 2,000x (right)

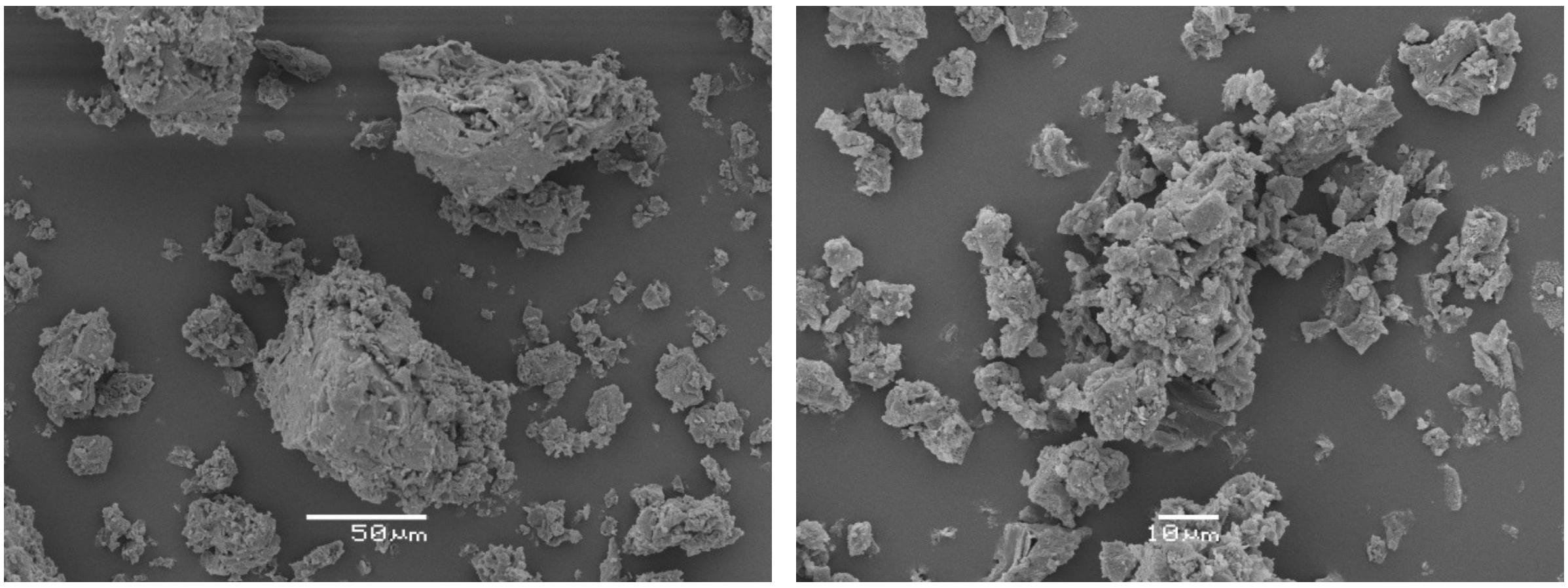

Figure S36. GBAAmHAzTZ·2H_2O magnification 400x (left) and 1,000x (right)

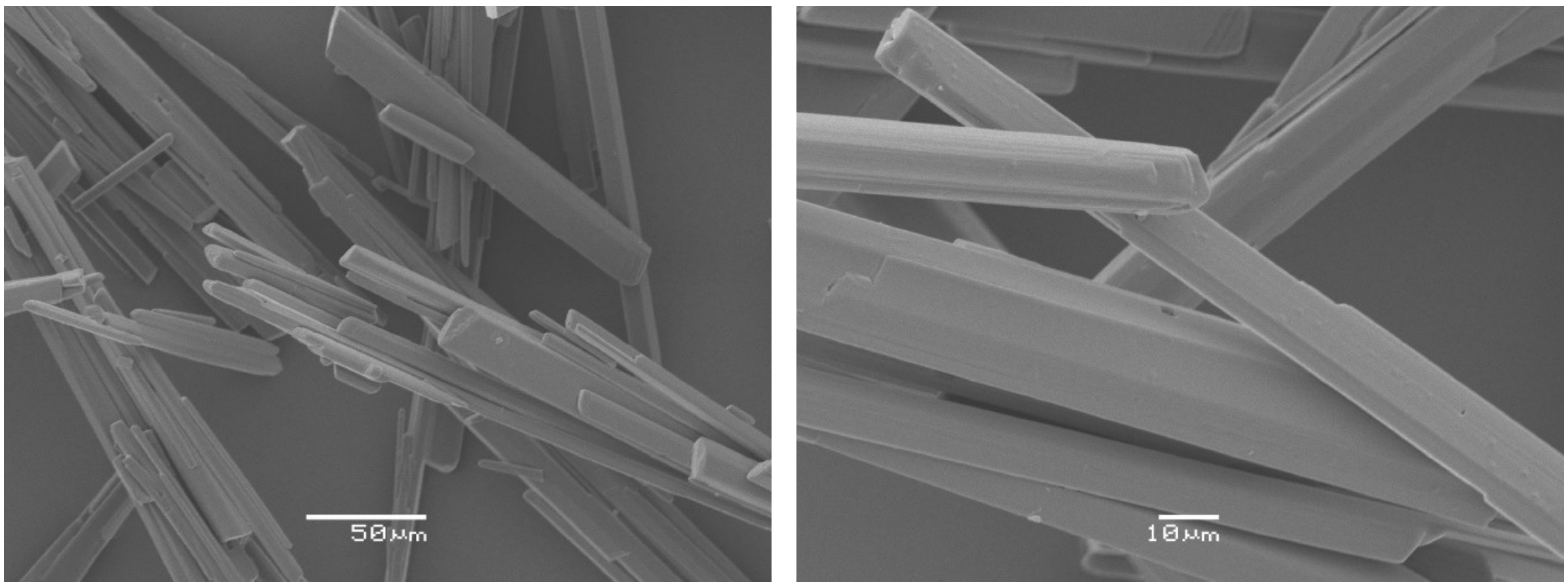

Figure S37. GZT magnification 400x (left) and 1,000x (right)

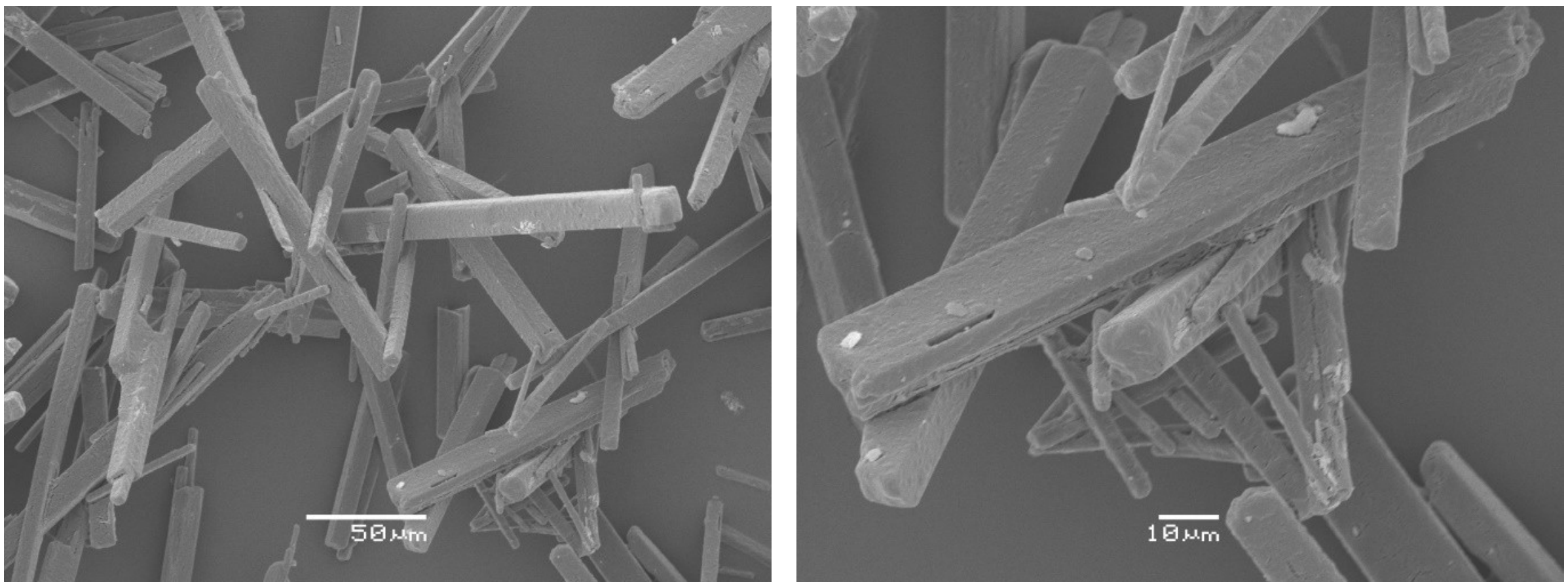

Figure S38. TAGZT magnification 400x (left) and 1,000x (right)

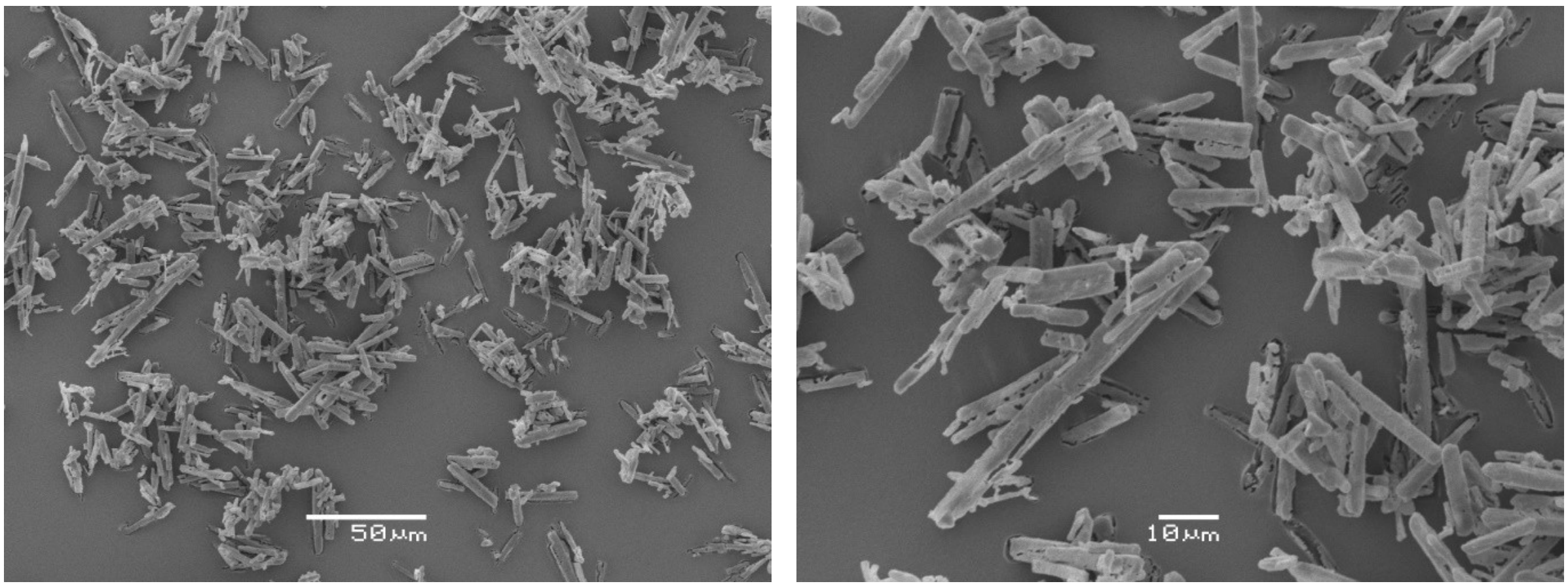

Figure S39. TAGN magnification 400x (left) and 1,000x (right)

Crystallographic data from single-crystal X-ray diffraction

Table S8. Crystallographic parameters for [1,2,4]triazolo[1,5-a][1,3,5]triazine-2,5,7-triamine.

**Crystal data**	
Chemical formula	$C_4H_6N_8$
M_r	166.17
Crystal system, space group	Orthorhombic, *Pccn*
Temperature (K)	150
a, *b*, *c* (Å)	7.7454(2), 12.5805(4), 13.1384(5)
V (Å^3)	1280.22(7)
Z	8
Radiation type	Mo*K*α
μ (mm^{-1})	0.13
Crystal size (mm)	0.39 × 0.20 × 0.04
**Data collection**	
Diffractometer	Bruker D8 - Venture
Absorption correction	Multi-scan<br>*SADABS2016*/2 - Bruker AXS area detector scaling and absorption correction Reference: Krause, L., Herbst-Irmer, R., Sheldrick G.M. & Stalke D., J. Appl. Cryst. 48 (2015) 3-10.
T_{min}, T_{max}	0.643, 0.746
No. of measured, independent and observed [$I > 2\sigma(I)$] reflections	10721, 1261, 1146
R_{int}	0.054
$(\sin \theta/\lambda)_{max}$ (Å^{-1})	0.617
**Refinement**	
$R[F^2 > 2\sigma(F^2)]$, $wR(F^2)$, S	0.095, 0.263, 1.13
No. of reflections	1261
No. of parameters	115
No. of restraints	148
H-atom treatment	H atoms treated by a mixture of independent and constrained refinement
$\Delta\rho_{max}$, $\Delta\rho_{min}$ (e Å^{-3})	1.03, −0.78

Computer programs: *SHELXL2019*/1 (Sheldrick, 2019).

Table S9. Hydrogen-bond geometry (Å, º) for [1,2,4]triazolo[1,5-a][1,3,5]triazine-2,5,7-triamine.

D—H···*A*	*D*—H	H···*A*	*D*···*A*	*D*—H···*A*
N6—H6Ba···N3^{i}	0.88	2.18	3.038(5)	164
N6—H6^{A}a···N4^{ii}	0.88	2.51	3.140(5)	129
N7—H7*A*···N5^{iii}	0.85(5)	2.17(5)	3.022(5)	174(3)
N7—H7*B*···N4^{iv}	0.97(5)	2.26(5)	3.056(5)	139(4)
N8—H8*A*···N2a^{v}	0.88	2.12	3.003(5)	179
N8—H8*B*···N3^{vi}	0.88	2.52	3.191(5)	134
N8—H8*B*···N7^{vi}	0.88	2.57	3.350(5)	149

Symmetry codes: (i) −*x*+1/2, *y*, *z*−1/2; (ii) *x*, −*y*+1/2, *z*−1/2; (iii) *x*−1/2, *y*+1/2, −*z*+1; (iv) *x*−1/2, −*y*+1, −*z*+3/2; (v) *x*+1/2, *y*−1/2, −*z*+1; (vi) −*x*+1, *y*−1/2, −*z*+3/2.

Table S10. Crystallographic parameters for **DATrAzTz**.

**Crystal data**	
Chemical formula	$C_2N_{10}\cdot 2(C_2H_5N_5)$
M_r	364.36
Crystal system, space group	Orthorhombic, *Cmca*
Temperature (K)	150
a, *b*, *c* (Å)	6.3032(10), 13.270(2), 18.199(3)
V (Å^3)	1522.2(4)
Z	4
Radiation type	Mo*K*α
μ (mm^{-1})	0.12
Crystal size (mm)	0.31 × 0.23 × 0.03
**Data collection**	
Diffractometer	Bruker D8 - Venture
Absorption correction	Multi-scan<br>*SADABS2016*/2 - Bruker AXS area detector scaling and absorption correction
T_{min}, T_{max}	0.643, 0.746
No. of measured, independent and observed [$I > 2\sigma(I)$] reflections	16309, 815, 706
R_{int}	0.073
$(\sin \theta/\lambda)_{max}$ (Å^{-1})	0.617
**Refinement**	
$R[F^2 > 2\sigma(F^2)]$, $wR(F^2)$, S	0.038, 0.108, 1.07
No. of reflections	815
No. of parameters	98
No. of restraints	87
H-atom treatment	Only H-atom coordinates refined
$\Delta\rho_{max}$, $\Delta\rho_{min}$ (e Å^{-3})	0.21, −0.22

Computer programs: Apex4 v2022.1-1 (Bruker, 2022), *SAINT* V8.40B (Bruker AXS LLC, 2019), *SHELXT* 2018/2 (Sheldrick, 2018), *SHELXL2019*/1 (Sheldrick, 2019), Bruker *SHELXTL*.

Table S11. Hydrogen-bond geometry (Å, º) for **DATrAzTz**.

D—H···*A*	*D*—H	H···*A*	*D*···*A*	*D*—H···*A*
N7—H7···N3	0.94(3)	1.92(3)	2.812(3)	158(2)
N9—H9B···N2^{i}	0.87(3)	1.99(3)	2.857(3)	179(3)
N9—H9A···N4	0.92(3)	2.20(3)	3.037(3)	152(3)
N10—H10B···N4^{ii}	0.86(3)	2.63(3)	3.483(3)	176(3)
N10—H10B···N5^{ii}	0.86(3)	2.21(3)	3.000(3)	154(3)
N10—H10A···N8^{iii}	0.83(3)	2.17(3)	2.988(2)	167(3)

Symmetry codes: (i) *x*, *y*−1/2, −*z*+1/2; (ii) −*x*+1, −*y*+1/2, *z*+1/2; (iii) −*x*+1, −*y*+1, −*z*+1.

Table S12. Crystallographic parameters for **HMTAzTz·2H$_2$O**.

**Crystal data**	
Chemical formula	$C_2N_{10}·2(C_6H_{13}N_4)·2(H_2O)$
M_r	482.56
Crystal system, space group	Triclinic, *P*-1
Temperature (K)	150
a, *b*, *c* (Å)	6.4084(2), 8.3484(2), 10.1232(3)
α, β, γ (°)	83.839(1), 89.184(1), 85.973(1)
V (Å^3)	537.12(3)
Z	1
Radiation type	Mo*K*α
μ (mm^{-1})	0.11
Crystal size (mm)	0.34 × 0.29 × 0.12
**Data collection**	
Diffractometer	Bruker D8 - Venture
Absorption correction	Multi-scan<br>*SADABS2016*/2 - Bruker AXS area detector scaling and absorption correction
T_{min}, T_{max}	0.693, 0.746
No. of measured, independent and observed [$I > 2σ(I)$] reflections	32185, 2667, 2416
R_{int}	0.047
$(\sin θ/λ)_{max}$ (Å^{-1})	0.668
**Refinement**	
$R[F^2 > 2σ(F^2)]$, $wR(F^2)$, S	0.047, 0.131, 1.08
No. of reflections	2667
No. of parameters	160
No. of restraints	131
H-atom treatment	H atoms treated by a mixture of independent and constrained refinement
$Δρ_{max}$, $Δρ_{min}$ (e Å^{-3})	0.32, −0.69

Computer programs: Apex4 v2022.1-1 (Bruker, 2022), *SAINT* V8.40B (Bruker AXS LLC, 2019), *SHELXT* 2018/2 (Sheldrick, 2018), *SHELXL2019*/1 (Sheldrick, 2019), Bruker *SHELXTL*.

Table S13. Hydrogen-bond geometry (Å, º) for **HMTAzTz·2H$_2$O**.

D—H···*A*	*D*—H	H···*A*	*D*···*A*	*D*—H···*A*
O1—H1A···N2^{i}	0.99(1)	2.39(1)	3.296(2)	152(2)
O1—H1A···N3^{i}	0.99(1)	1.94(1)	2.923(2)	173(2)
O1—H1B···N1	0.98(1)	2.70(1)	3.4985(19)	139(1)
O1—H1B···N2	0.98(1)	1.90(1)	2.857(2)	163(2)
N6—H6···N1	1.00	1.90	2.8348(16)	155

Symmetry code: (i) −*x*, −*y*+1, −*z*+1.

Table S14. Crystallographic parameters for **DMBGAzTz·2H$_2$O**

**Crystal data**	
Chemical formula	$C_2N_{10}\cdot 2(C_4H_{12}N_5)\cdot 2(H_2O)$
M_r	460.52
Crystal system, space group	Triclinic, *P*-1
Temperature (K)	150
a, *b*, *c* (Å)	5.3957(3), 9.3587(5), 11.2720(7)
α, β, γ (°)	84.617(2), 85.813(2), 73.472(2)
V (Å^3)	542.63(5)
Z	1
Radiation type	Mo*K*α
μ (mm^{-1})	0.11
Crystal size (mm)	0.59 × 0.57 × 0.12
**Data collection**	
Diffractometer	Bruker D8 - Venture
Absorption correction	Multi-scan *SADABS2016*/2 - Bruker AXS area detector scaling and absorption correction Reference: Krause, L., Herbst-Irmer, R., Sheldrick G.M. & Stalke D., J. Appl. Cryst. 48 (2015) 3-10.
T_{min}, T_{max}	0.677, 0.746
No. of measured, independent and observed [$I > 2\sigma(I)$] reflections	21742, 2678, 2416
R_{int}	0.053
$(\sin \theta/\lambda)_{max}$ (Å^{-1})	0.668
**Refinement**	
$R[F^2 > 2\sigma(F^2)]$, $wR(F^2)$, S	0.050, 0.149, 1.07
No. of reflections	2678
No. of parameters	169
No. of restraints	93
H-atom treatment	H atoms treated by a mixture of independent and constrained refinement
$\Delta\rho_{max}$, $\Delta\rho_{min}$ (e Å^{-3})	0.61, −0.59

Computer programs: Apex4 v2022.1-1 (Bruker, 2022), *SAINT* V8.40B (Bruker AXS LLC, 2019), *SHELXT* 2018/2 (Sheldrick, 2018), *SHELXL2019*/1 (Sheldrick, 2019), Bruker *SHELXTL*.

Table S15. Hydrogen-bond geometry (Å, º) for **DMBGAzTz·2H$_2$O**.

D—H···*A*	*D*—H	H···*A*	*D*···*A*	*D*—H···*A*
O1—H1A···N4	0.85(3)	1.98(3)	2.8205(18)	167(2)
O1—H1B···N3^{i}	0.74(3)	2.35(3)	3.0439(18)	156(3)
N8—H8B···N6^{ii}	0.90(2)	2.08(2)	2.9709(19)	170(2)
N8—H8A···N2	0.92(2)	2.06(2)	2.9489(19)	161(2)
N9—H9B···O1^{iii}	0.88(3)	2.21(3)	3.0590(19)	163(2)
N9—H9A···N2	0.88(3)	2.58(3)	3.258(2)	135(2)
N9—H9A···N4^{iv}	0.88(3)	2.67(2)	3.1749(19)	118.0(19)
N10—H10B···N1^{v}	0.83(2)	2.17(3)	2.9841(18)	165(2)
N10—H10A···O1^{i}	0.89(2)	2.11(2)	2.9916(18)	171(2)

Symmetry codes: (i) −*x*+2, −*y*+1, −*z*+1; (ii) −*x*+1, −*y*+1, −*z*; (iii) −*x*+1, −*y*+1, −*z*+1; (iv) *x*−1, *y*, *z*; (v) *x*−1, *y*+1, *z*.

Table S16. Crystallographic parameters for **GBAmHAzTz·4H$_2$O.**

**Crystal data**	
Chemical formula	C_2N_{10}·$C_4H_{12}N_8$·4(H_2O)
M_r	408.40
Crystal system, space group	Monoclinic, $P2_1/c$
Temperature (K)	150
a, b, c (Å)	10.1771(3), 6.8728(2), 13.3783(4)
β (°)	98.698(1)
V (Å^3)	924.99(5)
Z	2
Radiation type	Mo$K\alpha$
μ (mm^{-1})	0.12
Crystal size (mm)	0.32 × 0.19 × 0.09
**Data collection**	
Diffractometer	Bruker D8 - Venture
Absorption correction	Multi-scan<br>*SADABS2016*/2 - Bruker AXS area detector scaling and absorption correction Reference: Krause, L., Herbst-Irmer, R., Sheldrick G.M. & Stalke D., J. Appl. Cryst. 48 (2015) 3-10.
T_{min}, T_{max}	0.717, 0.746
No. of measured, independent and observed [$I > 2\sigma(I)$] reflections	14068, 1702, 1548
R_{int}	0.037
(sin θ/λ)$_{max}$ (Å^{-1})	0.606
**Refinement**	
$R[F^2 > 2\sigma(F^2)]$, $wR(F^2)$, S	0.033, 0.088, 1.02
No. of reflections	1702
No. of parameters	163
No. of restraints	74
H-atom treatment	H atoms treated by a mixture of independent and constrained refinement
$\Delta\rho_{max}$, $\Delta\rho_{min}$ (e Å^{-3})	0.18, −0.23

Computer programs: Apex4 v2022.1-1 (Bruker, 2022), *SAINT* V8.40B (Bruker AXS LLC, 2019), *SHELXT* 2018/2 (Sheldrick, 2018), *SHELXL2019*/1 (Sheldrick, 2019), Bruker *SHELXTL*.

Table S17. Hydrogen-bond geometry (Å, º) for **GBAmHAzTz·4H$_2$O**.

D—H···*A*	*D*—H	H···*A*	*D*···*A*	*D*—H···*A*
O1—H1B···N4	0.85(1)	2.01(1)	2.8480(14)	169(2)
O2—H2A···O1	0.89(1)	1.94(1)	2.8301(14)	174(2)
O2—H2B···N3^{i}	0.85(1)	2.06(1)	2.8940(14)	171(2)
O1—H1A···N2^{ii}	0.82(1)	2.07(1)	2.8821(14)	169(1)
N9—H9B···O1^{iii}	0.890(18)	1.992(18)	2.8410(14)	159.1(15)
N6—H6···N1^{iv}	0.796(9)	2.141(9)	2.9286(15)	170.5(14)
N8—H8B···N5a^{iv}	0.862(19)	2.165(19)	3.021(3)	171.9(16)
N8—H8B···N5Ab^{iv}	0.862(19)	2.143(19)	2.973(3)	161.7(15)
N8—H8A···O2	0.863(19)	1.966(19)	2.7985(14)	161.7(16)
N9—H9A···O2^{v}	0.866(18)	2.166(18)	2.9564(14)	151.5(15)

Symmetry codes: (i) −*x*, −*y*+1, −*z*+1; (ii) *x*, −*y*+3/2, *z*−1/2; (iii) −*x*, *y*−1/2, −*z*+1/2; (iv) −*x*+1, −*y*+1, −*z*+1; (v) *x*, −*y*+1/2, *z*−1/2.

Heat of combustion and heat of formation data

Table S18. Raw experimental measured heat of combustion data of *N*-rich salts of 5,5'-azotetrazole and reference gas-generating substances triaminoguanidine nitrate (TAGN), guanidinium 5,5'-azotetrazolate (GZT) and triaminoguanidinium 5,5'-azotetrazolate (TAGZT). Heat of combustion, Heat of combustion average value, Heat of formation.

**Compound**	**Heat of combustion**	**Heat of combustion average**	**Heat of formation**	**Heat of formation**
	Q_c (J/g)	***Q_c (J/g)***	***ΔH (kJ/mol)***	***ΔH (kJ/kg)***
**ImAzTz·H_2O**	17375<br>17372<br>17366	17371	712.3	2224
**ATrAzTz**	14304<br>14571<br>14455	14443	1054	3154
**DATrAzTz**	13410<br>13392<br>13413	13405	825.3	2266
**MBATrAzTz·H_2O**	14265<br>14252<br>14259	14259	741.7	2036
**DAAmGTrAzTz**	13416<br>13468<br>13487	13457	739.9	2246
**HMTAzTz·H_2O**	20703<br>20811<br>20757	20757	138.3	297.6
**ODHAzTz·3H_2O**	9786<br>9776<br>9781	9781	-736.1	-1613
**DMBGAzTz·H_2O**	17247<br>17263<br>17258	17256	-4.763	-10.76
**GBAmHAzTz·2H_2O**	13669<br>13531<br>13443	13548	411.9	1106
**GBAAmHAzTz·2H_2O**	13621<br>13607<br>13624	13617	563.0	1399
GZT	-	-	410.0	1442 [34]
TAGZT	-	-	1062	2850 [34]

TAGN	-	-	-48.12	-288 [35]

Hygroscopicity data

Table S19. Raw hygroscopicity data of *N*-rich salts of 5,5'-azotetrazole and reference gas-generating substances triaminoguanidine nitrate (TAGN), guanidinium 5,5'-azotetrazolate (GZT) and triaminoguanidinium 5,5'-azotetrazolate (TAGZT). Relative humidities, number of days, gain weight.

Compound	Relative humidity 64 %		Relative humidity 84 %	
	1. day	20. day	1. day	20. day
	Gain weight (%)			
**ImAzTz·H_2O**	0.186	0.188	0.448	0.526
**ATrAzTz**	0.141	0.121	0.177	0.216
**DATrAzTz**	0.187	0.187	0.070	0.071
**MBATrAzTz·H_2O**	0.161	0.181	1.150	1.308
**DAAmGTrAzTz**	0.202	0.141	3.320	3.947
**HMTAzTz·H_2O**	4.318	4.603	8.051	10.552
**ODHAzTz·3H_2O**	0.042	0.021	1.635	1.635
**DMBGAzTz·H_2O**	0.078	0.078	0.073	0.074
**GBAmHAzTz·2H_2O**	0.296	0.237	1.921	2.064
**GBAAmHAzTz·2H_2O**	0.676	0.537	2.449	3.014
GZT	0.094	0.113	0.164	0.206
TAGZT	0.113	0	0	0
TAGN	0.081	0.121	0.117	0.083

Sensitivity data

Table S20. Raw impact sensitivity data of *N*-rich salts of 5,5'-azotetrazole and reference gas-generating substances triaminoguanidine nitrate (TAGN), guanidinium 5,5'-azotetrazolate (GZT) and triaminoguanidinium 5,5'-azotetrazolate (TAGZT). Impact energy levels, number of initiations, weight of used hammer and impact energy levels.

Compound	Hammer weight [kg]	Impact energy *E* [J]	No. of initiations
**ImAzTz·H_2O**	1	3.0	2/15
		3.5	6/15
		4.0	8/15
		5.0	11/15
		10	13/15
**ATrAzTz**	0.5	0.5	1/15
		0.75	3/15
		1.0	8/15
		2.0	10/15
		3.0	13/15
**DATrAzTz**	2 and 1	2.5	4/15
		3.5	7/15
		5.0	9/15
		10	11/15
		20	12/15
**MBATrAzTz·H_2O**	0.5	1.0	1/15
		1.5	3/15
		2.0	6/15
		2.5	12/15
		4.0	14/15
**DAAmGTrAzTz**	1	2.5	2/15
		3.0	5/15
		4.0	8/15
		5.0	11/15
		7.5	14/15
**ODHAzTz·3H_2O**	2 and 1	1.0	3/15
		3.0	9/15
		5.0	11/15
		10	12/15
		20	14/15
**GBAmHAzTz·2H_2O**	5	10	1/15
		20	4/15
		30	6/15
		35	4/15

		40	2/15
**GBAAmHAzTz·2H_2O**	5 and 2	5.0	2/15
		10	6/15
		15	8/15
		20	11/15
		25	13/15
**GZT**	2	3.0	2/15
		5.0	6/15
		7.5	8/15
		10	9/15
		15	11/15
		20	11/15
**TAGZT**	0.5	0.5	3/15
		1.0	7/15
		2.0	10/15
		3.0	12/15
		4.0	14/15
**TAGN**	1	4.0	3/15
		4.25	5/15
		4.5	8/15
		5.0	10/15
		6.0	13/15

Table S21. Raw friction sensitivity data of reference gas-generating substances triaminoguanidine nitrate (TAGN) and triaminoguanidinium 5,5'-azotetrazolate (TAGZT). Friction force levels, number of initiations.

Compound	Friction force *F* [N]	Number of initiations
	96	1/15
	120	4/15
**TAGN**	144	7/15
	160	10/15
	192	13/15
	120	3/15
	160	6/15
**TAGZT**	216	9/15
	240	13/15
	252	14/15

Table S22. Impact sensitivity curves parameters, mean and standard deviation, of *N*-rich salts of 5,5'-azotetrazole **2-13** compared with the sensitivity of triaminoguanidine nitrate (TAGN), guanidinium 5,5'-azotetrazolate (GZT) and triaminoguanidinium 5,5'-azotetrazolate (TAGZT).

**Compound**	**Impact energy**	**Impact sensitivity curve parameters**	
	**E_{50} (J)**	**Mean μ**	**Standard deviation σ**
**ImAzTz·H_2O**	4.25	1.446	0.5946
**ATrAzTz**	1.44	0.3653	0.7464
**DATrAzTz**	4.47	1.498	1.492
**MBATrAzTz·H_2O**	2.03	0.7079	0.4163
**DAAmGTrAzTz**	3.83	1.342	0.4370
**HMTAzTz·H_2O**	> 50	-	-
**ODHAzTz·3H_2O**	2.57	0.9443	1.340
**DMBGAzTz·H_2O**	> 50	-	-
**GBAmHAzTz·2H_2O**	36.5	3.599	0.8878
**GBAAmHAzTz·2H_2O**	12.3	2.511	0.7613
GZT	7.95	2.074	1.173
TAGZT	1.16	0.1491	0.9954
TAGN	4.57	1.520	0.2287

References

[1] L. Krause, R. Herbst-Irmer, G. M. Sheldrick, D. Stalke, "Comparison of silver and molybdenum microfocus X-ray sources for single-crystal structure determination" *Journal of Applied Crystallography* **48**, *(*2015): 3–10.

[2] G. Sheldrick, "SHELXT - Integrated space-group and crystal-structure determination" *Acta Crystallographica Section A* **71**, *(*2015): 3–8.

[3] G. Sheldrick, "Crystal structure refinement with SHELXL" *Acta Crystallographica Section C* **71**, *(*2015): 3–8.

[4] 1.Bruker-AXS APEX4 v2022.1-1., 2022.

[5] J. Šelešovský, J. Pachman, "Probit Analysis - a promising tool for evaluation of explosive's sensitivity" *Central European Journal of Energetic Materials* **7**, *(*2010): 269–278.

[6] M. Lampl, G. Laus, D. E. Braun, V. Kahlenber, K. Wurst, G. Fuhrmann, H. Schottenberger, H. Huppertz, "New crystal structures in the realm of 5,5′ -azotetrazolates" *Zeitschrift für Naturforschung B* **70**, *(*2015): 125−134.

[7] H. Fu, M. Li, M. Guo, H. Tang, T. Zhang, H. Li, "On-line Raman spectroscopy combined with multivariate curve resolution-alternating least squares (MCR-ALS) to investigate the synthesis mechanism of 3,5-diamino-1,2,4-triazole (DAT)" *Spectrochimica Acta Part A: Molecular and Biomolecular Spectroscopy* **289**, *(*2023): 122231.

[8] A. Dippold, T. M. Klapötke, M. Feller, Synthesis and energetic properties of 5,5‘-dinitrimino-3,3‘-methylene-1*H*-1,2,4-triazole and selected nitrogen rich salts, *14th Seminar on New trends in research of energetic materials*, Pardubice, Czech Republic, *(*2011): 550–560.

[9] V. M. Chernyshev, A. V. Chernysheva, V. A. Taranushich, "Optimization of the synthesis of 5-amino-1,2,4-triazol-3-ylacetic acid and bis(5-amino-1,2,4-triazol-3-yl)methane" *Russian Journal of Applied Chemistry* **82**, *(*2009): 276–281.

[10] Y. Xu, Q. Lin, P. Wang, M. Lu, "Stabilization of the pentazolate anion in three anhydrous and metal-free energetic salts" *Chemistry – An Asian Journal* **13**, *(*2018): 924–928.

[11] M. Labaj, Z. Jalový, R. Matyáš, J. Nesveda, J. Mikuláštík, A. Votýpka, "One-pot synthesis of guanidinium 5,5′ -azotetrazolate avoiding isolation of hazardous sodium 5,5′ -azotetrazolate" *Organic Process Research & Development* **28**, *(*2024): 4091−4098.

[12] K. L. Wagaman, C. F. Clark, L. D. Henderson, Method of producing triaminoguanidine nitrate, US 5,041,661, (1991).

[13] A. R. Patel, A. Venkateswara, "Crystal growth in gel media" *Bulletin of Materials Science* **4**, *(*1982): 527–548.

[14] H. K. Henisch, J. Dennis, J. I. Hanoka, "Crystal growth in gels" *Journal of Physics and Chemistry of Solids* **26**, *(*1965): 493–500.

[15] M. S. Joshi, P. Mohan, A. V. Antony, "Growth of single crystals of some complex salts in silica gels" *Bulletin of Materials Science* **2**, *(*1980): 127–133.

[16] T. M. Klapötke, A. Helmy, J. T. Lechner, E. Reinhardt, Problems and approaches for estimating the enthalpy of formation of energetic materials, in *Energetic Materials and Techniques*, (2026), pp. 89–158.

[17] A. L. R. Silva, G. P. León, M. D. M. C. Ribeiro da Silva, T. M. Klapötke, J. Reinhardt, "Enthalpy of formation of the nitrogen-rich salt guanidinium 5,5′ -azotetrazolate

(GZT) and a simple approach for estimating the enthalpy of formation of energetic C, H, N, O salts" *Thermo* **3**, (2023): 549–565.
[18] F. Neese, "The SHARK integral generation and digestion system" *Journal of Computational Chemistry* **44**, (2023): 381–396.
[19] F. Neese, "Software update: The ORCA program system—version 6.0" *WIREs Computational Molecular Science* **15**, (2025): e70019.
[20] G. Bistoni, C. Riplinger, Y. Minenkov, L. Cavallo, A. A. Auer, F. Neese, "Treating subvalence correlation effects in domain based pair natural orbital coupled cluster calculations: An out-of-the-box approach" *Journal of Chemical Theory and Computation* **13**, (2017): 3220–3227.
[21] F. Neese, A. Hansen, D. G. Liakos, "Efficient and accurate approximations to the local coupled cluster singles doubles method using a truncated pair natural orbital basis" *The Journal of Chemical Physics* **131**, (2009).
[22] F. Neese, F. Wennmohs, A. Hansen, "Efficient and accurate local approximations to coupled-electron pair approaches: An attempt to revive the pair natural orbital method" *The Journal of Chemical Physics* **130**, (2009).
[23] C. Riplinger, F. Neese, "An efficient and near linear scaling pair natural orbital based local coupled cluster method" *The Journal of Chemical Physics* **138**, (2013).
[24] C. Riplinger, P. Pinski, U. Becker, E. F. Valeev, F. Neese, "Sparse maps—A systematic infrastructure for reduced-scaling electronic structure methods. II. Linear scaling domain based pair natural orbital coupled cluster theory" *The Journal of Chemical Physics* **144**, (2016).
[25] C. Riplinger, B. Sandhoefer, A. Hansen, F. Neese, "Natural triple excitations in local coupled cluster calculations with pair natural orbitals" *The Journal of Chemical Physics* **139**, (2013).
[26] L. Hellmann, J. Neugebauer, "Automatic generation of auxiliary basis sets in spherical representation using the Cholesky decomposition" *The Journal of Physical Chemistry A* **127**, (2023): 8698–8711.
[27] P. J. Lindstrom, W. G. E. Mallard, NIST standard reference database number 69, http://webbook.nist.gov/chemistry, (2026).
[28] L. Habert, D. Mathieu, S. Quéré, M. Daniel, E. Pasquinet, "Benchmark measurements for energetic salts: A prerequisite toward improved estimation methods for lattice enthalpy" *ACS Omega* **10**, (2025): 51345–51353.
[29] L. Glasser, H. D. B. Jenkins, "The thermodynamic solvate difference rule: Solvation parameters and their use in interpretation of the role of bound solvent in condensed-phase solvates" *Inorganic Chemistry* **46**, (2007): 9768–9778.
[30] H. D. B. Jenkins, L. Glasser, "Ionic hydrates, $M_pX_q \cdot nH_2O$: Lattice energy and standard enthalpy of formation estimation" *Inorganic Chemistry* **41**, (2002): 4378–4388.
[31] G. V. Belov, "Thermodynamic analysis of combustion products at high temperature and pressure" *Propellants, Explosives, Pyrotechnics* **23**, (1998): 86–89.
[32] G. V. Belov, REAL for Windows computer modeling of complex chemical equilibrium at high pressures and temperature, 2001.
[33] J. Zigmund, P. Kohlíček, I. Varga, "Development of a green propellant" *SAFEX Newsletter* **65**, (2018): 18–25.
[34] A. Hammerl, M. A. Hiskey, G. Holl, T. M. Klapötke, K. Polborn, J. Stierstorfer, J. J. Weigand, "Azidoformamidinium and guanidinium 5,5‘-azotetrazolate salts" *Chemistry of Materials* **17**, (2005): 3784–3793.
[35] R. Meyer, J. Köhler, A. Homburg, *Explosives*, 7th Edition ed., Wiley-VCH Verlag GmbH, Weinheim, (2015), p. 5.
